# Diphosphane-Mediated Control of the Emissive Properties in [Cu(NHC)(P^P)]^+^ Complexes: TADF vs Phosphorescence

**DOI:** 10.1021/acs.inorgchem.5c03511

**Published:** 2025-10-22

**Authors:** Raquel Jiménez, Olga Crespo, M. Concepción Gimeno

**Affiliations:** Departamento de Química Inorgánica, 16765 Instituto de Síntesis Química Y Catálisis Homogénea (ISQCH). Universidad de Zaragoza-CSIC Zaragoza e-50009, Spain

## Abstract

The influence of both the skeleton and substituents of the diphosphane in the photophysical properties of [Cu­(NHC)­(P^P)]­PF_6_ TADF-phosphorescent emitters is investigated [NHC = Bz-Im-2-XPy (X = H, Cl)]. The study reveals that optimization of the diphosphane structure can boost quantum yields to as high as 85% using carbenes with simple structural frameworks or substituent wings. Remarkably, the diphosphane skeleton emerges as the most influential factor for controlling the quantum yield (Φ). None of the ΔE­(S_1_-T_1_) energy gap, steric factors (buried volume percentage), or T_1_ energy alone seem to govern the observed Φ. These insights reveal new avenues for achieving high-performance TADF emitters through ligand engineering.

## Graphical abstract

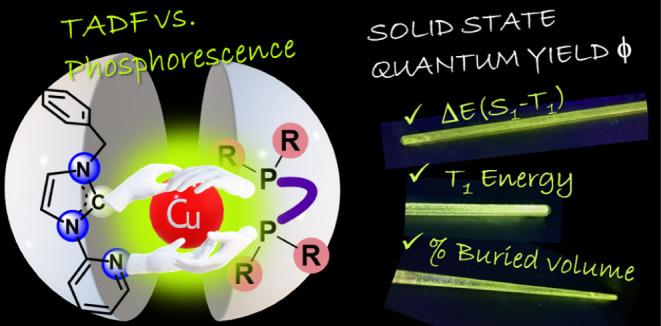

## Introduction

Thermally activated delayed fluorescence (TADF) may be considered a triplet recycling upconversion process, achieved through reverse intersystem crossing (RICS) from the excited triplet to the excited singlet, by taking advantage of a small energy gap between both states.^1,2^ The use of TADF emitters is one of the strategies that enables the increment of the internal quantum efficiency in OLEDs or LECs.^3,4^ TADF emitters may also find applications in fields like biomedicine, sensing, and catalysis.^5^ The design, study, and modification of TADF-emitting molecules, have been greatly impacted by these developments, making TADF a dynamic and rapidly evolving area of research within the photophysics and photochemistry of coordination compounds.^6,7^

A major advantage is that TADF facilitates the use of coordination complexes based on abundant and cheaper metals, unlike heavy metals that make feasible phosphorescence due to spin–orbit coupling, other triplet recycling mechanism.^8^ These findings have triggered a significant interest in copper­(I) emissive complexes.^9,10^ Among them, diphosphane/diimine tetracoordinated copper compounds [Cu­(N^N)­(P^P)]^+^ have been deeply studied with the aim of identifying the factors that may tune their emission color, lifetime, and quantum yield.^11,12^ The presence of the diphosphane as a ligand in these tetracoordinated complexes prevents distortion from tetrahedral to square planar geometry, which may lead to emissive quenching.

More recently, mixed NHC ditopic carbene/diphosphane complexes [Cu­(NHC)­(Dpephos)]^+^ have demonstrated intriguing luminescent properties, with studies exploring the impact of both the carbene wingtips and the carbene core on their emissive behavior (Figure 1)^13–24^ TADF has been proposed as the origin of emission for these complexes, although only a few studies have reported temperature-dependent lifetime modifications and the ΔE­(S_1_-T_1_) energy gap. Further noticeable are their mechanochromic properties^15^ and their use as color converters^13^ as reported for some of these compounds. However, the specific influence of the diphosphane ligand on the emission properties of these compounds remains largely unexplored, as most of the reported complexes feature the same diphosphane (Dpephos),^13–24^ with the exception of three complexes reported with Xantphos.^25^

**Figure 1:**
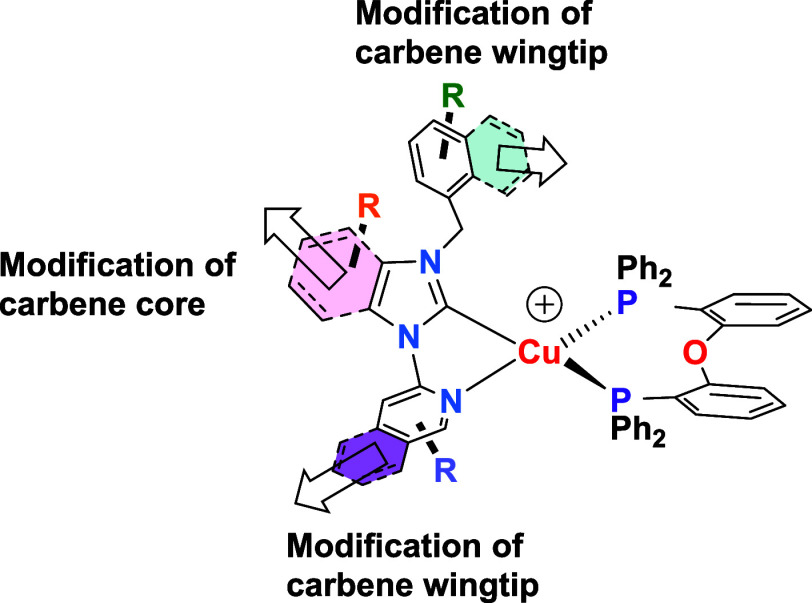
Scheme of the modification of the NHC carbene in different reported photophysical studies of [Cu­(NHC)­(Dpephos)]^+^ complexes. In this work, we focus on the diphosphane influence.

With this scenario in mind, this work is mainly focused on the analysis of the diphosphane effect in the emissive properties of [Cu­(NHC)­(P^P)]­PF_6_ complexes. With this purpose, we report the synthesis of five new complexes (**Cu1**-**Cu4** and **Cu6**), modifying both the diphosphane backbone and the substituents on the phosphorus atoms. Figure 2 shows the diphosphane and NHC ligands selected for this study. Although complexes [Cu­(NHC)­(Dpephos)]­PF_6_ (NHC = L_A_, L_B_) have been previously reported,^19,23^ we have also synthesized [Cu­(L_B_)­(Dpephos)]­PF_6_ (**Cu5**) for comparison. The analysis of the TADF behavior, steric factors (buried volume percentage), and T_1_ energies is carried out trying to understand trends in quantum yields.

**Figure 2:**
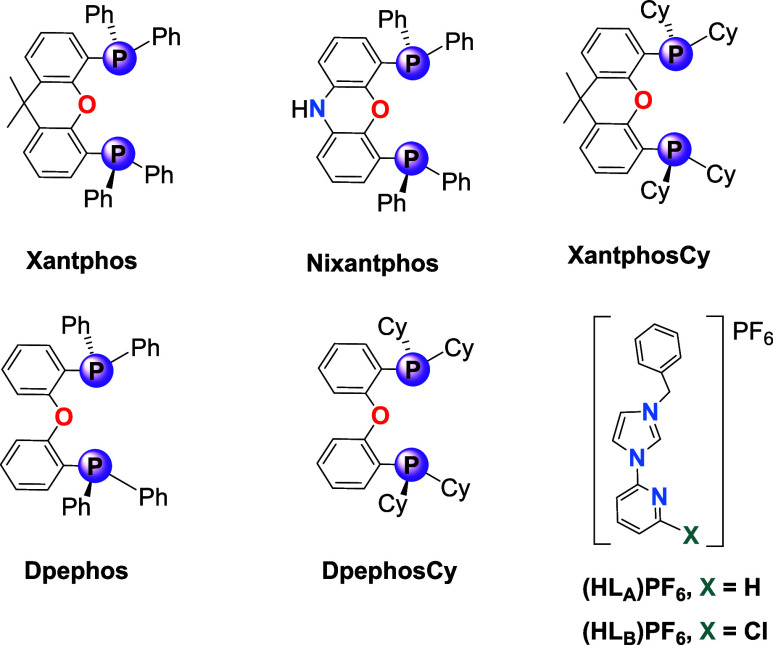
Diphosphanes and NHC ligands selected for this study.

## Discussion

### Synthesis and Characterization

Although transmetalation from silver derivatives has been documented for synthesizing similar compounds, it is rarely employed.^19^ Instead, complexes [Cu­(NHC)­(P^P)]­PF_6_ (**Cu1**-**Cu6)** (Scheme 1) have been synthesized following a reported literature method, involving the reaction of copper metal with the corresponding diphosphane and imidazolium salt [Bz-HIm-2-XPy]­PF_6_ [X = H, **HL**_**A**_ **(PF**_**6**_); Cl, **HL**_**B**_ **(PF**_**6**_)] (Scheme 1).

**Scheme 1:**
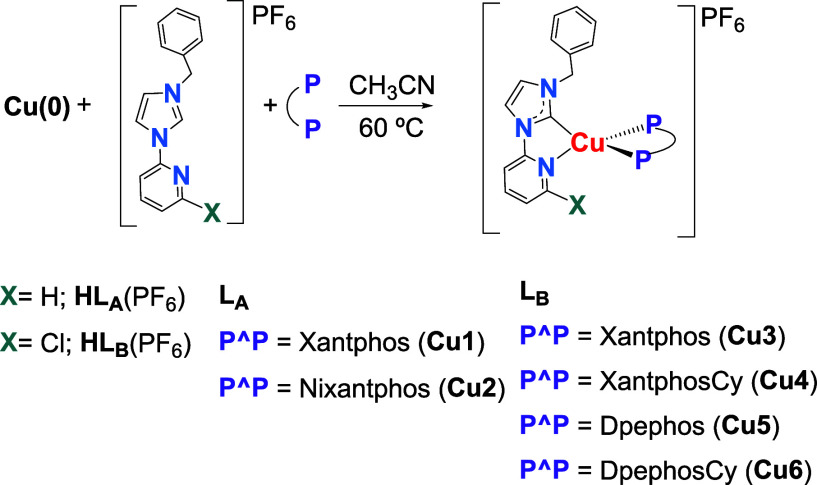
Synthesis of **Cu1**-**Cu6**

The diphosphane excess and reaction times have been adjusted depending on the reagents (see Experimental Part). While the synthesis and photophysical properties of **Cu5** have been previously reported,^23^ no data regarding the analysis of TADF were included. Therefore, compound **Cu5** has been prepared, in order to enable a comparative study of the TADF and other experimental parameters between compounds **Cu1**-**Cu6**.

The expected tetrahedral environment around the copper atom, involving the phosphorus atoms, the carbene carbon, and the pyridine nitrogen atoms of the **L**_**A**_ or **L**_**B**_ ligands (Figures 3,4 and S34) has been confirmed by X-ray diffraction studies for complexes **Cu1**-**Cu3**. The structural data can be compared with those previously reported for **Cu5**.^23^ The angle (α) between the plane defined by the carbene carbon atom, the copper, and the pyridine nitrogen atoms, and the plane defined by the phosphorus and copper atoms informs about the distortion from the ideal tetrahedral geometry (α = 90°). The major distortion is found for **Cu2** (α = 84.00°, Figure 3). Intramolecular π-π interactions are present in these complexes between the centroids of phenyl rings bonded to different phosphorus atoms of the diphosphane.

**Figure 3:**
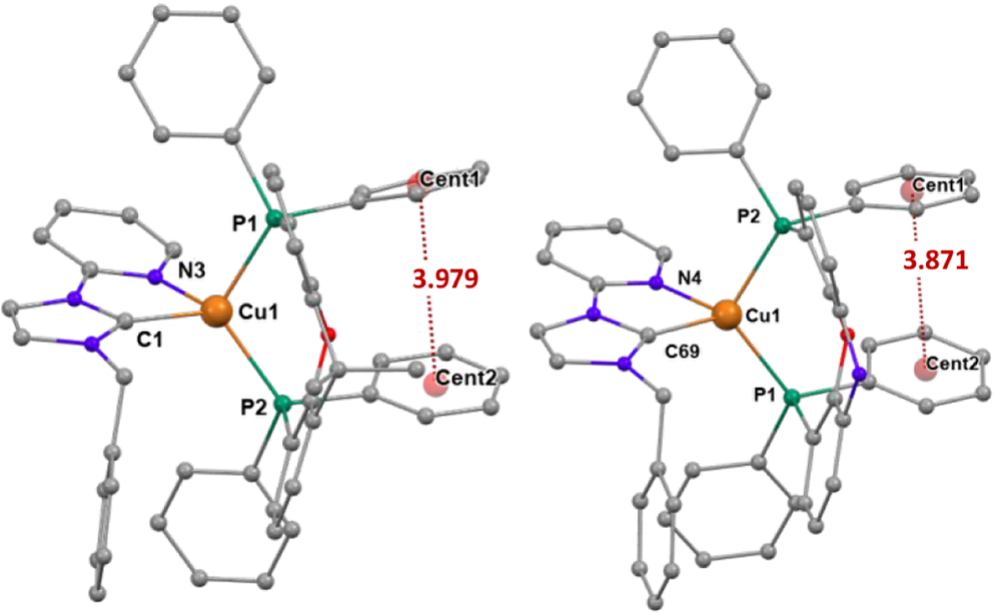
Molecular diagram of the cation of complexes **Cu1** (left) and **Cu2** (right), showing π···π interactions between phenyl rings. Hydrogen atoms have been omitted for clarity. **Cu1**: Bond lengths (Å) P1–Cu1 2.2632(9), P2–Cu1 2.2480(9), N3–Cu1 2.175(3), C1–Cu1 1.966(3). Bond angles (deg) C1–Cu1–N3 79.93(13), P2–Cu1–P1 112.96(4). α= 85.24°. **Cu2**: Bond lengths (Å) P1–Cu1 2.2553(14), P2–Cu1 2.2792(14), N4–Cu1 2.142(5), and C69–Cu1 1.996(6). Bond angles (deg) N4–Cu1–P1 119.90(14), C69–Cu1–P2 116.66(16), C69–Cu1–N4 80.5(2), P1–Cu1–P2 112.34(5). α = 84.00°.

**Figure 4:**
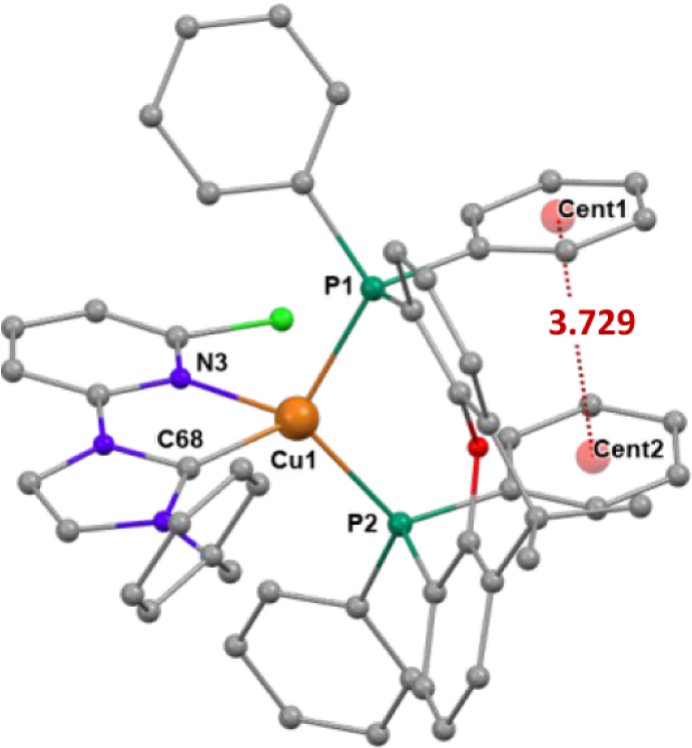
Molecular diagram of the cation of complex **Cu3** showing π···π interactions between the phenyl rings. Hydrogen atoms have been omitted for clarity. Bond lengths (Å) P1–Cu1 2.2825(6), P2–Cu1 2.2640(6), N3–Cu1 2.2412(19), C68–Cu1 1.980(2). Bond angles (deg) C68–Cu1–N3 78.84(8), P2–Cu1–P1 110.44(2). α = 85.18°.

### Thermogravimetric Studies

As discussed in the Introduction, [Cu­(NHC)­(P^P)]^+^ complexes have been reported to exhibit high quantum yields. However, beyond quantum yield, other factors, such as thermal stability, must be considered to assess the suitability of these complexes for different applications. Thus, we performed the thermogravimetric analysis of complexes **Cu1**-**Cu6** (Figures 5 and S35). All of the complexes are stable up to 400–300 °C with complex **Cu1** being the most stable (up to 400 °C) and complex **Cu5** the least stable (up to 300 °C). Similar values have been reported for complexes [Cu­(L_A_)­(Dpephos)]­PF_6_ and [Cu­(Qbim)­(Dpephos)]­PF_6_ (Qbim = 1-benzyl-3-(quinolin-2-yl)­benzimidazol-2-ylidene).^19^ Regarding weight loss, complexes **Cu1** and **Cu2**, with X = H (Figure 5), exhibit an initial weight loss of approximately 60% of the starting mass which fits the diphosphane loss. In contrast, for **Cu3** and **Cu4**, which contain the L_B_ ligand, the initial weight loss of about 20% is consistent with the carbene ligand loss. This may indicate less strongly bonded carbene in complexes bearing the L_B_ ligand. However, complexes **Cu5** and **Cu6** (also with L_B_) do not follow this trend, and their mass loss occurs more gradually, compared with **Cu1**-**Cu4**. Copper Cu-E (E = N, P) bond distances in **Cu1** with L_A_ are marginally shorter than those for complex **Cu3** with L_B_, both containing Xantphos (see ESI).

**Figure 5:**
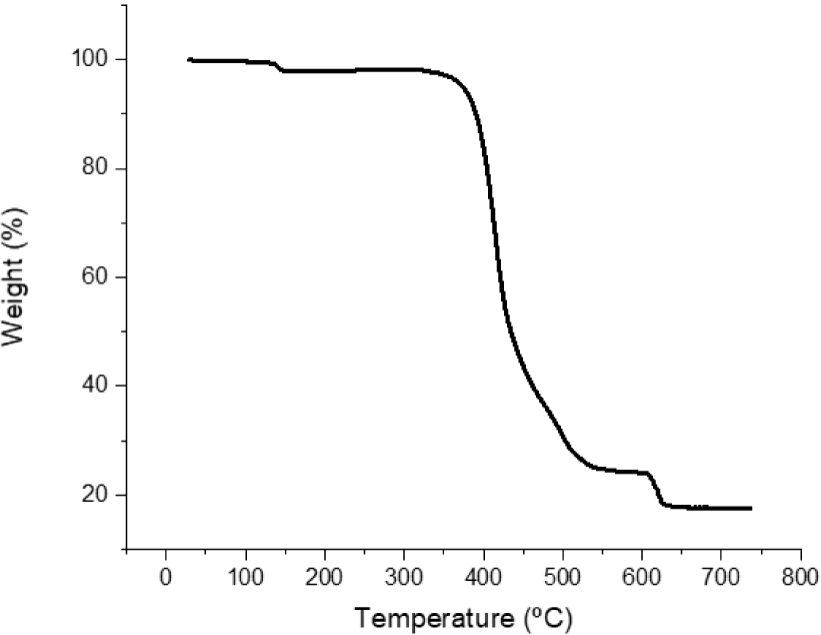
TGA curve of compound **Cu1** as representative of the behavior of **Cu1** and **Cu2**.

In previously reported studies, [Cu­(L_A_)­(Dpephos)]­PF_6_ and [Cu­(Qbim)­(Dpephos)]­PF_6_ ^19^ primarily undergo single-step weight loss, corresponding to about 80% of the initial mass.

To further contextualize these thermogravimetric results, a comparison with reported studies on [Cu­(N^N)­(P^P)]^+^ complexes could be valuable.^26–29^ The relevant conclusion is that with regard to thermal stability, the use of [Cu­(NHC)­(P^P)]^+^ may represent an alternative to the use of [Cu­(N^N)­(P^P)]^+^ as the initial weight loss temperatures are comparable.

### Photophysical Properties

#### Steady-State and Lifetime Studies

Table 1 summarizes the excitation and emission wavelengths and lifetimes of complexes **Cu1**-**Cu6** in the solid state, both at room temperature (rt) and at 77 K (Table 1, Figures S36 and S37), and in PMMA films at room temperature (Figure S38). They are green emitters (Figure 6) and 1931 CIE coordinates have been calculated from the quantum yield studies after excitation light was excluded (Figure S39).

**Figure 6:**
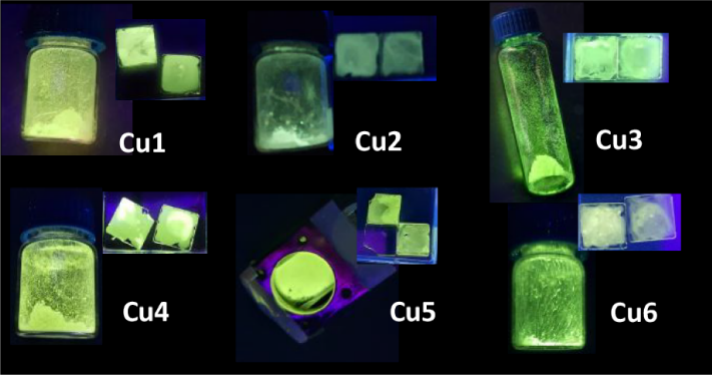
Solid (in vials) and 5 wt % PMMA films (in square quartz sheets) of complexes were studied in this work under UV light (360 nm). For compound **Cu5,** the solid is shown in the cryostat accessory for measuring solid samples.

**Table 1:** Photophysical Data of **Cu1**-**Cu6** and [Cu­(L_A_)­(Dpephos)]­PF_6_ (**I**) in the Solid State (s) and 5 wt% PMMA Film (f)

		λex(nm)	λem(nm)	τ(μs)^d^	Φ (%)	K_r_ ^e^ (μs^–1^)	K_nr_ ^e^ (μs^–1^)
**Cu1**	rt (s)^a^	370	515	76.21	62	8.13 × 10^–3^	4.99 × 10^–3^
	77 K (s)^a^	356	531	140.3			
	rt (f)	336	516	40.07	23	5.73 × 10^–3^	1.92 × 10^–2^
**Cu2**	rt (s)^a^	388	543	61.80	17	2.75 × 10^–3^	1.3410^–2^
	77 K (s)^a^	364	544	137.3			
	rt (f)	332	537	36.99	13	3.51 × 10^–3^	2.35 × 10^–2^
**Cu3**	rt (s)^a^	388	518	44.43	85	1.91 × 10^–2^	3.37 × 10^–2^
	77 K (s)^a^	370	538	135.3			
	rt (f)	350	516	28	23	8.21 × 10^–3^	2.75 × 10^–2^
**Cu4**	rt (s)^a^	380	540	14.09	18	1.27 × 10^–2^	5.81 × 10^–2^
	77 K (s)^a^	380	540	97.27			
	rt (f)	344	532	22	22	1.00 × 10^–2^	4.00 × 10^–2^
**Cu5** ^b^	rt (s)^a^	383	538	48.57	54	1.11 × 10^–2^	9.47 × 10^–3^
	77 K (s)^a^	365	560	159.5			
	rt (f)	288, 346	531	37.91	29	7.64 × 10^–3^	1.87 × 10^–2^
**Cu6**	rt (s)	350	539	38.6	53	1.37 × 10^–2^	1.22 × 10^–2^
	77 K (s)^a^	358	553	79.50			
	rt (f)	286, 340	539	21.41	19	8.87 × 10^–3^	3.78 × 10^–2^
Cu5* ^b^	rt (s)		539	82.4	58	^f^7.04 × 10^–3^	^f^5.09 × 10^–3^
	77 K (s)		563	253.9			
**I** ^c^	rt (s)		520	79.84	56	^f^7.01 × 10^–3^	^f^5.51 × 10^–3^
	77 K (s)		553	160.7			

a Lifetimes at 300 and 80 K, from the study with the cryostat.

b Data for [Cu­(L_B_)­(Dpephos)]­PF_6_: this work (**Cu5**) and reported (**Cu5** ^*^).^23^

c Reported for [Cu­(L_A_)­(Dpephos)]­PF_6_ (**I**).^19^

d Fitted to a two exponential equation (see Supporting Information).

e K_r_ = Φ/τ; K_nr_ = (1-Φ)/τ.

f Calculated from the lifetime and quantum yield reported data.

Lifetimes at room temperature, both of film and solid samples, are on the order of microseconds (Figures S40–S57). Table 1 shows that, in general, the lifetimes of film samples are shorter compared with those of solid samples of the same compound. The different quantum yields of the solid and film samples are illustrated by the K_r_ constants.

Lifetime of powder samples increases upon cooling from room temperature to low temperature. Additionally, as a general trend, a red shift of the emission maximum is observed upon cooling. These trends are consistent with the expected TADF behavior for **Cu1**-**Cu6**.

### TADF Studies

Experimental lifetime of solid samples at different temperatures may be fitted to eq 1, proving the TADF behavior of the complexes. This study is not included among the photophysical data reported for **Cu5**,^23^ so we performed the analysis for comparison.

Experimental data have been fitted to eq 1 using the least-squares fitting method, and values of the first singlet and triplet excited states, as well as the energy gap ΔE­(S_1_-T_1_) have been obtained^30,31^ (K_B_ = Boltzmann constant). A representative example is shown in Figure 7 for compound **Cu3** (see fitting curves for the rest of the complexes in Figures S58 and S59). Due to the temperature working range of the cryostat, in some cases, it was not possible to observe the complete high-temperature plateau (see below). The resulting data summarized in Table 2 show that prompt fluorescence is at least 1 order of magnitude shorter than the TADF lifetime, and ΔE­(S_1_-T_1_) energy gaps are below 1000 cm^–1^, except for **Cu6**. Prompt fluorescence may be expected at lower lifetimes,^32^ but several nanoseconds fluorescence has been reported, for instance, 180 ns for other tetracoordinated copper complexes.^31^ For the complexes in this work, the longer and shorter prompt fluorescence values are 0.460 μs (**Cu5**) and 0.080 μs (**Cu4**) respectively. These values are very different from the shorter [8.6 μs (**Cu4**)] and longer [53.48 μs (**Cu5**)] TADF lifetime components observed in ESI.

1 $$\tau =\frac{3+e^{{‐\Delta E(S}_{1}{‐T}_{1}{\mathrm{)/K}}_{B}T}}{3\left(\frac{1}{{\mathrm{\tau (T}}_{1})}\right)+\left(\frac{1}{{\mathrm{\tau (S}}_{1})}\right)e^{{‐\Delta E(S}_{1}{‐T}_{1}{\mathrm{)/K}}_{B}T}}$$

**Figure 7:**
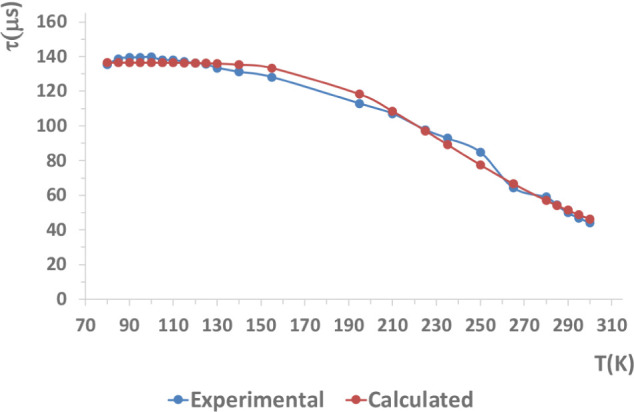
Temperature dependence of the emission lifetime of complex **Cu3** in the solid state and the corresponding fitting values using eq 1 (Chi-Square 0.993).

**Table 2:** Delayed Fluorescence Lifetime and Lifetimes of Excited States S_1_ and T_1_ (μs) and ΔE­(S_1_-T_1_) Energy Gap (cm^–1^) from Fitting Lifetime Data at Different Temperatures to eq 1

Compound	τ(S_1_)	τ(T_1_)	ΔE(S_1_-T_1_)
**Cu1**	1.492	134.84	773.73
**Cu2**	1.045	131.99	774.01
**Cu3**	0.206	136.42	984.67
**Cu4**	0.080	94.823	948.38
**Cu5**	0.381	152.15	887.75

In general, a smaller increment in the lifetime upon cooling is observed for complexes [Cu­(NHC)­(P^P)]^+^, compared with most of the [Cu­(N^N)­(P^P)]^+^ reported compounds displaying TADF. This has been attributed to a double emissive origin, based on phosphorescence and TADF.^23^ Representation of lifetimes vs temperature for TADF complexes would display three regions, at low temperature, a plateau is expected, corresponding to phosphorescence, at high temperature a plateau is expected, corresponding to TADF; and the intermediate region combines phosphorescence and TADF. As commented above, no plateau is observed at room temperature, indicating the contribution of both phosphorescence and TADF at room temperature.^30^ In this sense, the fitting for complex **Cu6** lacks good quality, the first plateau stops at near 270 K, and the calculated ΔE­(S_1_-T_1_) energy gap is higher than 2000 cm^–1^ pointing to a phosphorescent decay.

### Quantum Yield Analysis

The general quantum yields of complexes **Cu1**-**Cu6** and **I** range from 13 to 85%. However, it is important to note that PMMA film samples exhibit similar quantum yields (around 20%), despite significant structural variations, such as substitutions at the phosphorus atom, modifications to the diphosphane skeleton, or the presence of a chloride on the pyridine ring. In contrast, solid samples show much more diverse quantum yield values. In general, films show a lower *k*_r_ than the corresponding solid samples, which fit with the observed quantum yields. In addition to the molecular steric hindrance, which is expected to suppress nonradiative deactivation, media rigidity must also be considered. Both increases and decreases in quantum yield have been reported when comparing solid and PMMA film samples, yet a general explanation remains elusive. While increased medium rigidity is generally expected to enhance quantum yield, the influence of specific interactions between the compound and the embedding medium (PMMA, in this case) is complex and difficult to quantify. These interactions may account for the divergent trends observed across different complexes when comparing measurements in the solid state versus PMMA films.^31,33–35^

### Solid-State Quantum Yields

We have focused on solid-state data and analyzed different factors, looking for trends governing the final quantum yield: structural ligand changes (as the diphosphane skeleton or substituents at phosphorus) and global molecular factors (energetic or steric factors involving the hole molecule). In addition to the expected deactivation pathways associated with molecular flexibility, tetracoordinated copper complexes may undergo distortions from their ideal tetrahedral geometry, giving rise to square-planar-like excited states that facilitate nonradiative deactivation.

#### Structural Ligand Changes Influence

*Presence of the chloride atom in the pyridine ring.* As the quantum yield of **Cu3** is higher than that of **Cu1** (both with the Xantphos diphosphane), it could be deduced that the presence of the chloride in the pyridine ring leads to an important increment in the quantum yield. However, such an effect is not observed for complexes with the Dpephos diphosphane, for which no noticeable difference is observed when the chloride atom at the pyridine ring is present (see Table 4, **I** and **Cu5**). Therefore, the presence of a chloride atom in the pyridine ring does not lead to an increase in the quantum yield for any diphosphane.

#### Structural Ligand Changes Influence

*Diphosphane skeleton*. In the solid state, the diphosphane backbone seems to play a relevant role in the quantum yield (Tables 1 and 4). Substituting the diphosphane Dpephos, mostly used in the reported [Cu­(NHC)­(P^P)]­PF_6_ complexes, by Xantphos, with either L_A_ or L_B_ ligands, leads to higher quantum yields. Specifically, complexes with the Xantphos diphosphane (**Cu1**, **Cu3**) display a higher quantum yield than **Cu5**, whereas the use of Nixantphos leads to lower quantum yields.

#### Structural Ligand Changes Influence

*Substituents at the phosphorus atom.* Change of phenyl by cyclohexyl substituents at the phosphorus atoms bonded to the 9,9-dimethylxanthene backbone (compounds **Cu3** and **Cu4)** leads to a significant decrease in the quantum yield. However, when the same substitution is made at the phosphorus atoms in complexes with the bis­(phenyl)­ether backbone (compounds **I** and **Cu6**), no significant change in quantum yield is observed.

Thus, the presence of a chloride substituent on the pyridine ring and diphosphane substituents at phosphorus results in different outcomes, depending on the diphosphane backbone. These findings suggest that rather than focusing on specific structural elements, a more comprehensive analysis of the global energetic and steric factors of the molecule may be necessary to understand the observed results.

#### Global Molecular Factors Influence

*ΔE­(S*_1_ *-T*_1_ *) energy gap.* A small ΔE­(S_1_-T_1_) energy gap is required for TADF to occur, but Table 3 shows that the smallest energy gap does not guarantee the highest quantum yield. For compound **Cu6,** data indicate that a significant quantum yield is achieved via a phosphorescent mechanism.

**Table 3:**
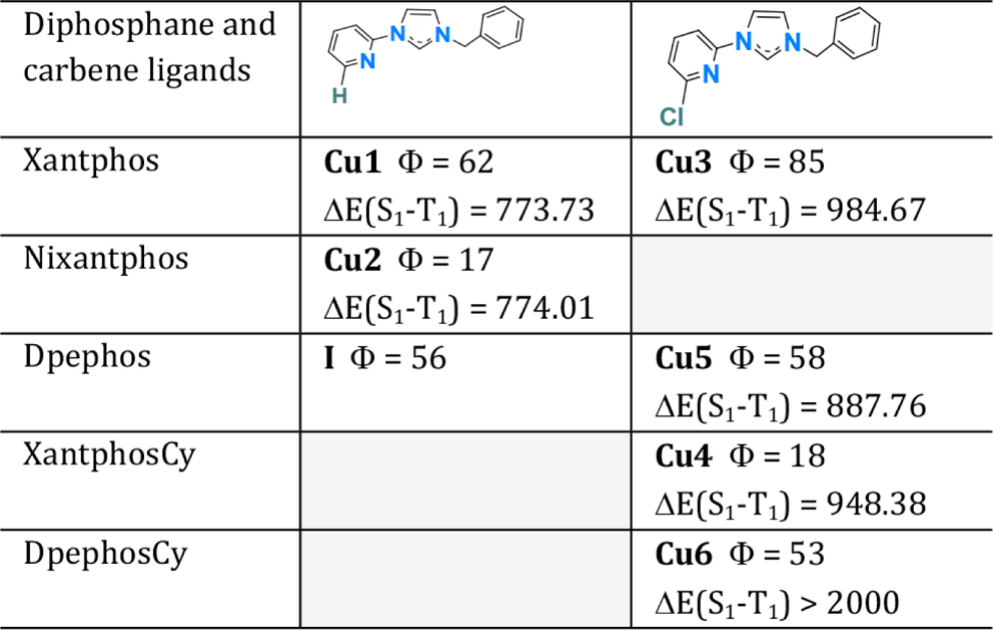
Overview of Quantum Yields (%) in the Solid State and ΔE­(S_1_-T_1_) Energy Gaps (cm^–1^)

#### Global Molecular Factors Influence

*Other.* Reported theoretical results for different complexes [Cu­(NHC)­(P^P)]­PF_6_ complexes reveal that the copper atom and diphosphane mostly contribute to the HOMO orbital, whereas those of the NHC ligand mostly contribute to the LUMO orbital,^13,16–18,20,23^ although it also may contribute to a greater or lesser extent to the HOMO orbital.^14,19^ Thus, in general, transitions are attributed to metal ligand (copper, L = P^P) to ligand (L’= NHC) (MLL’CT) mixed with intraligand (L’= NHC) IL’CT charge transfer transitions. The analysis of these data, in terms of the contributions of copper and both ligands to the frontier orbitals, has proven useful in some cases to rationalize the shifts in emission maxima following modification of the donor or electron-withdrawing nature of the substituents on the carbene core or wings of the NHC ligand. However, no conclusions are provided for the observed changes in the quantum yield, as the nature of the transition does not provide information about the influence of nonradiative decay channels.

Other factors have been analyzed in order to rationalize the quantum yield in heteroleptic [Cu­(N^N)­(P^P)]^+^ complexes, as (i) the buried volume percentage (%V_bur_) (the occupied volume by a given ligand or group of ligands inside a sphere of a defined radius around the metal center), which may inform about the influence of steric factors in structural distortion, and (ii) the excited triplet state energy.^36^

The atomic coordinates generated from the X-ray data have been used to calculate the %V_bur_ of the diphosphane [P^P %V_bur_], that of the carbene ligand [CHN %V_bur_] and the %V_bur_ corresponding to both ligands together [total %V_bur_] for each compound and are shown in Table 4 and illustrated for **Cu1** in Figure 8 and for the rest of the complexes in Figures S60–S64. For comparison, we have also calculated the corresponding values for the already known complexes [Cu­(L_A_)­(Dpephos)]^+^ (**I**) and [Cu­(L_B_)­(Dpephos)]^+^ (**Cu5**), which had not been reported.

**Figure 8:**
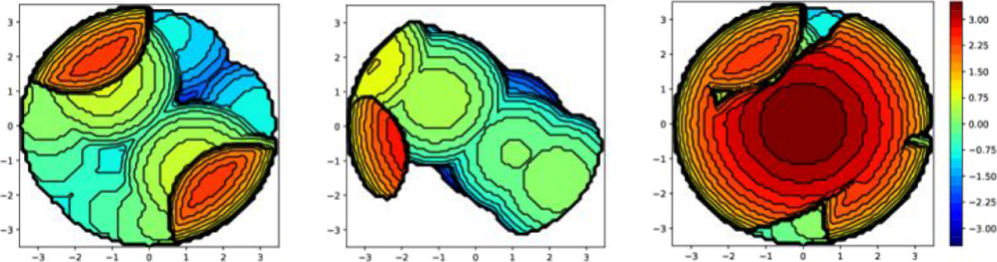
Topographical steric maps for Xantphos, **L**_**A**_ and {(Xantphos)+(**L**_**A**_)} in compound [Cu­(Xantphos)­(**L**_**A**_)]­PF_6_ (**Cu1**).

The highest total %V_bur_ values are found for compound **Cu5** with the **L**_**A**_ ligand and Dpephos. The Dpephos %V_bur_ is always bigger than that corresponding to the more rigid diphosphanes Xantphos, Nixantphos, or XanphosCy and the %V_bur_ of **L**_**A**_ is always smaller than that of **L**_**B**._ Thus, the higher flexibility of the diphosphane Dpephos seems to lead to higher [P^P %V_bur_] and the presence of the chloride atom in the pyridine ring leads to higher [C^N %V_bur_].

The distortion from the tetrahedral to the square planar geometry may become more difficult as the steric demand increases [which may be estimated from %V_bur_)] and the deactivation from the square planar excited states is expected to be avoided for higher %V_bur._ ^12,36^ The highest total %V_bur_ is found for **Cu5**, which does not display the highest quantum yield, so this unique factor does not explain the observed value.

The calculated energies of the first excited triplet are shown in Table 4. High energy values can hinder deactivation and lead to higher quantum yields.^18^ For the analyzed complexes, the highest T_1_ energy is found for **Cu3**. Thus, for compound **Cu3**, which displays high total buried volume and the highest T_1_ energy, these two factors combinate to result in the highest quantum yield. It appears that a high quantum yield is achieved through a balance between these two factors. Other factors, such as the organization of the molecules in the pristine solids, could likewise affect the quantum yield.

**Table 4:** %V_bur_ ^a^ for P^P, C^N and {(P^P)+(NHC)} Blocks in Complexes **Cu1–Cu5** and **I**

Compound	P^P	NHC	{(P^P)+(NHC)}	Φ(%)^b^	E(T_1_) (cm^–1^)^e^
**Cu1**	56.4	36.0	90.8	62	21.4 × 10^3^
**Cu2**	56.1	37.9	90.3	17	21.6 × 10^3^
**Cu3**	55.7	37.1	91.4	85	21.8 × 10^3^
**Cu4**				18	20.6 × 10^3^
**I**	57.3^d^	35.3^d^	90.4^d^	56^c^	
**Cu5**	57.4^d^	38.3^d^	92.7^d^	58^c^/54	20.4 × 10^3^

a Calculated from the crystal structure data, see Supporting Information for additional details.

b Obtained for powder samples, at room temperature.

c Reported data for **I** ^19^ and **Cu5**.^23^

d Calculated from the coordinates corresponding to the reported crystal structure of **I** ^19^ and **Cu5**.^23^

e Calculated from the λ_onset_ of the emission spectrum at 77 K.^37^

### Electrochemistry

HOMO and LUMO energies may help to evaluate the usefulness of the complexes for different applications. These values may be estimated from the cyclic voltammetry curves, which are illustrated in Figure 9 and more data are shown in Figures S65 and S66. The complexes do not show reversible processes. Based on studies on tetracoordinated complexes with diphosphanes as ligands, oxidation at higher potential may be attributed to copper Cu­(I)/Cu­(II) oxidation, while the oxidation peak beyond this peak is proposed to correspond to diphosphane oxidation. The reduction peak at lower potential is proposed to correspond to processes involving the NHC ligand.^38^ This peak is much more poorly defined than the oxidation peak. This assignation fits with the theoretical results discussed above in which copper and diphosphane contribute mostly to the HOMO orbital and the carbene to the LUMO orbital.^13,14,16–18,20^

**Figure 9:**
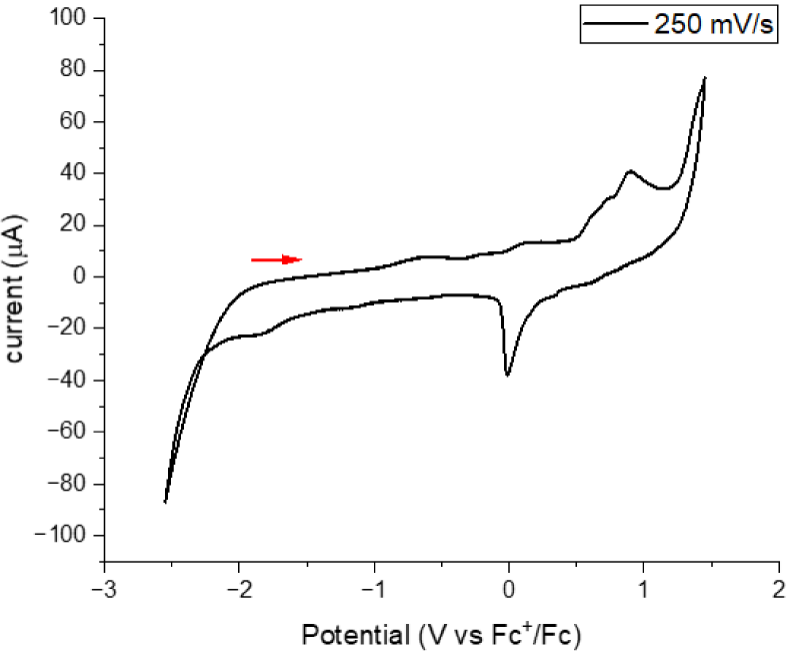
Cyclic voltammogram of **Cu1** in CH_2_Cl_2_ with ^n^Bu_4_PF_6_ as supporting electrolyte, values are referenced to Fc/Fc^+^ recorded at 250 mV/s.

It must be taken into account that these calculations are merely indicative, which is reinforced by the poor definition of the reduction peaks in many of the complexes. We selected **Cu3** and **Cu6** to study using a scan from −0.5 V to −2.4 V, but no reduction peak was detected. Although the values should be regarded as tentative, they have been included, as they may still serve as a useful guide for potential applications. The HOMO and LUMO calculated values are shown in Table 5 and fall within the rage of those reported for other [Cu­(NHC)­(P^P)]^+^ complexes, obtained through theoretical calculations. However, HOMO energies are slightly lower, resulting in HOMO–LUMO gaps that resemble those reported for complex **Cu5** and analogous complexes with different substituents at the pyridine ring (Figure 10a).^23^ These energy gaps are smaller than many previously reported, which in some cases are near 5 eV.^13,14,16–18^ Notably, complexes with chloride or methyl substituents on the imidazole ring of L_A_ exhibit some of the highest energy values (HOMO ≈ 8 eV, LUMO ≈ 4 eV) (Figure 10b).^14^

**Table 5:** Data from the Cyclic Voltammetry Studies (eV)^a^

Compound	E_HOMO_ ^b^	E_LUMO_ ^b^	ΔE
**Cu1**	–5.39	–2.99	2.40
**Cu2**	–5.37	–3.21	2.16
**Cu3**	–5.50	–^c^	
**Cu4**	–5.70	–2.9	2.80
**Cu5**	–5.51	–3.23	2.28
**Cu6**	–5.62	–^c^	

a From the graph recorded at recorded at 250 mV/s in CH_2_Cl_2_ with ^n^Bu_4_PF_6_ as supporting electrolyte. Values are referenced to Fc/Fc^+^.

b LUMO = −(E_onset red_ + 4.8), HOMO = −(E_onset ox_ + 4.8).^39–41^

c Not clear peak observed.

**Figure 10:**
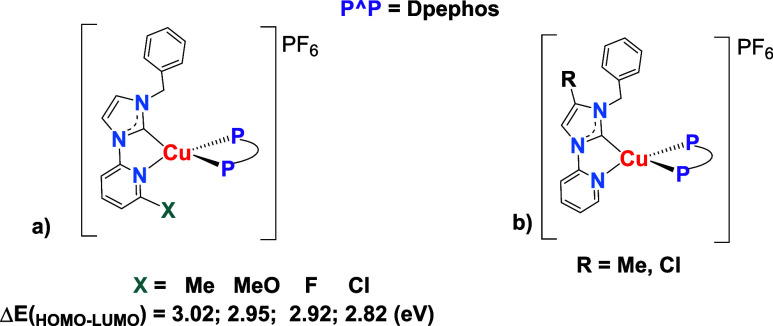
a) ΔE­(_HOMO–LUMO_) energy gaps reported for complexes with different substituents bonded to the pyridine ring, including **Cu5**.^23^ b) Complexes with different substituents bonded to the imidazole ring of L_A._ ^14^.

## Conclusions

Some of the new complexes here reported exhibit quantum yields higher than those of similar complexes with the same carbene ligands. Notably, **Cu3** reaches a quantum yield of 85% in the solid state, which is lower than the 96% reported for [Cu­{Im-2-Mepy}­(Dpephos)]­PF_6_ (61% as crystal powder) but higher than the values reported for other complexes of the [Cu­{Im-Z-Mepy}­(Dpephos)]­PF_6_ family in the solid state^14,23^ or in film.^13,16^ These results reveal that high quantum yields can be achieved by modifying the diphosphane with simple carbene skeletons and wings, eliminating the need for complex NHC structures.

The analysis of the previously unexplored influence of the diphosphane on the emissive properties of heteroleptic [Cu­(NHC)­(P^P)]­PF_6_ indicates that, except for **Cu6**, the studied complexes are TADF emitters in the solid state with a mixed TADF-phosphorescent character. Changing the diphosphane modifies the ΔE­(S_1_-T_1_) energy gap. Data for compound **Cu6** point out a phosphorescent nature.

Changing the diphosphane does not result in a noticeable modification of the quantum yields for PMMA films. Regarding the quantum yield in the solid state, the structural backbone of the diphosphane appears to have the most significant impact, with quantum yield increasing in the order: Xantphos > Dpephos > Nixantphos. The presence of a chloride substituent on the pyridine ring and diphosphane substituents at phosphorus results in different outcomes, depending on the diphosphane backbone.

Furthermore, while high buried volume percentage and high T_1_ energy both seem to lead to high quantum yield, they may offset one another, highlighting the complex interplay between these factors in optimizing the quantum yield.

## Experimental Section

### Instrumentation

NMR spectra (Figures S1–S28) were carried out in a Bruker AV 400 or 300. Chemical shifts (ppm) are reported relative to the solvent peaks of the deuterated solvent.^42^ A Bruker MicroTOF-Q spectrometer was used for high-resolution mass spectra-ESI (HRMS-ESI) equipped with an API-ESI source and a QTOFmass analyzer, both allowing a maximum error in the measurement of 5 ppm. Mass spectra are shown in Figures S29–S33). The termogravimetric analysis was carried out in a TA Instruments SDT2960 equipment at a rate of 10 °C min^–1^ under a nitrogen atmosphere until 600 °C and under an air atmosphere from 600 to 750 °C and the corresponding TGA curves are shown in Figure S35.

Steady-state photoluminescence spectra (Figures S36 and S38) and lifetimes (Figures S40 and S57) were recorded using a FluoTime300 PicoQuant spectrometer. Solid powders were placed within a quartz tube and placed inside a quartz dewar, films were placed in an adjustable front-face holder. An OptistatDN Oxford variable temperature liquid nitrogen cryostat has been used for lifetime studies at different temperatures. Quantum yields were measured by the absolute method using a Hamamatsu Quantaurus-QY C11347 compact one-box absolute quantum yield measurement system. In order to prove the reproducibility of the measurements, three or more measurements were carried out for each compound with different amount of solid; for films, two or three reference films and the same number of samples were prepared, and each sample was measured with each reference. For film preparation, a solution of ca. 4 mg of compound and ca. 76 mg of PMMA in 1 mL of CH_2_Cl_2_, was sonicated for 15–20 min. Films were prepared by drop casting the resulting solution. Reference film samples were prepared as explained below but without adding the copper compound. In order to prove reproducibility of the measurements of solid samples, different amounts of each compound in a quartz tube were measured, and for films, two or three reference samples and two or three films of each compound were prepared, with each sample measured against all the references. Through studies carried out for different substances using both, the absolute method and the comparative method, the relative uncertainty for the absolute method has been determined as less than 6%.^43^

**Electrochemical experiments** of complexes **Cu1**-**Cu5** in dichloromethane solutions of about 5 × 10^–4^ M were performed using a potenciostat/galvanostat EG&G Research Model 273 in a glass cell using three electrodes: Ag/AgCl (3 M) reference electrode, platinum wire auxiliary electrode, and a glassy carbon disk working electrode. In order to ensure the absence of electroactive impurities, a 0.1 M NBu_4_PF_6_ supporting solution was scanned over the solvent (CH_2_Cl_2_) window. A total of five cycles were acquired for each respective scan rate. Cyclic voltamograms are shown in Figures S65 and S66.

### Crystallography

Crystals suitable for X-ray studies were obtained by diffusion of diethyl ether into acetonitrile or acetone solutions of the complexes. Crystals were mounted on a MiTeGen Crystal micromount and transferred to the cold gas stream of a Bruker D8 VENTURE (2) diffractometer. Data were collected using monochromated MoKα radiation (λ = 0.71073 Å). Scan type ω. Absorption corrections based on multiple scans were applied with the program SADABS.^44^ The structures were refined on F^2^ using the program SHELXL-2018.^45^ All non-hydrogen atoms were refined anisotropically. Hydrogen atoms were included by using a riding model. CCDC depositions 2430838 (**Cu1**), 2430837 (**Cu2**) and 2430839 (**Cu3**) contain the supplementary crystallographic data. These data can be obtained free of charge from the Cambridge Crystallography Data Center. ORTEP diagrams of the cation of complexes **Cu1**-**Cu3** are shown in Figure S34.

### Calculation of the Buried Volume

SambVca 2.1 program^46^ https://www.molnac.unisa.it/OMtools/sambvca2.1/idex.html) was used to calculate buried volumes^47^ and topographical steric maps (Figures S60–S64).^48^ The center of the sphere was defined by the copper atom, and the selected parameters were: Bondi radii: 1.17; sphere radius *r* = 3.5 Å; mesh spacing 0.10, and the H atoms excluded. Selection of axis and atoms used for each %V_bur_: *for diphosphane [%V*_*bur*_ *(P^P)]* the P atoms of the diphosphane were selected to define the negative *Z*-axis and the C and N atoms of the carbene ligand coordinated to the copper center of the carbene ligand to define the XZ-plane. All atoms except those of the diphosphane ligand were deleted; *for carbene [%V*_*bur*_ *(NHC)]:* the C and N atoms of the carbene ligand coordinated to the cooper center were used to define the negative *Z*-axis and the P atoms of the diphosphane to define the XZ-plane. All the atoms except those of the carbene ligand were deleted; *for diphosphane + carbene: [%V*_*bur*_ *{P^P) + (NHC)}]* the C and N atoms of the carbene ligand coordinated to the copper center were used to define the negative *Z*-axis and the P atoms of the diphosphane to define the XZ-plane. The Cu atom was deleted.

### Synthesis of Complexes [Cu­(NHC)­(P^P)]­PF_6_

#### General Procedures

The synthetic procedures were carried out under an Ar atmosphere, using Schlenk techniques with degassed solvents. The starting materials Cu, Xantphos, XantphosCy, Dpephos, DpephosCy, and Nixantphos, are commercially available and were used as received. The imidazolium salts (**HL**_**A**_ **)­PF**_**6**_ and (**HL**_**B**_ **)­PF**_**6**_ have been synthesized from the **Im-2-Zpy** precursors (Z = H, Cl) following reported methods.^19,23^

#### Synthesis of Cu1–Cu6

Synthesis of the copper complexes has been carried out from copper powder, the diphosphane, and the corresponding **L**_**X**_ ligand (X = A, B), by adapting the excess of copper, diphosphane, and/or reaction time. As the synthesis of **Cu5** has been previously reported^23^ no characterization data are given.

The synthetic procedure consists of mixing copper powder, the corresponding NHC ligand, and disphosphane in degassed acetonitrile as the solvent. The mixture is heated to 50–60 °C and stirred^13–18,21–24^ for a general time of 14 h (X = H) or 24–48 h (X = Cl, **Cu3** = 24 h, **Cu4** = 24 h, **Cu5** = 48 h, **Cu6** = 48 h) The reaction mixture is allowed to cool and filtrated through Celite. The filtrate is concentrated (∼1 mL), and by the addition of diethyl ether (∼8 mL), a yellow solid is obtained, which is washed with Et_2_O.

The quantities listed below (in 15 mL of CH_3_CN) were employed:

Carbene: 0.2 mmol (**HL**_**A**_ = 76.22 mg, **HL**_**B**_ = 83.96 mg). Copper: 0.4 mmol of Cu^0^ (25.41 mg). Diphosphane: 0.24 mmol [P^P = Xantphos (**Cu1** and **Cu3**, 138.87 mg); XantphosCy (**Cu4**, 135.06 mg)]; 0,4 mmol [Nixantphos (**Cu2**, 132.37 mg); Dpephos (**Cu5**, 215.42); DpephosCy (**Cu6**, 225 mg)].

The ^31^P­{^1^H} NMR spectra of the complexes display one broad signal at ca. −10 ppm. In the ^1^H NMR spectra the signal corresponding to the CH_2_ unit of the benzyl group appears at about 5 ppm and those arising from the cyclohexyl substituents as broad signals in **Cu4** and **Cu6** between 0 and 3 ppm. The benzyl methylene carbon appears near 56 ppm in the ^13^C NMR spectra and those of the methyl fragments of Xantphos and XantphosCy at about 30 ppm, and the quaternary carbon bonded to these methyl groups at about 40 ppm. Despite the overlap of many signals, both in the ^1^H and ^13^C NMR spectra, some of the carbon and hydrogen atoms have been assigned through bidirectional NMR experiments (ESI).

**Cu1:** Yield = (132.6 mg, 65%). ^**1**^ **H NMR** (400 MHz, acetone-d_6_) δ (ppm): 8.30 (d, *J* = 2.0 Hz, 1H, **c**), 8.12–8.01 (m, 3H), 7.73 (dd, *J* = 7.8 Hz; 1.4 Hz, 2H, **f**), 7.46 (d, *J* = 2.0 Hz, 1H, **b**), 7.40–7.08 (20 H), 6.98 (m, 6H), 6.67 (d, *J* = 7.6 Hz, 2H, **d**), 6.55 (m, 2H, **g**), 4.78 (s, 2H, **a**), 1.72 (s, 3H, **e**), 1.66 (s, 3H, **e**). ^**31**^ **P­{** ^**1**^ **H} NMR** (162 MHz, acetone-d_6_) δ (ppm): −9.1. ^**13**^ **C**{^**1**^ **H} NMR** (101 MHz, acetone-d_6_) δ (ppm): 155.74 (CR_4_, C–P), 151.24 (CR_4_), 149.46, 141.62, 136.71 (**h**), 134.92­(**j**), 133.92­(*“t”*, *J* = 7.9 Hz), 137.72 (*“t”*, *J* = 7.9 Hz), 133.40 (CR_4_, C–P), 132.56 (CR_4_, C–P), 131.59 (**g**), 130.80, 129.70, 129.54, 129.50, 128.62 (**f**), 128.51 (**f**), 127.76 (**d**), 126.11, 124.54, 123.85 (**b**), 121.73 (CR_4_, C–P), 118.33 (**c**), 113.43, 55.28 (**a**), 36.87 (**i**), 28.57 (**e**), 27.55 (**e**). **ESI-QTOF (+)** *m*/*z* = [M^+^] calculated for C_45_H_45_CuN_3_OP_2_ 876.2328; found 876.2353.

**Cu2:** Yield = (110.4 mg, 55%). ^**1**^ **H NMR** (400 MHz, acetone-d_6_) δ (ppm): 8.33 (d, *J* = 4.9 Hz, 1H), 8.27 (d, *J* = 2.1 Hz, 1H, **b**), 8.12–8.08 (m, 2H); 8.01 (d, *J* = 8.3 Hz, 1H), 7.51 (d, *J* = 2.1 Hz, 1H, **c**), 7.40 (t, *J* = 7.4 Hz, 2H), 7.33–7.25 (m, 7H), 7.17–7.09 (m, 9H), 7.05–6.97 (10H), 6.84 (dd, *J* = 8.0; 0.8 Hz, 2H, **d**), 6.22–6.17 (m, 2H), 5.09 (s, 2H, **a**). ^**31**^ **P­{** ^**1**^ **H} NMR** (162 MHz, acetone-d_6_) δ (ppm): −10.3. ^**13**^ **C**{^**1**^ **H} NMR** (101 MHz, acetone-d_6_) δ (ppm): 151.21 (CR_4_), 149.56 (**e**), 146.65­(CR_4_), 141.65, 136.42 (CR_4_, C–P), 133.87, 132.28 (CR_4_, C–P), 130.79, 129.64, 128.73 (**d**), 127.98 (**d**), 126.33, 125.21, 124.54, 123.96 (**c**), 121.84 (CR_4_, **f**), 118.25 (**b**), 117.13, 113.41, 55.59 (**a)**. **ESI-QTOF (+)** *m*/*z* = [M^+^] calculated for C_51_H_40_CuN_4_OP_2_ 849.1995; found 849.1967.

**Cu3:** Yield = (109.5 mg, 52%). ^**1**^ **H NMR** (400 MHz, acetone-d_6_) δ (ppm): 8.20 (d, *J* = 2.1 Hz, 1H), 8.15 (*“t”*, *J* = 8.0 Hz, 1H, **e**), 7.94 (d, *J* = 8.0 Hz, 1H, **d**), 7.68 (dd, *J* = 7.8 Hz, 1.3 Hz, 2H), 7.44–7.32 (m, 5H), 7.30 (d, *J* = 2 Hz, 1H), **c**), 7.26 – 7.08 (m, 20H), 7.04 (m, 2H), 6.74 (m, 2H), 6.63 (d, *J* = 7.5 Hz, 2H), 4.80 (s, 2H, **a**), 1.69 (s, 3H, **f**), 1.58 (s, 3H, **f**). ^**31**^ **P­{** ^**1**^ **H} NMR** (162 MHz, acetone-d_6_) δ (ppm): −9.5. ^**13**^ **C**{^**1**^ **H} NMR** (101 MHz, acetone-d_6_) δ (ppm): 155.90 (CR_4_, C–P), 151.17­(CR_4_), 150.60 (CR_4_), 144.16 (**e**), 136.33 (CR_4_), 134.56 (CR_4_, C–P), 133.95 (*“t”*), 133.88 (*“t”*), 133.22 (*“t”*), 132.69 (*“t”*), 131.38, 130.93, 130.80, 129.64 (*“t”*), 129.56 (*“t”*), 128.71, 128.33, 127.56, 125.59, 124.53 (**c**), 123.80, 122.28 (CR_4_), 122.12 (CR_4_), 118.80 (**b**), 111.92 (**d**), 66.10, 54.95 (**a**), 36.74 (CR_4_, C–P). **ESI-QTOF (+)** *m*/*z* = [M^+^] calculated for C_59_H_44_ClCuN_3_OP_2_ 910.1900; found 910.1939.

**Cu4:** Yield (125.0, mg 58%). ^**1**^ **H NMR** (400 MHz, acetone-d_6_) δ (ppm): 8.33 (d, *J* = 2.1 Hz, 1H, **b**), 8.21 (*“t”*, *J* = 8.0 Hz, 1H, **e**), 8.10 (d, *J* = 7.9 Hz, 1H, **d**), 7.64 (dd, *J* = 7.7, 1.3 Hz, 2H), 7.51 (d, *J* = 2.0 Hz, 1H, **c**), 7.46 (d, *J* = 7.7 Hz, 1H, **f**), 7.43–7.39 (m, 2H), 7.31–7.28 (m, 5H), 6.92 (m, 2H), 4.99 (s, 2H, **a**), 1.67 (s, 3H, **g**), 1.49 (s, 3H, **g**), 2.3–0.5 (br, 40H, Cy). ^**31**^ **P­{** ^**1**^ **H} NMR** (162 MHz, acetone-d_6_) δ (ppm): −11.5. ^13^ **C**{^**1**^ **H} NMR** (101 MHz, acetone-d_6_) δ (ppm): 155.56 (CR_4_, C–P), 151.28, 151.21, 144.18 (**e**), 136.48, 133.78 (CR_4_, C–P), 131.26, 129.59, 128.83, 128.00, 128.77, 125.00, 124.74, 123.85 (**f**), 119.66 (d, *J* = 8.62 Hz, CR_4_), 119.56 (d, *J* = 8.62 Hz, CR_4_), 118.40 (**b**), 111.93 (**d**), 55.37 (**a**), 36.25, 34.61­(*“t”*, *J* = 7.51 Hz, Cy), 34.19 (*“t”*, *J* = 7.09 Hz, Cy), 29.04–28.83 (m, Cy/Me), 27.90–27.34 (m, Cy/Me), 27.04 (d, *J* = 10.11 Hz, Cy). **ESI-QTOF (+)** *m*/*z* = [M^+^] calculated for C_54_H_68_ClCuN_3_OP_2_ 934.3802; found 934.3817.

**Cu6:** Yield (83.49 mg, 40%). ^**1**^ **H NMR** (300 MHz, acetone-d_6_) δ (ppm): 8.27 (d, *J* = 2.1 Hz, 1H, **b**), 8.21 (*“t”*, *J* = 8.0 Hz, 1H, **e**), 8.04 (d, *J* = 8.0 Hz, 1H, **d**), 7.65 (m, 3H), 7.55 (m, 2H), 7.51 (d, *J* = 2.1 Hz, 1H, **c**), 7.38–7.30 (m, 7H), 7.05 – 7.02 (m, 2H, **f**), 5.38 (s, 2H, **a**), 2.16–0.66 (m, 40H, Cy). ^**31**^ **P­{** ^**1**^ **H} NMR** (162 MHz, acetone-d_6_) δ (ppm): −8.9. ^13^ **C**{^**1**^ **H} NMR** (101 MHz, acetone-d_6_) δ (ppm): 159.77 (*“t”*, *J* = 5.34 Hz, CR_4_, C–P), 151.90, 151.17, 143.98 (**e**), 136.38, 134.65, 132.10, 129.63, 128.87, 128.01, 125.10 (*“t”*), 124.54, 124.22 (**c**), 122.50 (d, *J* = 8.79 Hz), 122.40 (d, *J* = 8.98 Hz), 120.01 (t), 120.00 (s, **b**), 113.32, 66.10, 56.04 (**a**), 34.24 (*“t”*, *J* = 9.36 Hz, Cy–C-P), 33.64 (*“t”*, *J* = 6.74 Hz, Cy–C-P), 27.72–27.29 (Cy), 26.88–26.53 (Cy). **ESI-QTOF (+)** *m*/*z* = [M^+^] calculated for C_51_H_64_ClCuN_3_OP_2_ 894.3503; found 894.3535.

## Supplementary Material



## Notes

NMR spectra, mass spectra, X-ray crystal bond distances and angles,), TGA curves, photophysical studies (emission, excitation spectra, lifetime fitting curves, 1931 CIE color coordinates and temperature dependence of the emission lifetime), buried volume percentages and voltametries (PDF)
