# Supplementary material for: Diphosphane-Mediated Control of the Emissive Properties in [Cu(NHC)(P^P)]+ Complexes: TADF vs Phosphorescence
Source: Inorg Chem. 2025 Oct 22;64(43):21541–50. doi: 10.1021/acs.inorgchem.5c03511 (PMC12587383; doi:10.1021/acs.inorgchem.5c03511)
Supplement: Supplementary file 1 [file ic5c03511_si_001.pdf]

Supporting information

Diphosphane-Mediated Control of the Emissive Properties in $[Cu(NHC)(P^\wedge P)]^+$ Complexes. TADF vs. Phosphorescence

Raquel Jiménez[a], Olga Crespo*[a] and M. Concepción Gimeno*[a]

[a] Departamento de Química Inorgánica, Instituto de Síntesis Química y Catálisis Homogénea (ISQCH). Universidad de Zaragoza-CSIC. E-50009 Zaragoza, Spain.

S1.- NMR spectra **S2**

S2- Mass Spectra **S16**

S3.- Crystal X-ray data: Bond distances (Å) and angles (°) **S18**

S4.- TGA curves **S19**

S5.- Emission and excitation spectra **S20**

S6.- 1931 CIE color coordinates **S23**

S7.- Lifetime fitting curves **S24**

S8.- Temperature dependence of the emission lifetime for selected complexes **S42**

S9.- Buried volume percentage **S43**

S10.- Voltametries **S44**

S1.- RMN SPECTRA

COMPOUND Cu1

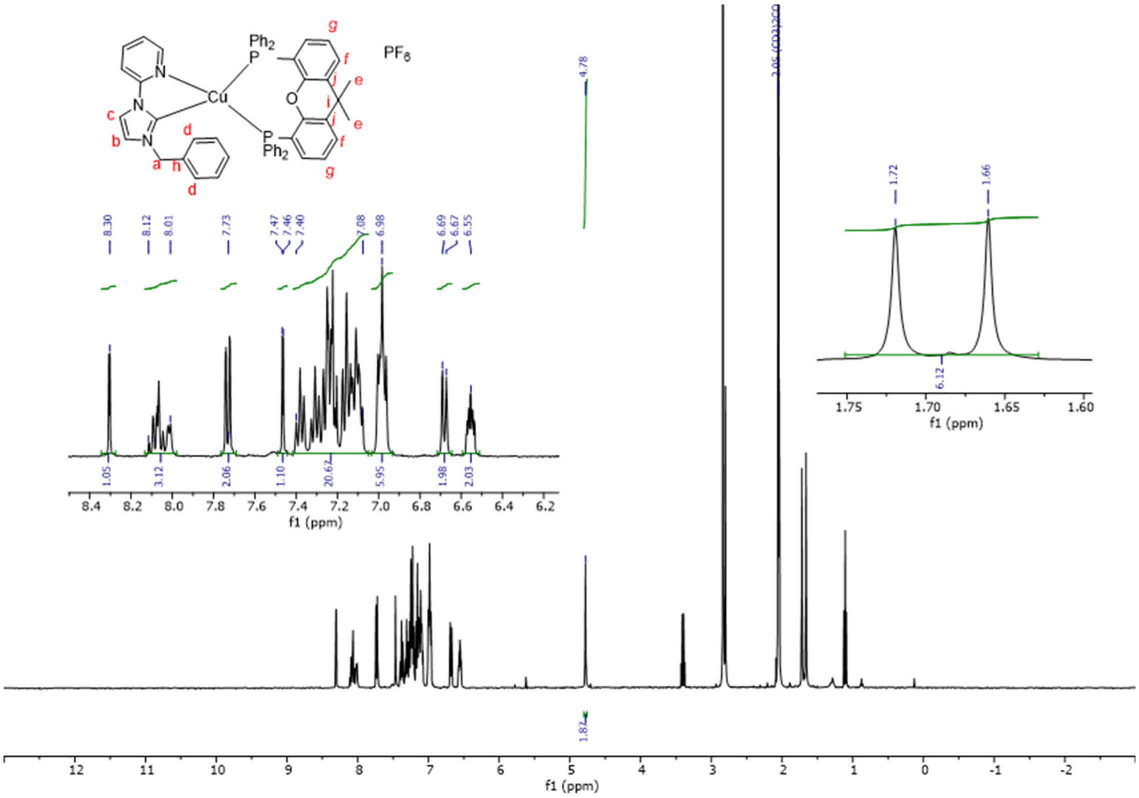

Figure S1. *[1] H-NMR* of **Cu1**.

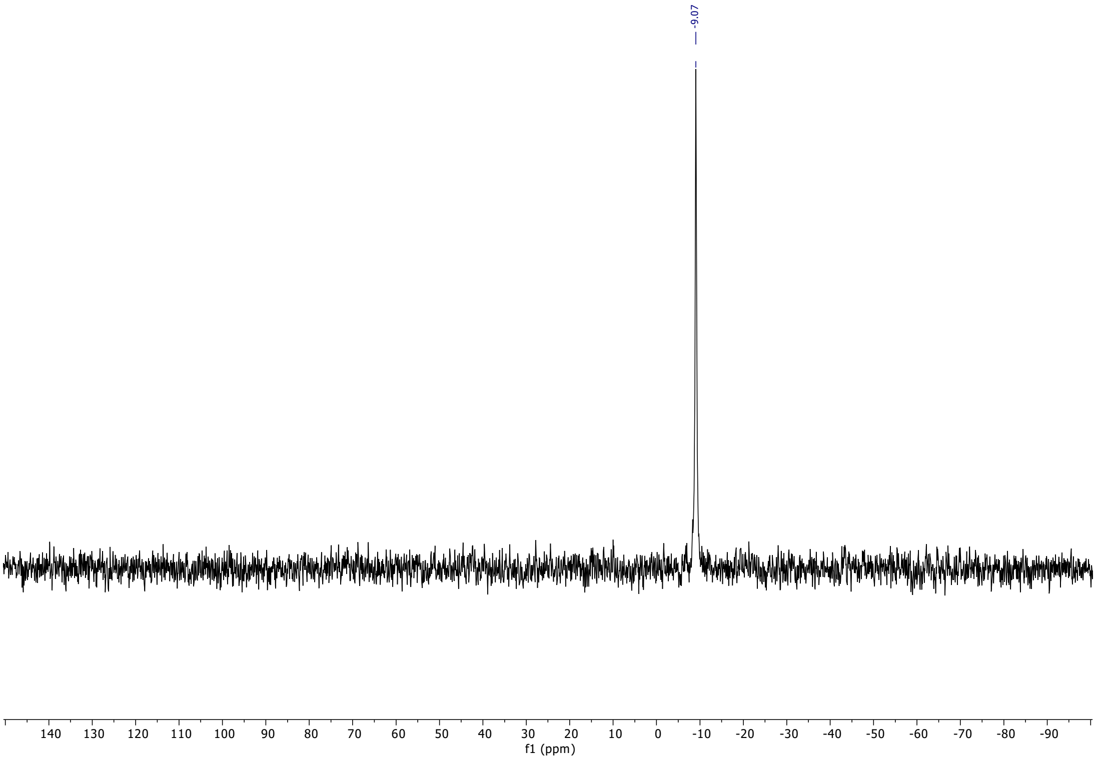

Figure S2. *^{31}P{^{1}H}-NMR* of **Cu1**.

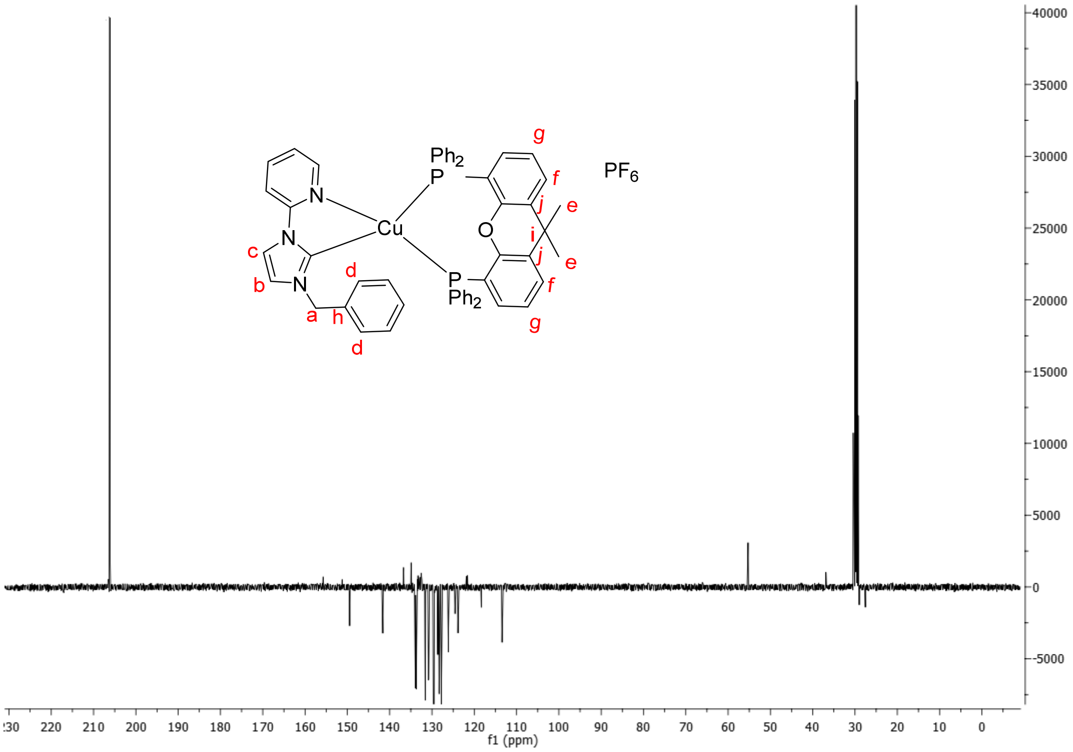

Figure S3. $^{13}C\{^{1}H\}$ *APT-NMR* of **Cu1**.

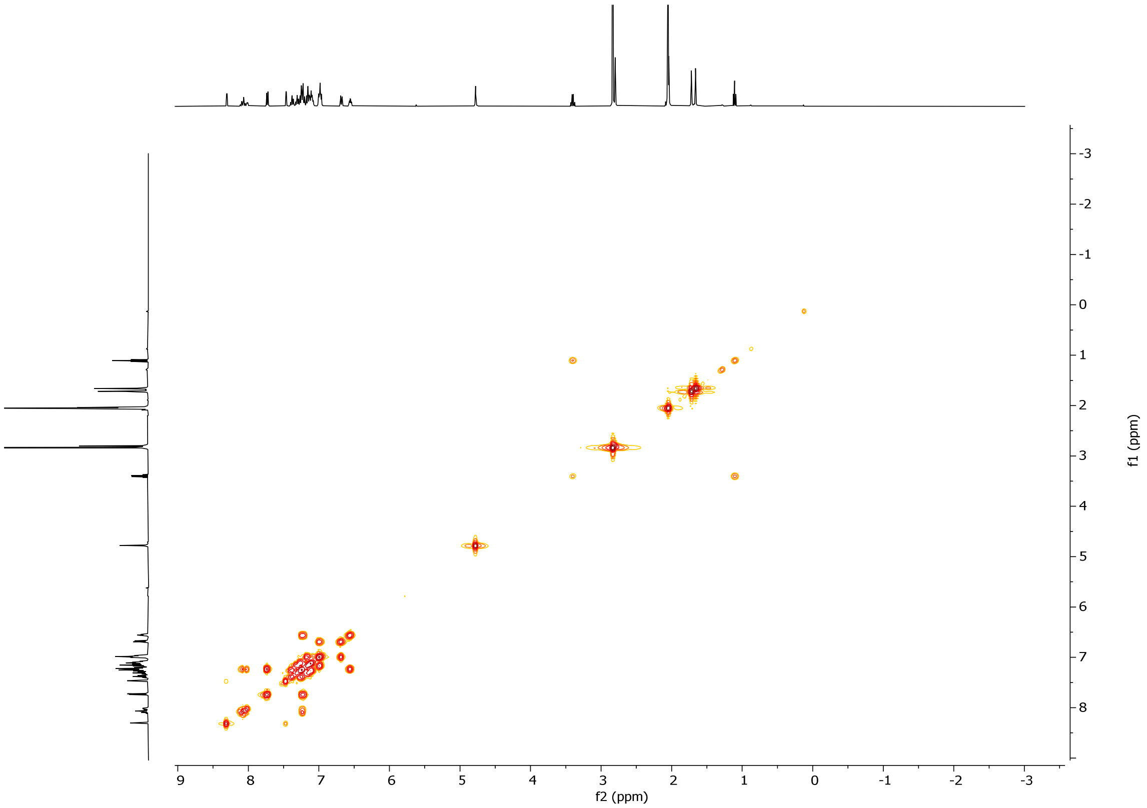

Figure S4. *COSY* ^{1}H-^{1}H of **Cu1.**

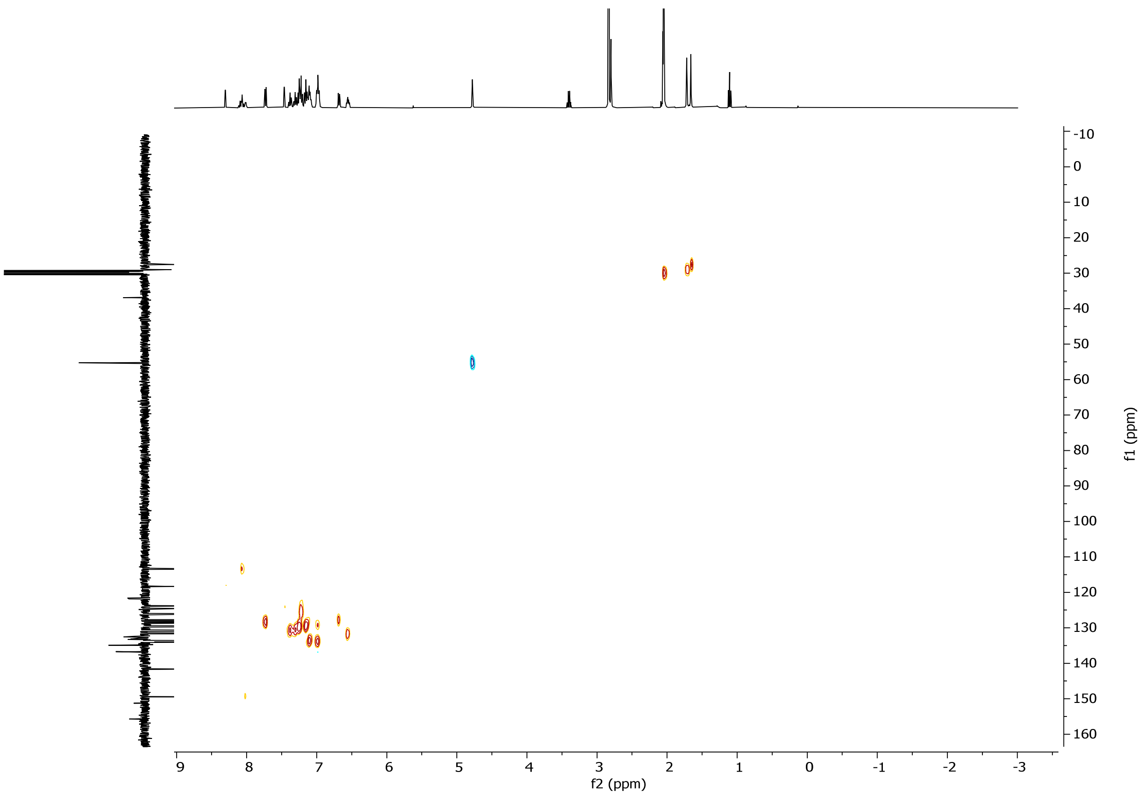

Figure S5. *HSQC* [1]*H*-[13]*C* of **Cu1.**

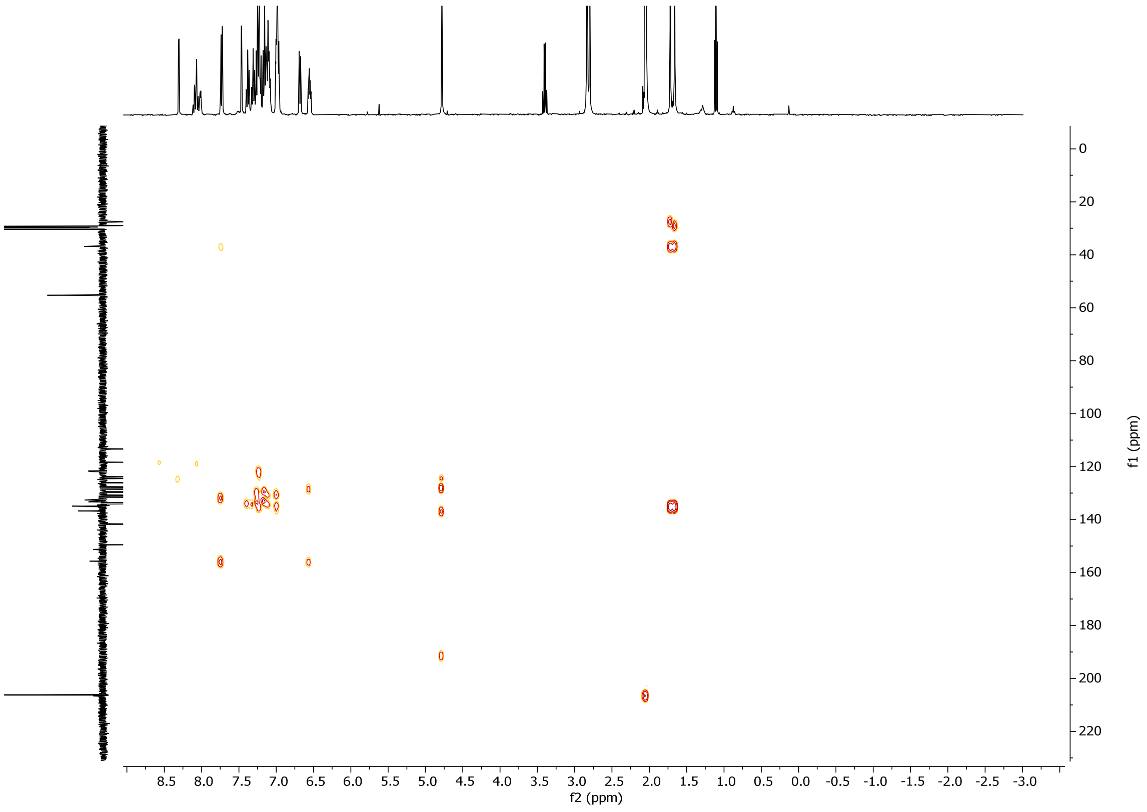

Figure S6. *HMBC* [1]*H*-[13]*C* of **Cu1.**

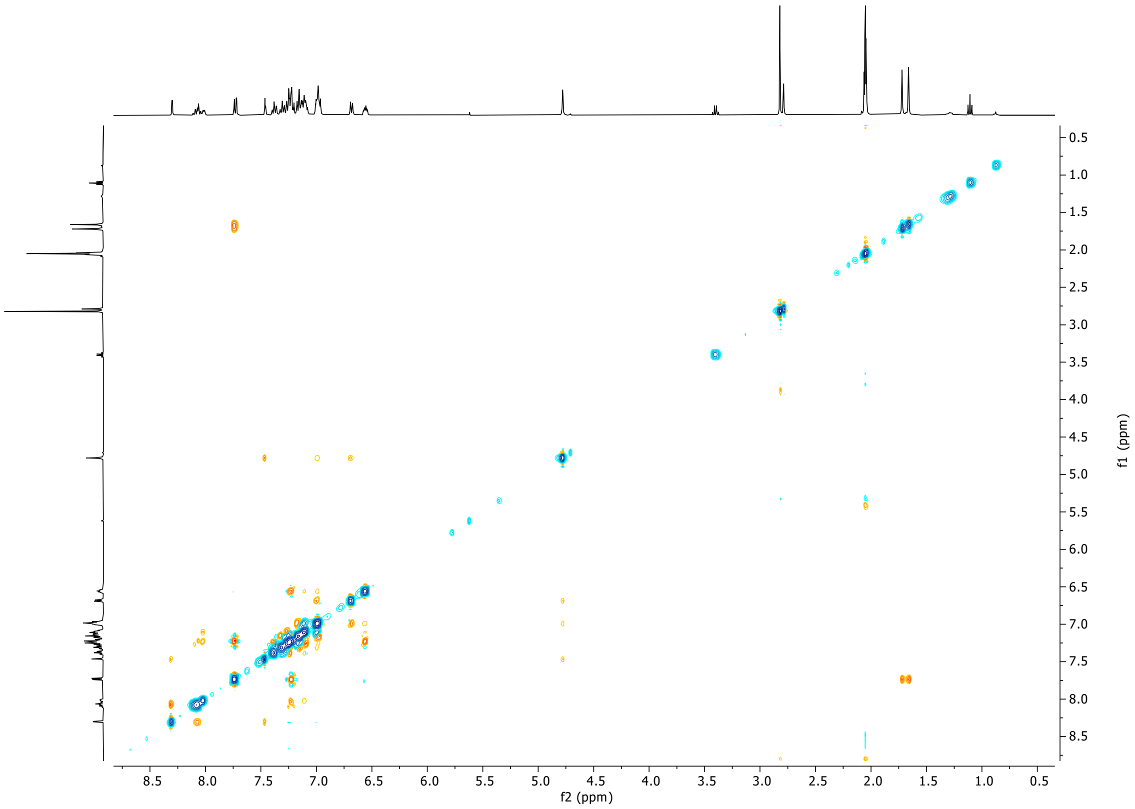

Figure S7. *NOESY ^{1}H-^{1}H* of **Cu1.**

COMPOUND Cu2

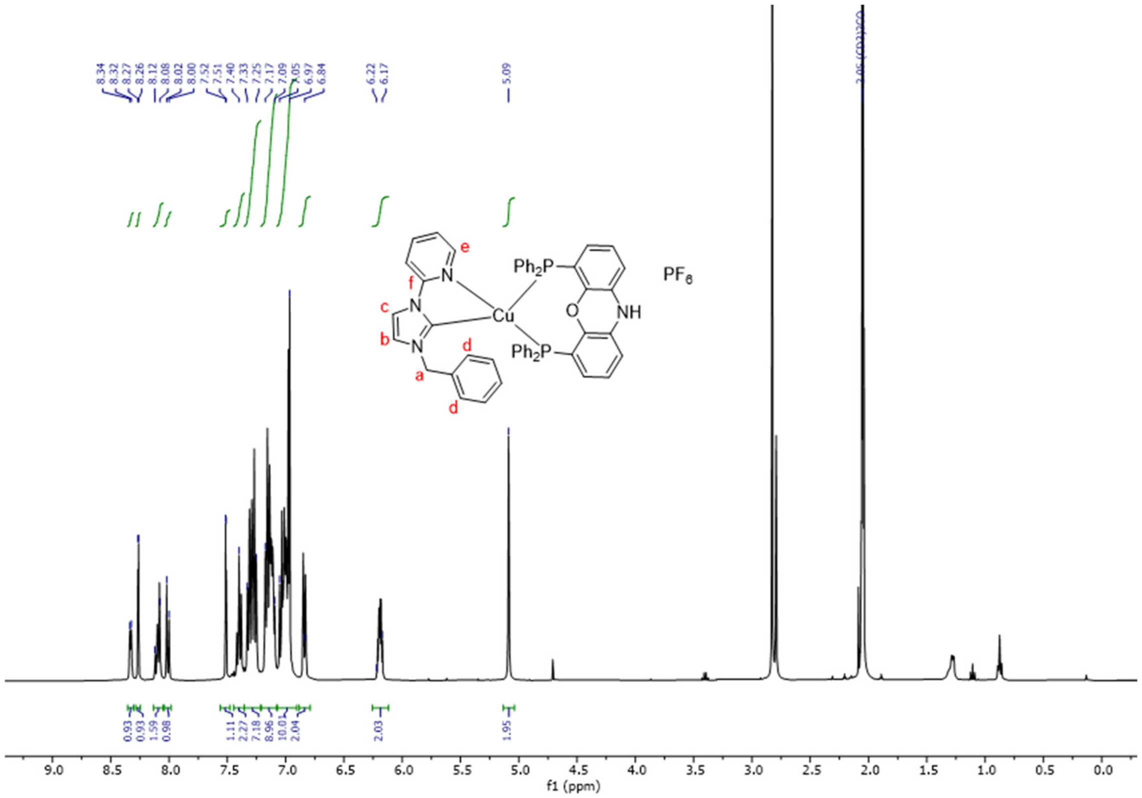

Figure S8. *^{1}H-NMR* of **Cu2**.

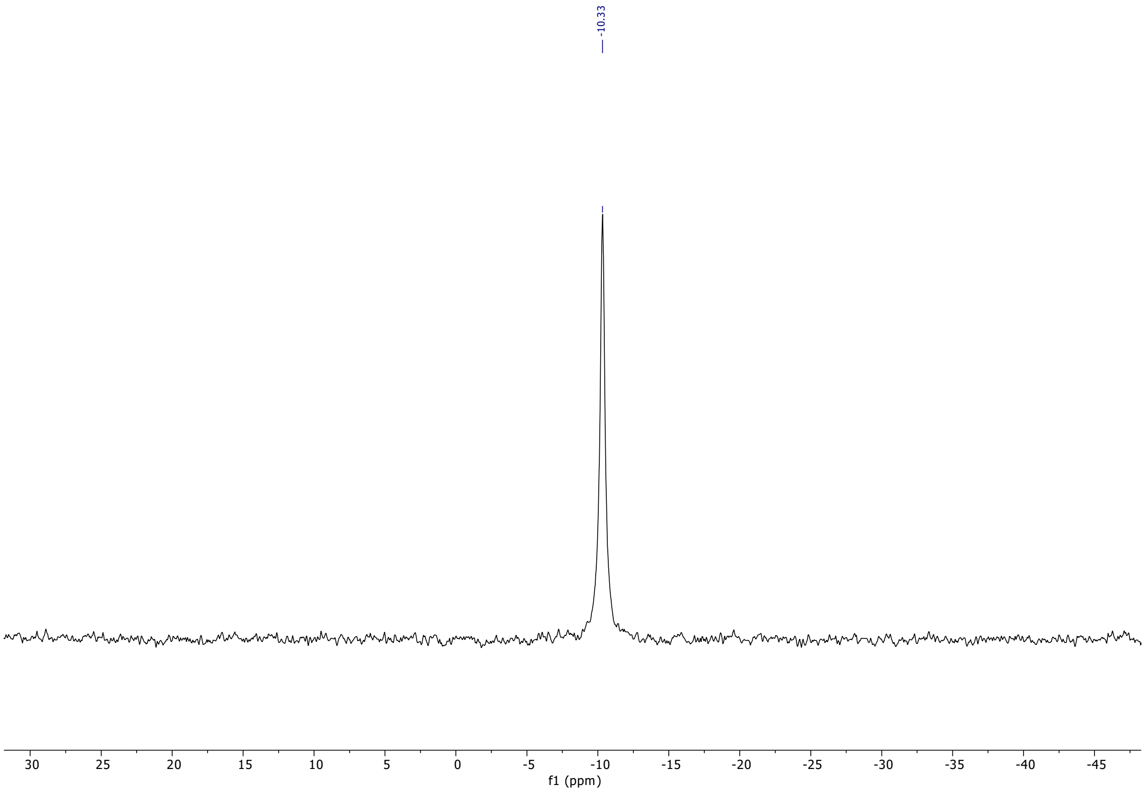

Figure S9. $^{31}P\{^{1}H\}$*-NMR* of **Cu2**.

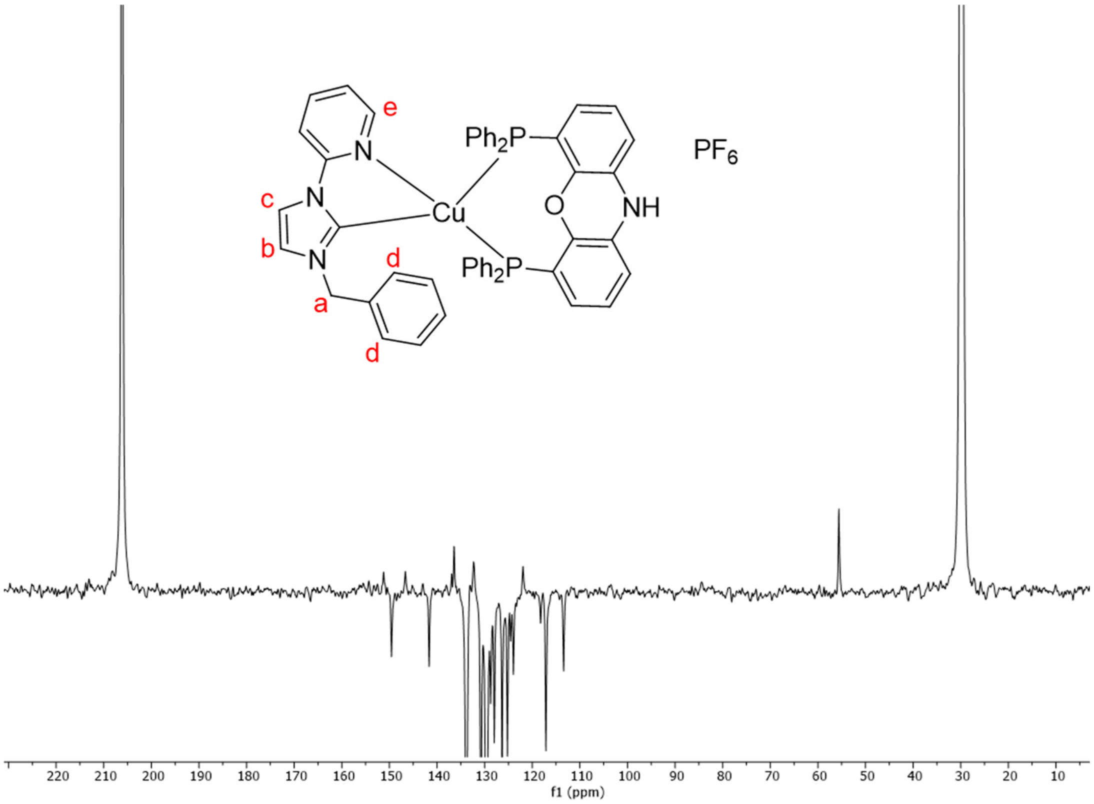

Figure S10. $^{13}C\{^{1}H\}$*-NMR* of **Cu2**.

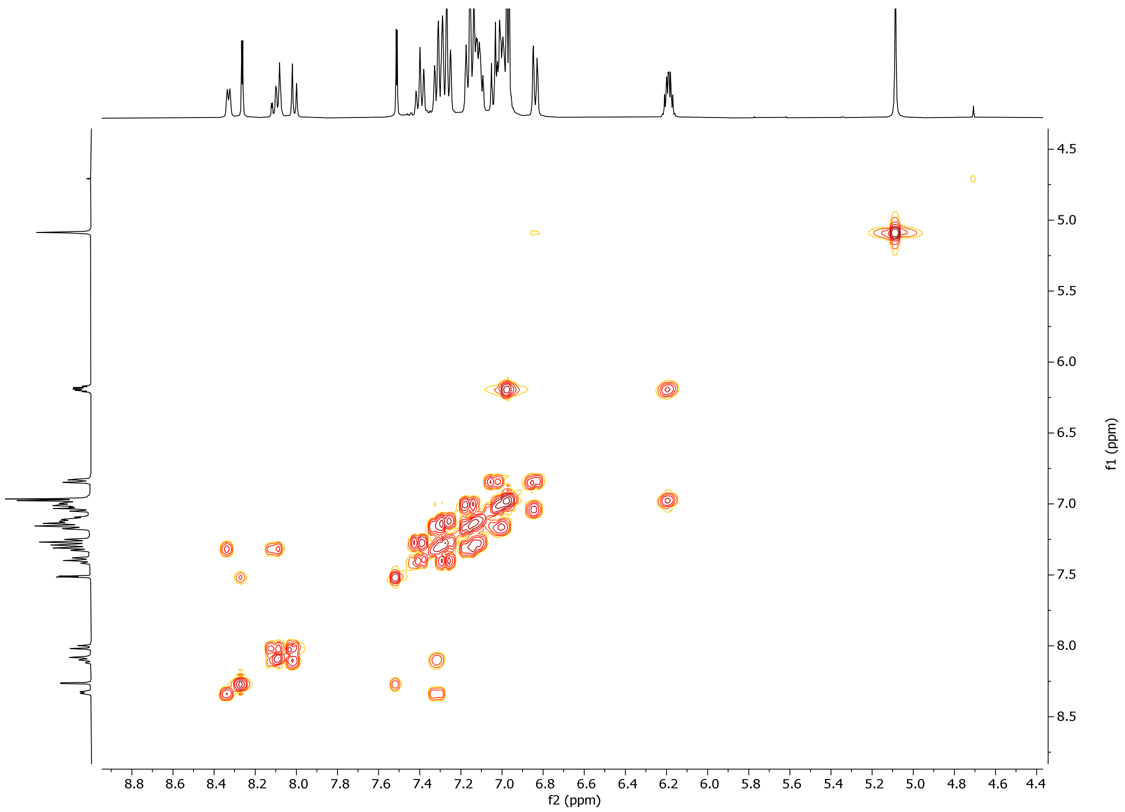

Figure S11. *COSY ^{1}H-^{1}H* of **Cu2.**

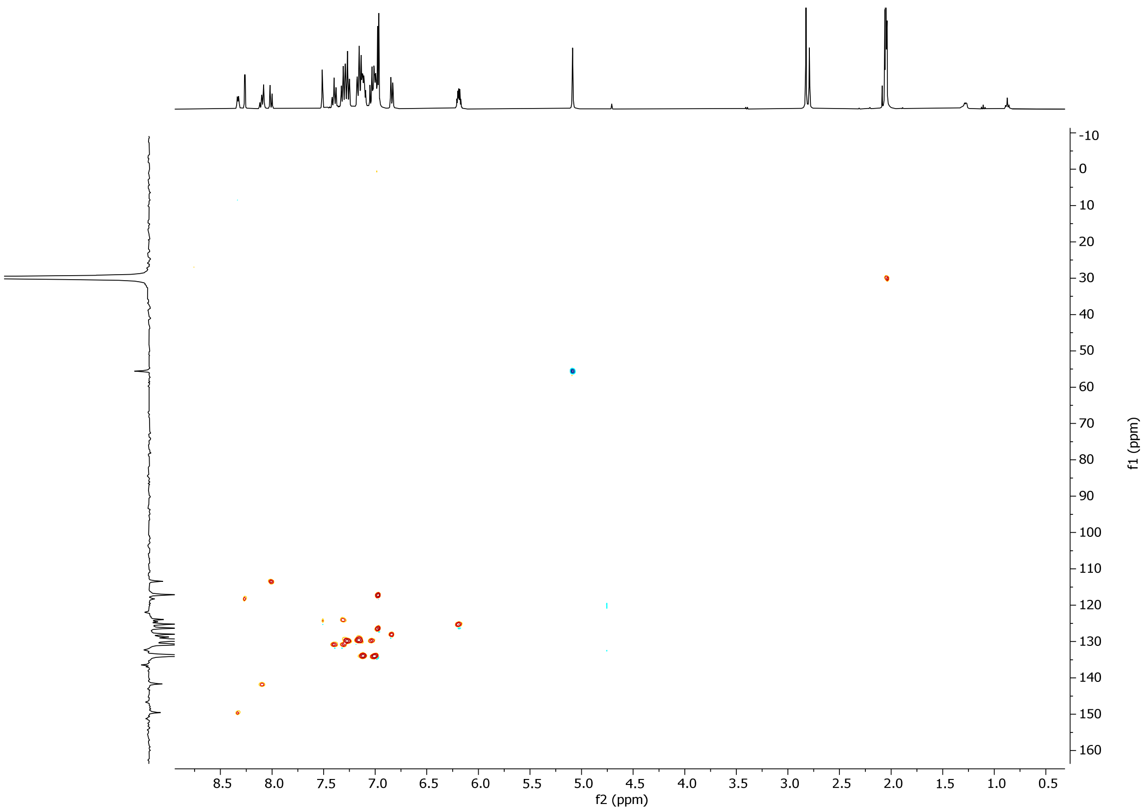

Figure S12. *HSQC ^{1}H-^{13}C* of **Cu2.**

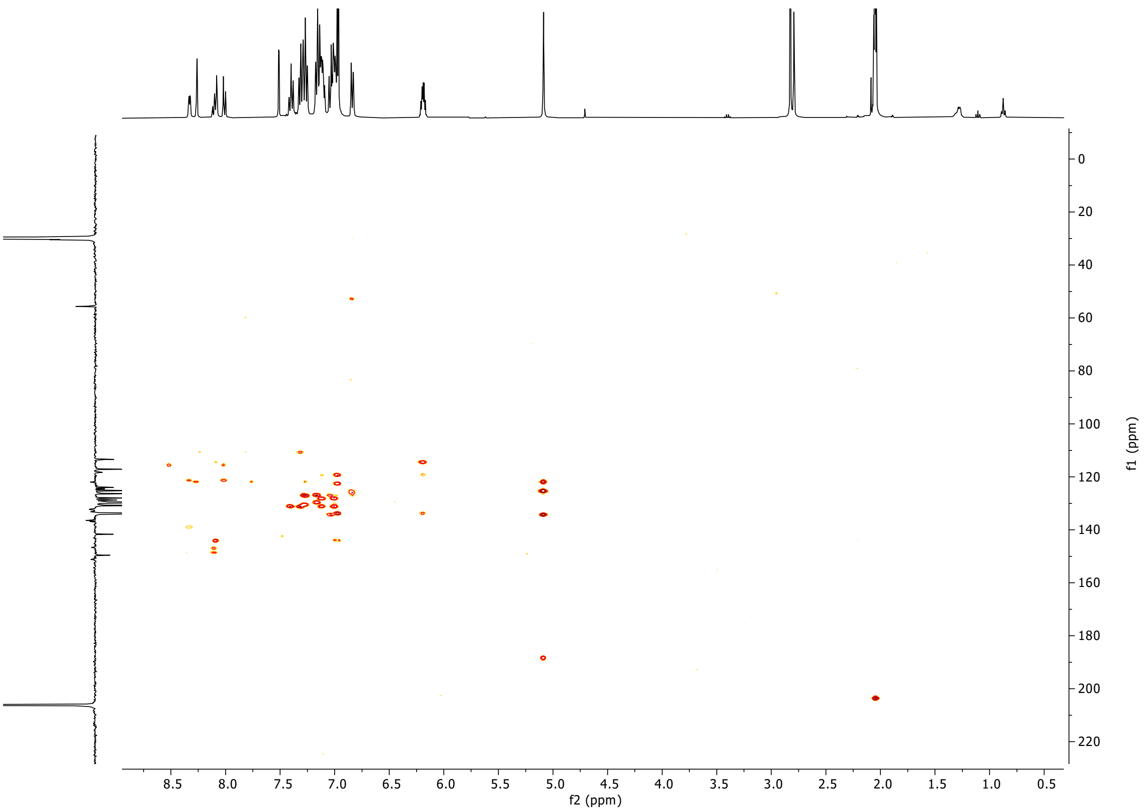

Figure S13. *HMBC ^{1}H-^{13}C* of **Cu2.**

COMPOUND 3

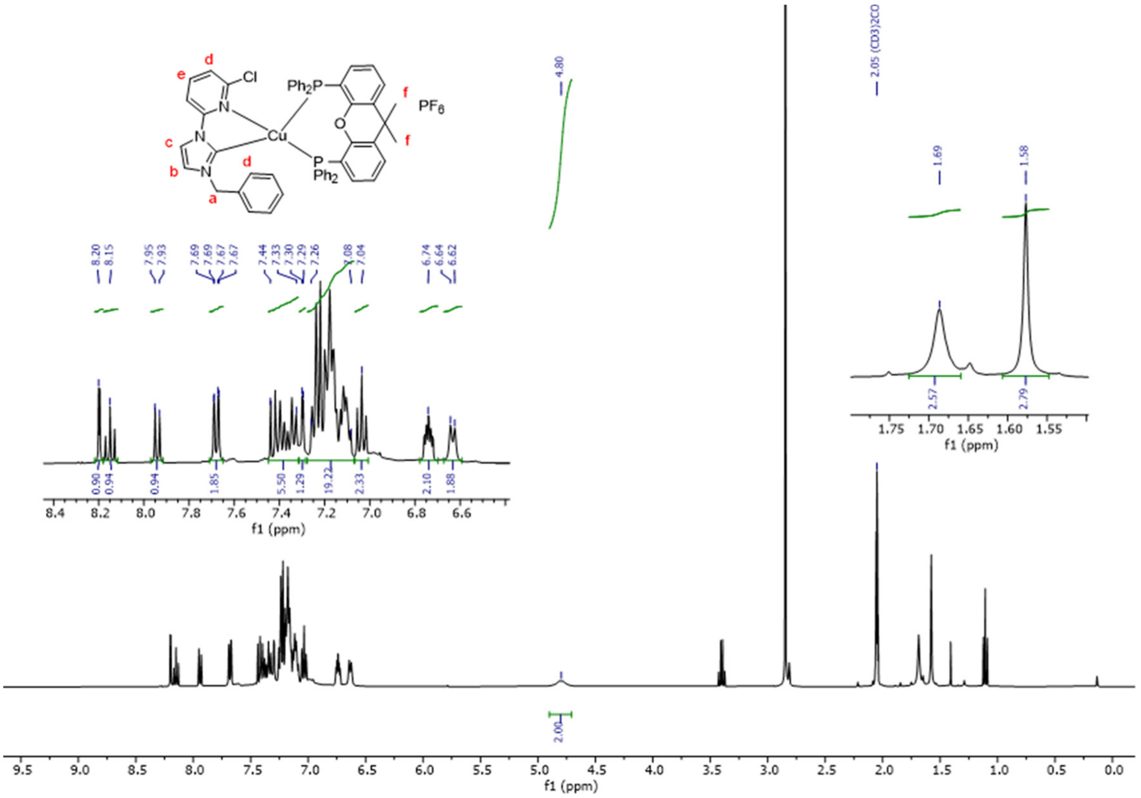

Figure S14. *^{1}H-NMR* of **Cu3**.

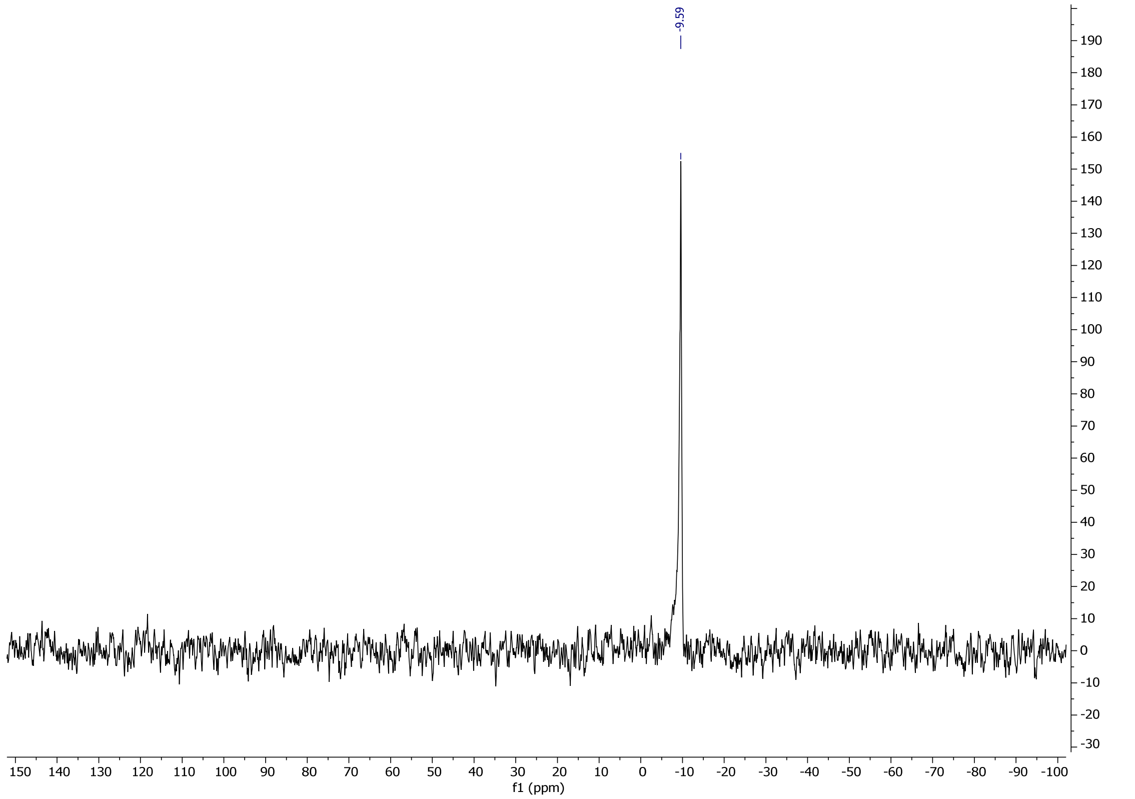

Figure S15. *$^{31}P\{^{1}H\}$-NMR* of **Cu3**.

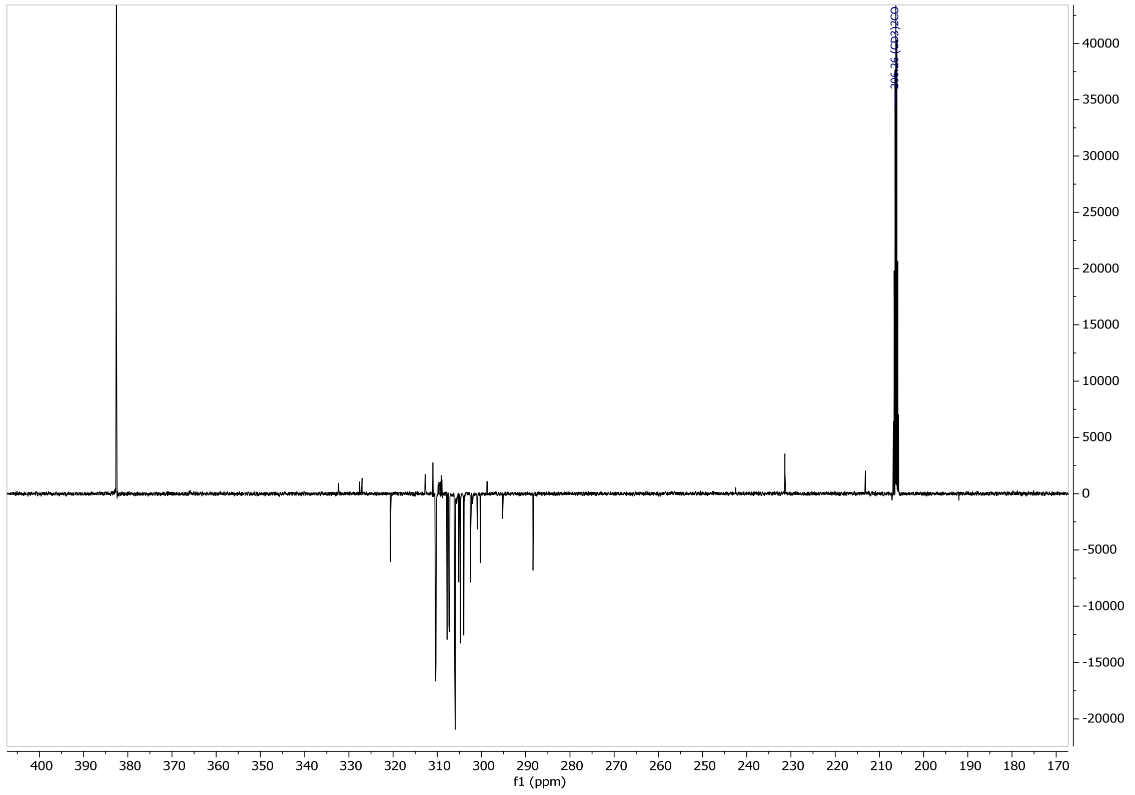

Figure S16. *$^{13}C\{^{1}H\}$-ATP-NMR* of **Cu3**.

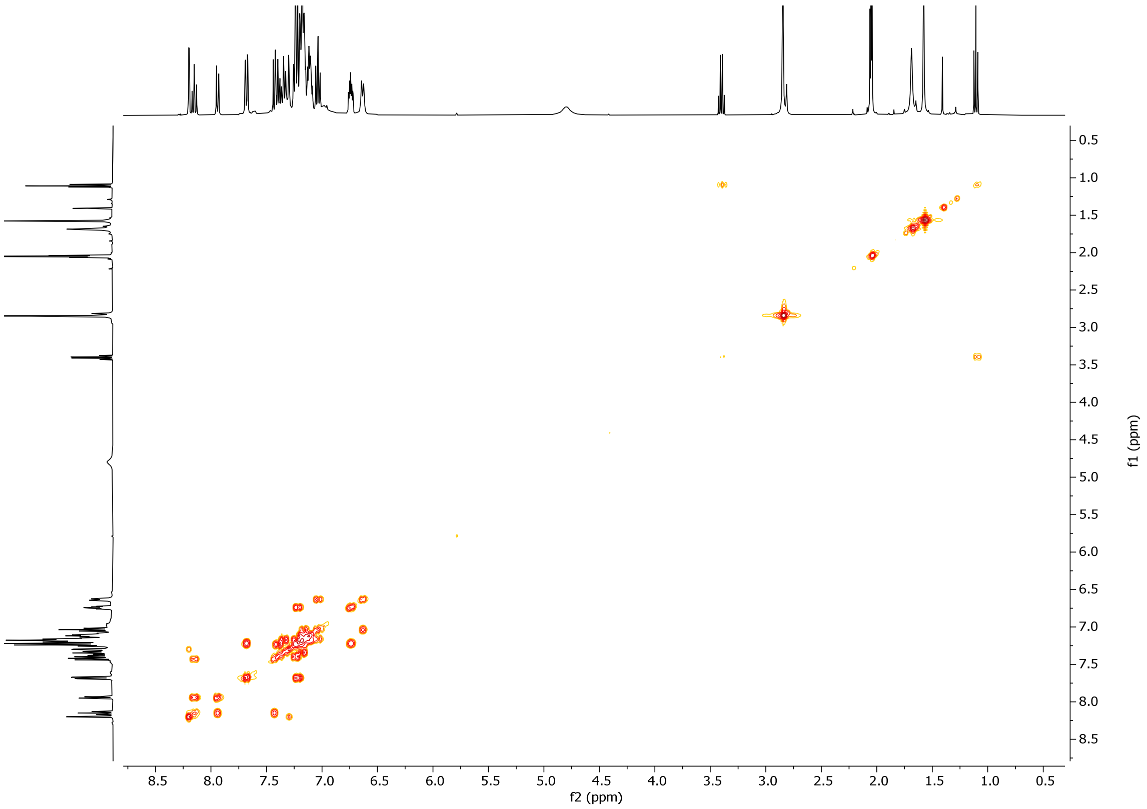

Figure S17. *COSY* ^{1}H-^{1}H of **Cu3**.

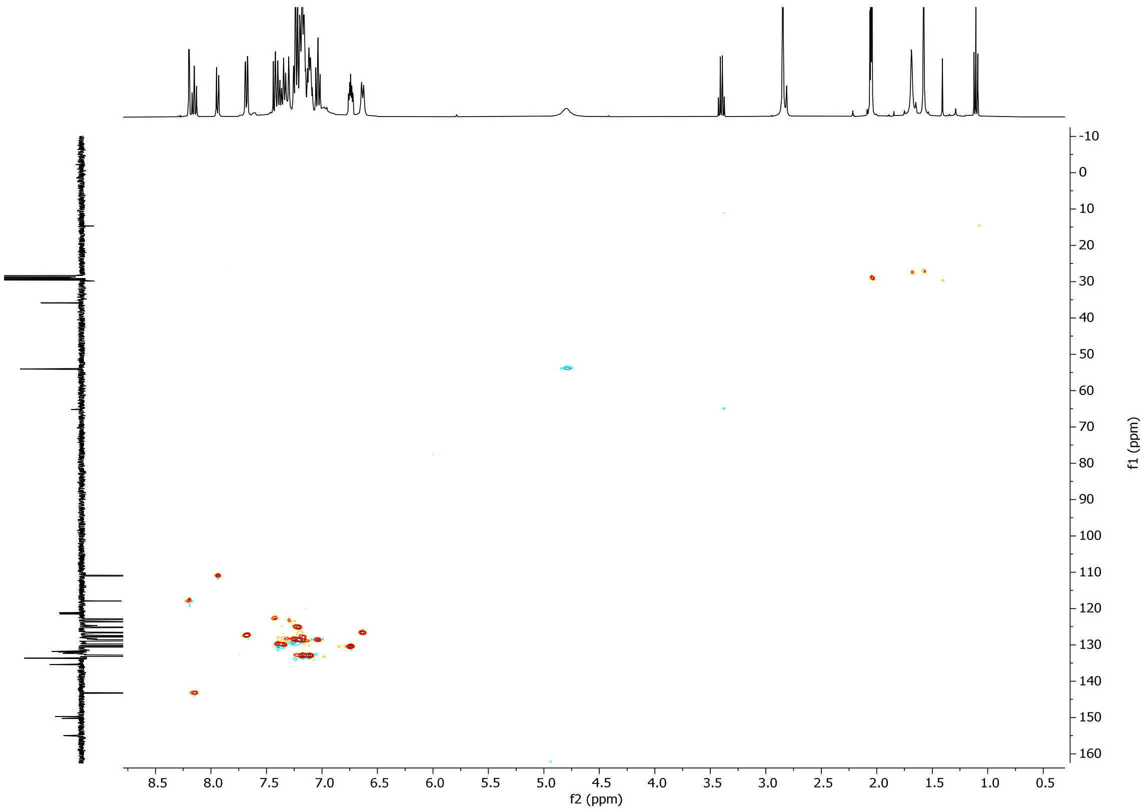

Figure S18. *HSQC* ^{1}H-^{13}C of **Cu3**.

COMPOUND 4

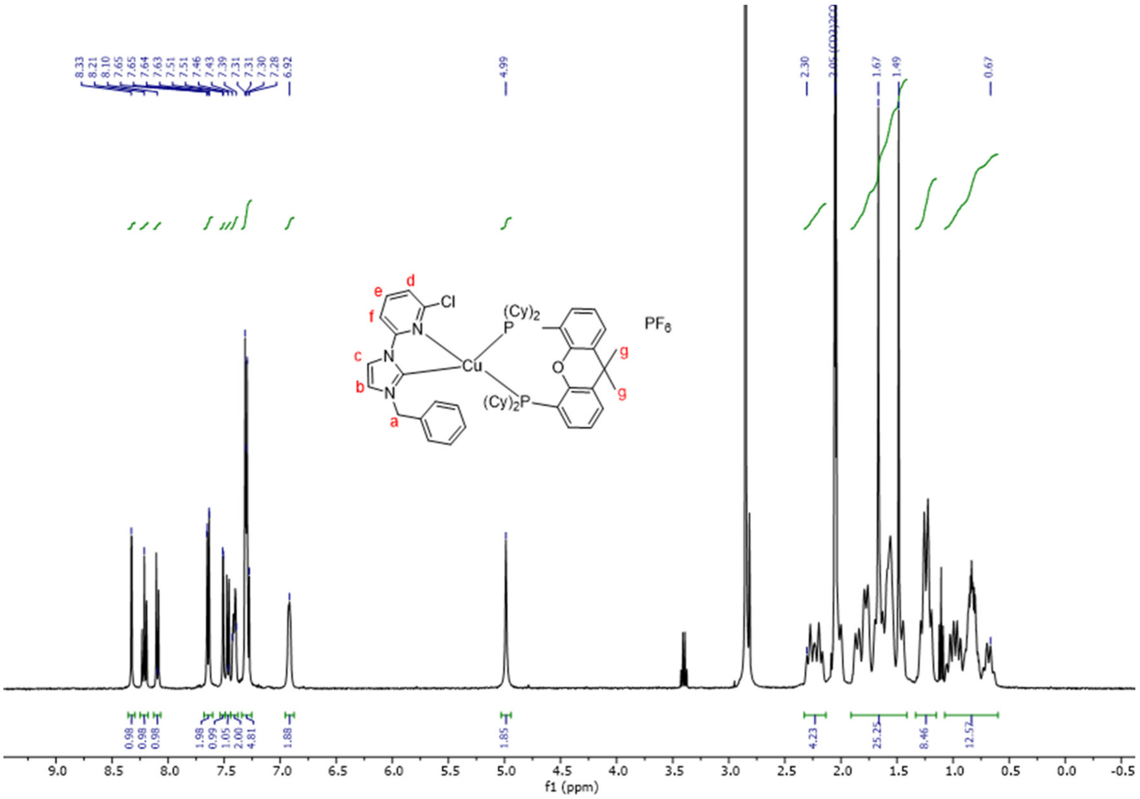

Figure S19. ^{1}H*-NMR* of **Cu4**.

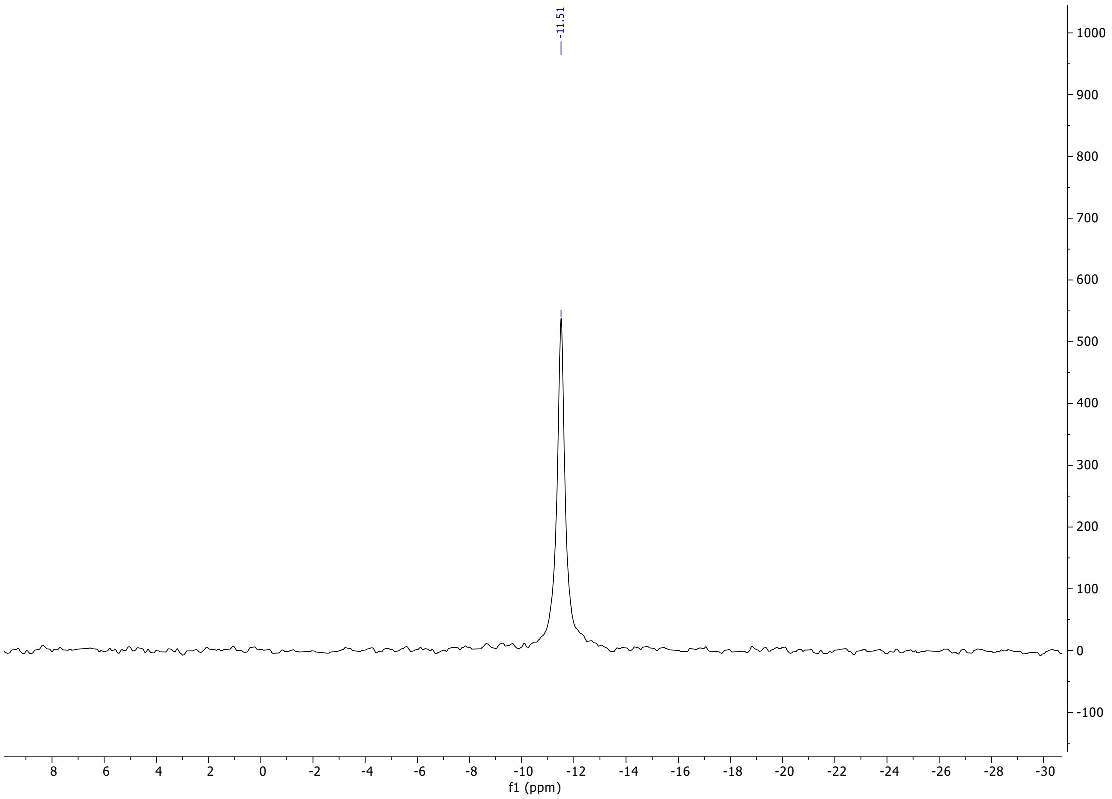

Figure S20. $^{31}P\{^{1}H\}$*-NMR* of **Cu4**.

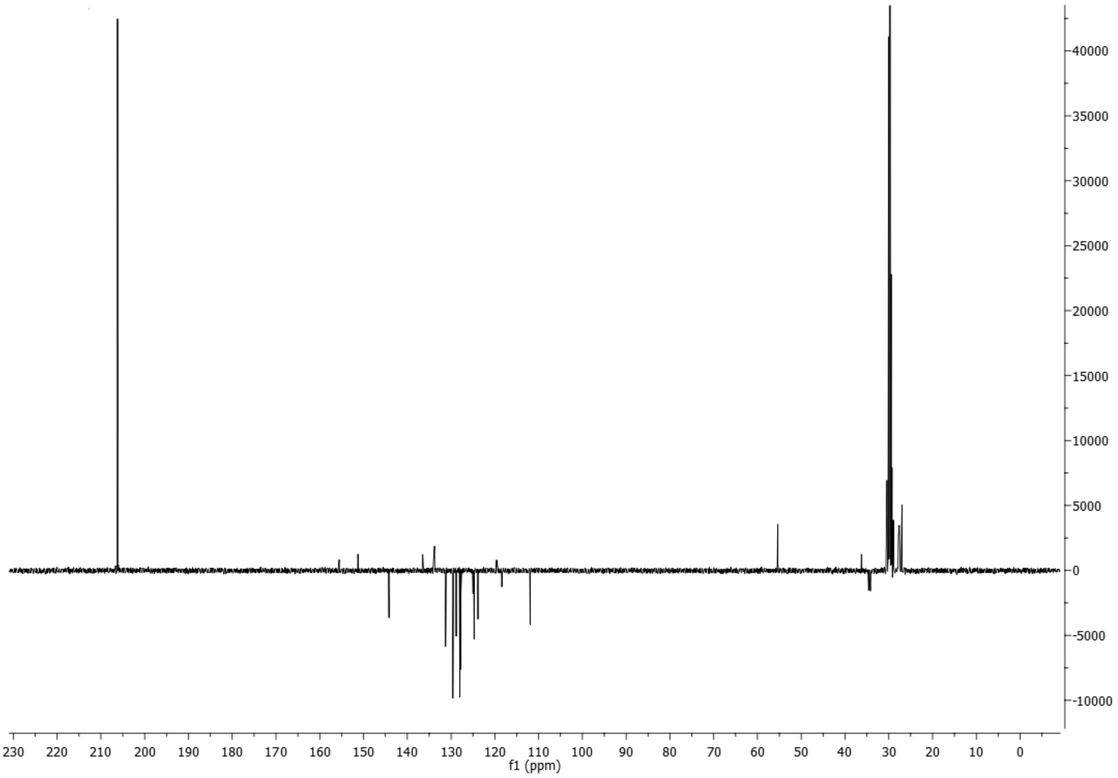

Figure S21. *$^{13}C\{^{1}H\}$-NMR* of **Cu4**.

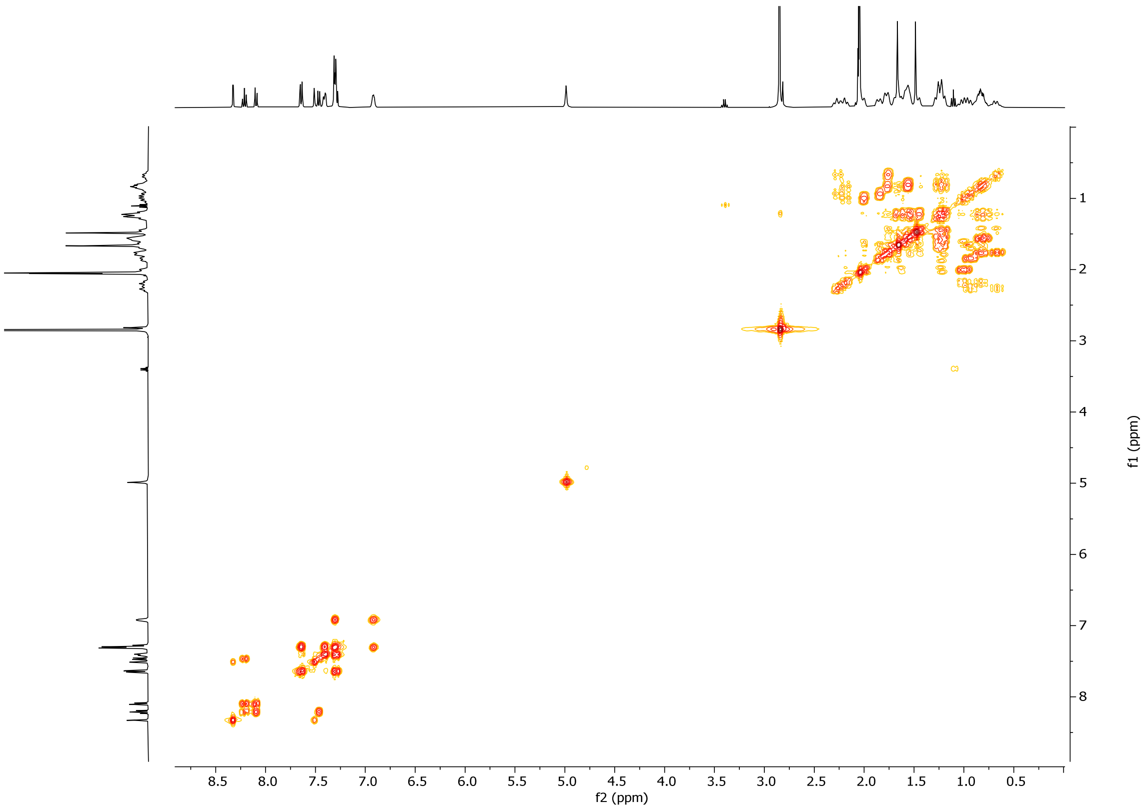

Figure S22. *COSY ^{1}H-^{1}H* of **Cu4**.

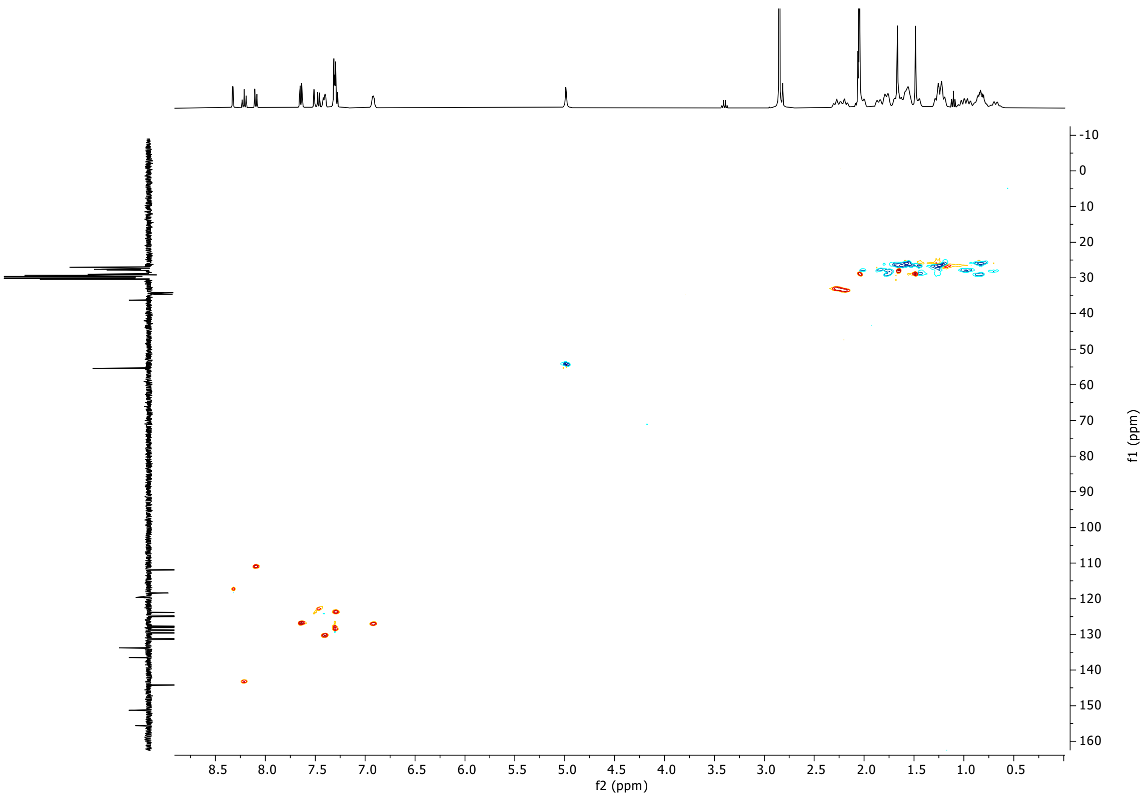

Figure S23. *HSQC ^{1}H-^{13}C* of **Cu4**.

COMPOUND 6

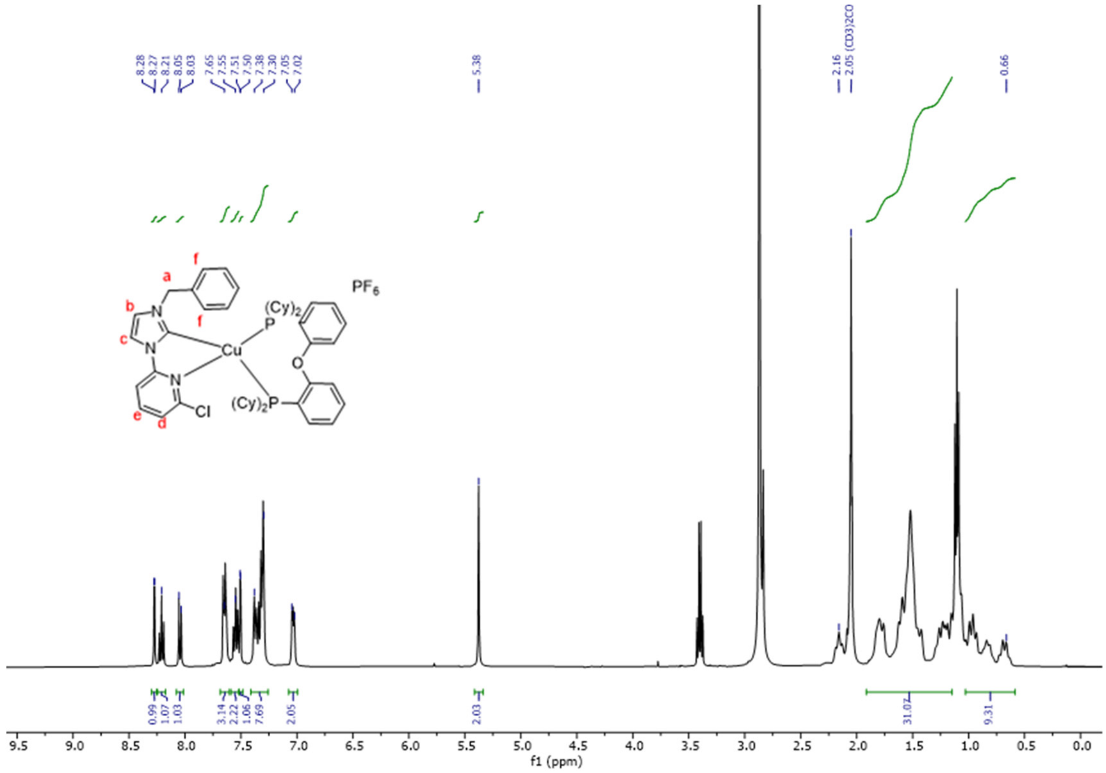

Figure S24. *^{1}H-NMR* of **Cu6**.

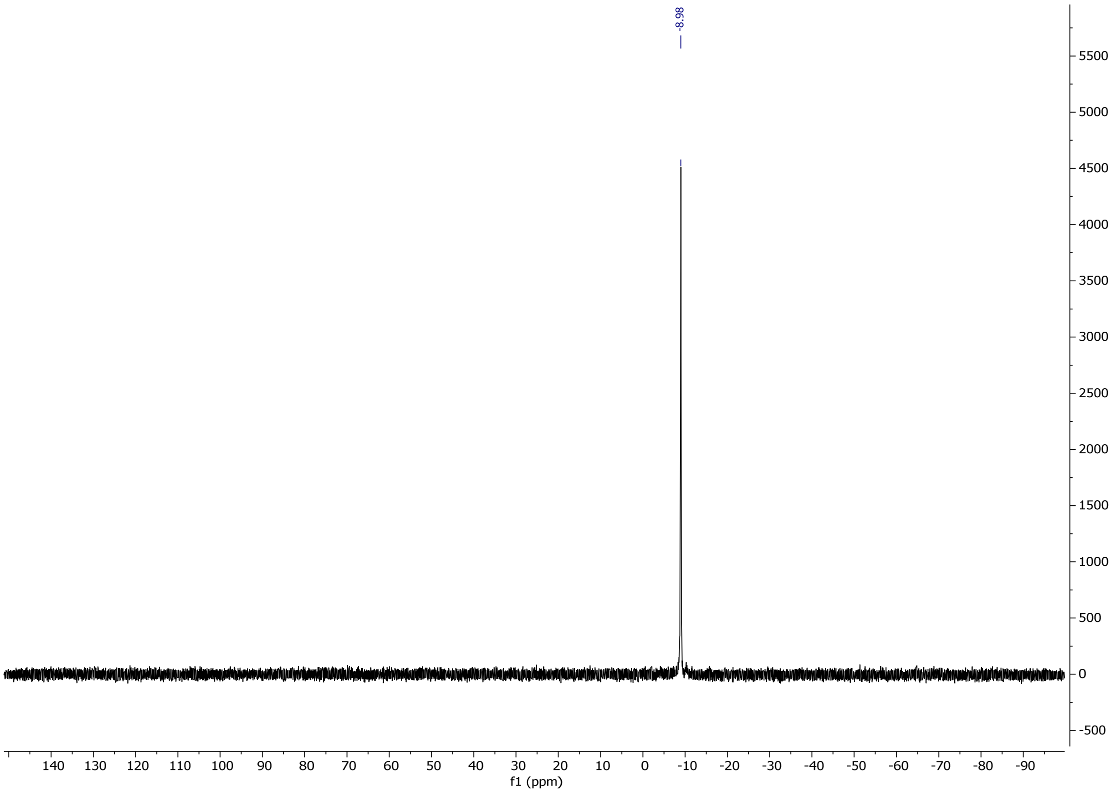

Figure S25. *$^{31}P\{^{1}H\}$-NMR* of **Cu6**.

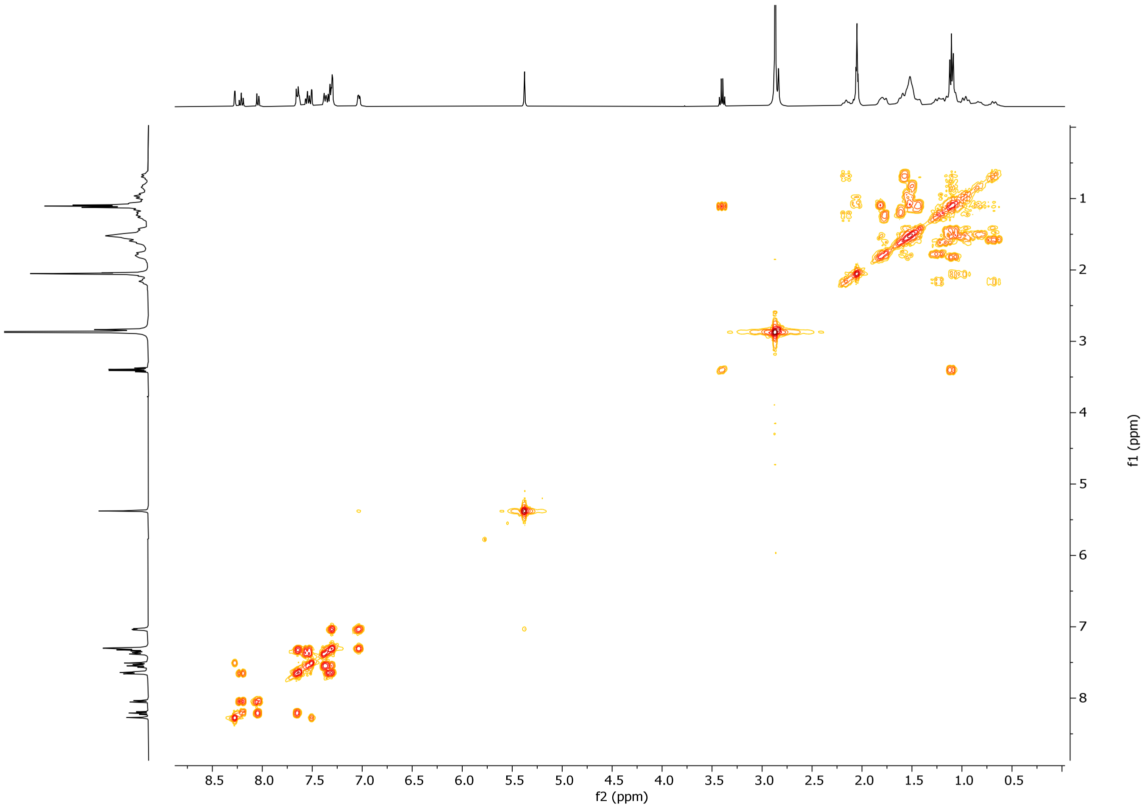

Figure S26. *COSY ^{1}H-^{1}H* of **Cu6**.

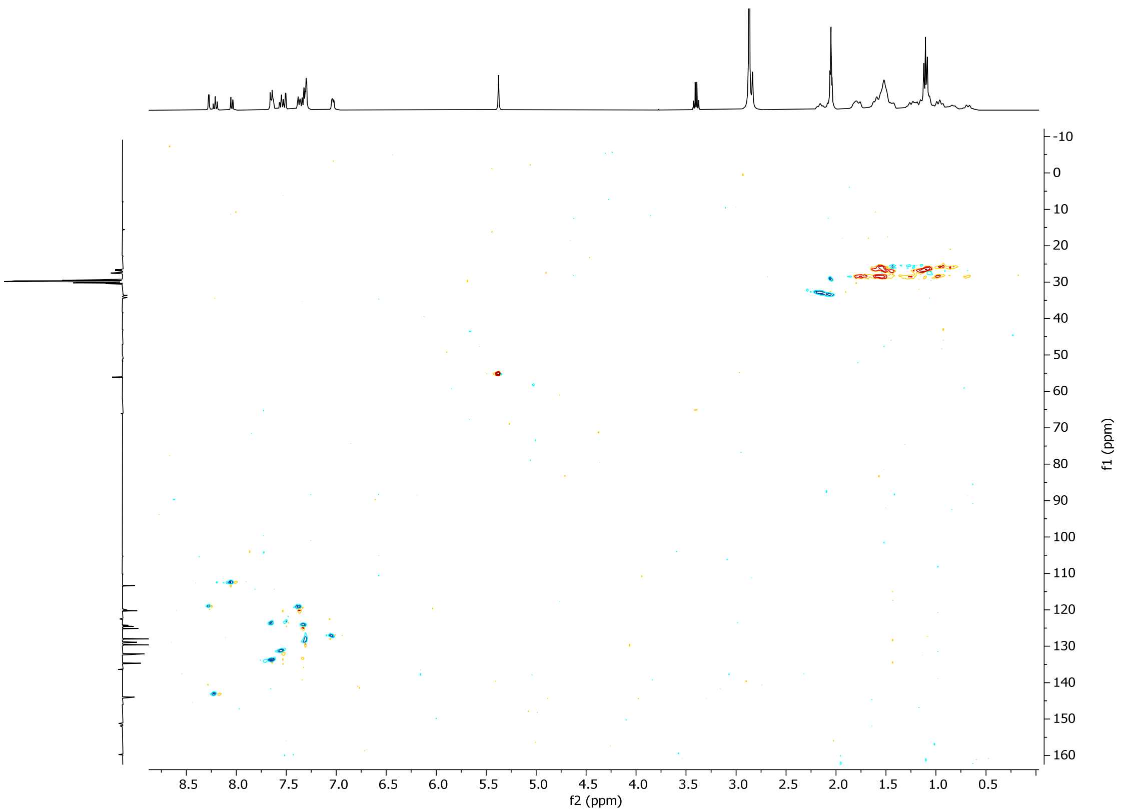

Figure S27. *HSQC ^1H-^{13}C* of **Cu6**.

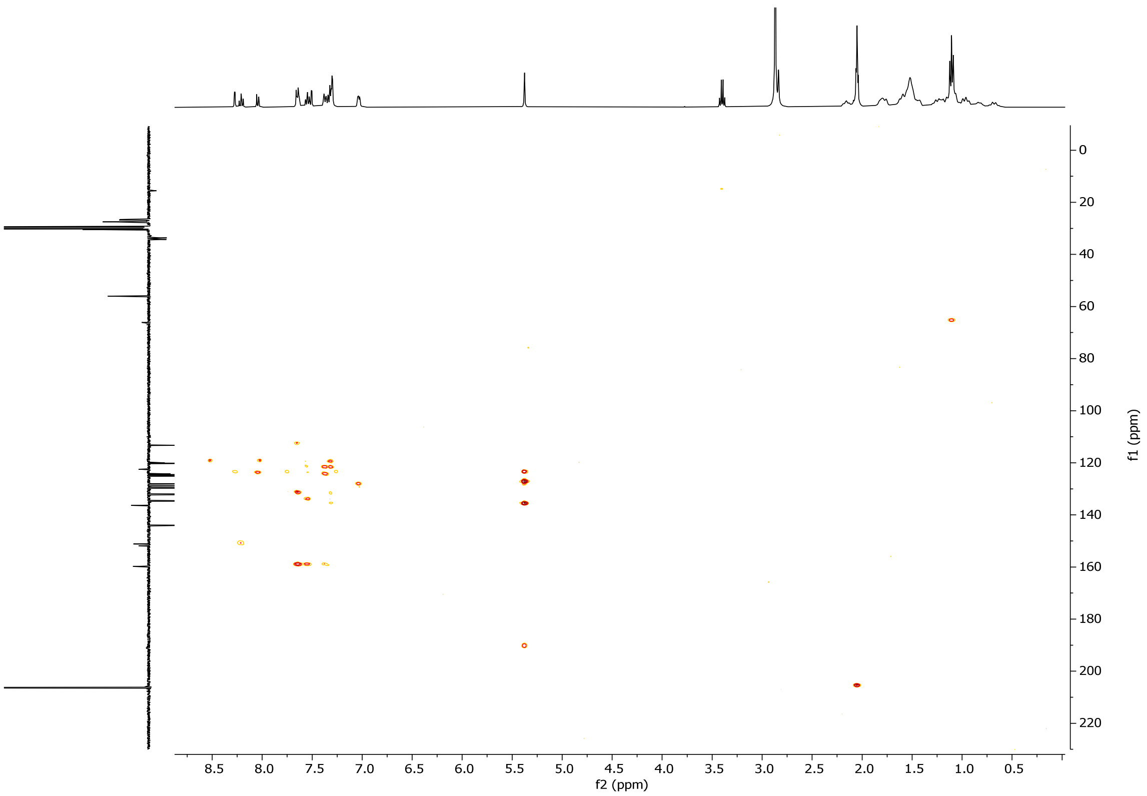

Figure S28. *HMBC ^1H-^{13}C* of **Cu6**.

S2.- MASS ESPECTRA

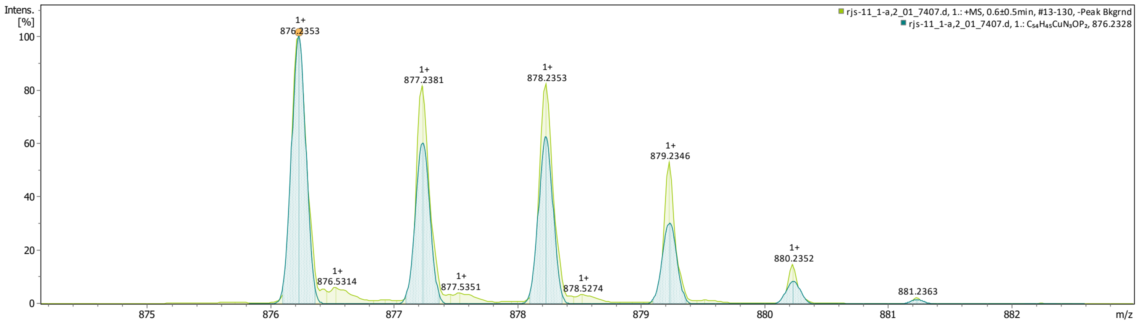

Figure S29. ***Data from ESI-QTOF (+) of Cu1***. *m/z*= [M$^+$] calculated for $C_{45}H_{45}CuN_3OP_2$ 876.2328; found 876.2353.

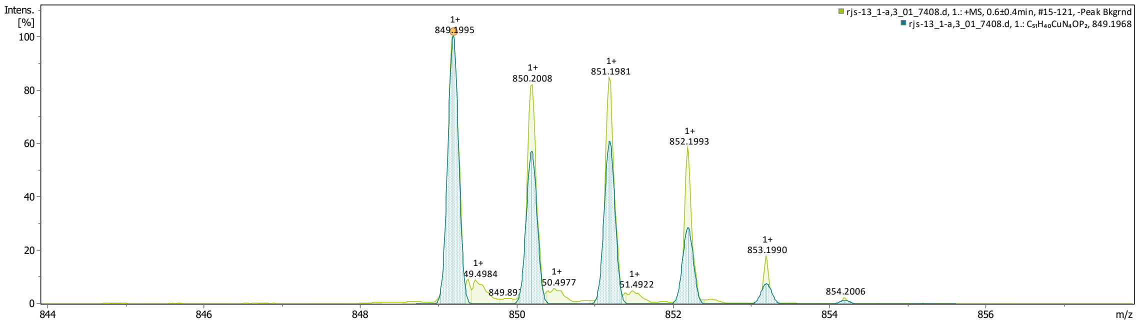

Figure S30. ***Data from ESI-QTOF (+) of Cu2***. *m/z*= [M$^+$] calculated for $C_{51}H_{40}CuN_4OP_2$ 849.1967; found 849.1995.

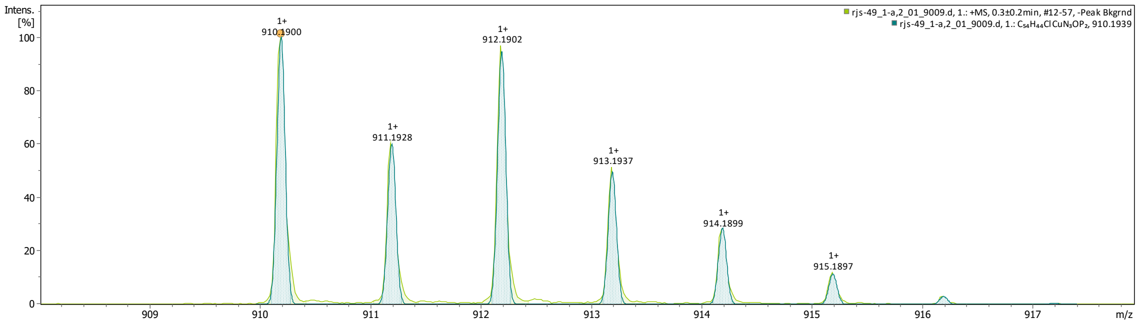

Figure S31. ***Data from ESI-QTOF (+) of Cu3.*** *m/z*= [M$^+$] calculated for $C_{59}H_{44}ClCuN_3OP_2$ 910.1938; found 910.1889.

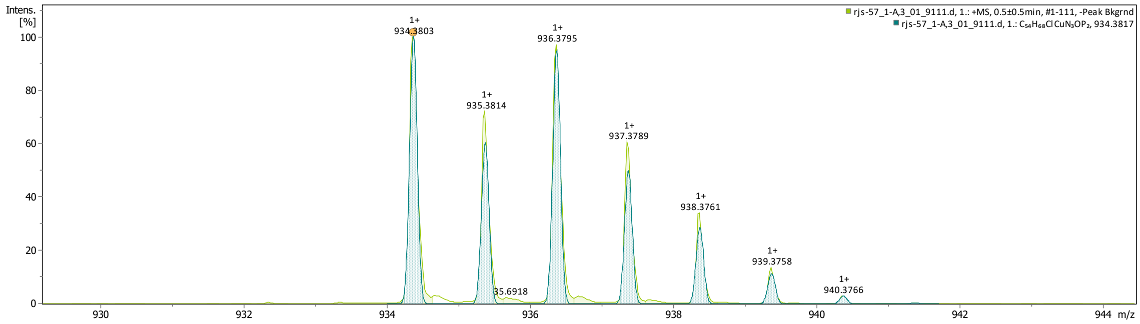

Figure S32. ***Data from ESI-QTOF (+) of Cu4.*** m/z= [M^+] calculated for $C_{54}H_{68}ClCuN_3OP_2$ 934.3816; found 934.3803.

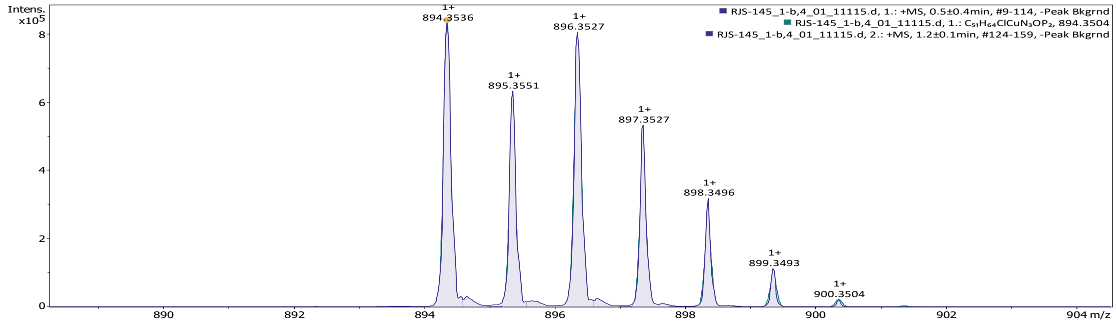

Figure S33. ***Data from ESI-QTOF (+) of Cu6.*** m/z= [M^+] calculated for $C_{51}H_{64}ClCuN_3OP_2$ 894.350364; found 894.353590.

S3.- Crystal X-ray data. Bond distances (Å) and angles (deg) for complexes **Cu1**-**Cu3**.

**Compound**	**Cu-C**	**Cu-N**	**Cu-P**	**P^Cu^P**	**C^Cu^N**
**Cu1 [Cu(L_A)(Xantphos)]$^+$**	1.966(3)	2.175(3)	2.2632(9), 2.2480(9)	112.96(4)	79.93(13)
**Cu2 [Cu(L_A)(NiXantphos)]$^+$**	1.996(6)	2.142(5)	2.2792(14),2.2553(14)	112.34(5)	80.5(2)
**Cu3 [Cu(L_B)(Xantphos)]$^+$**	1.980(2)	2.2412(19)	2.2825(6), 2.2640(6)	110.44(2)	78.84(8)

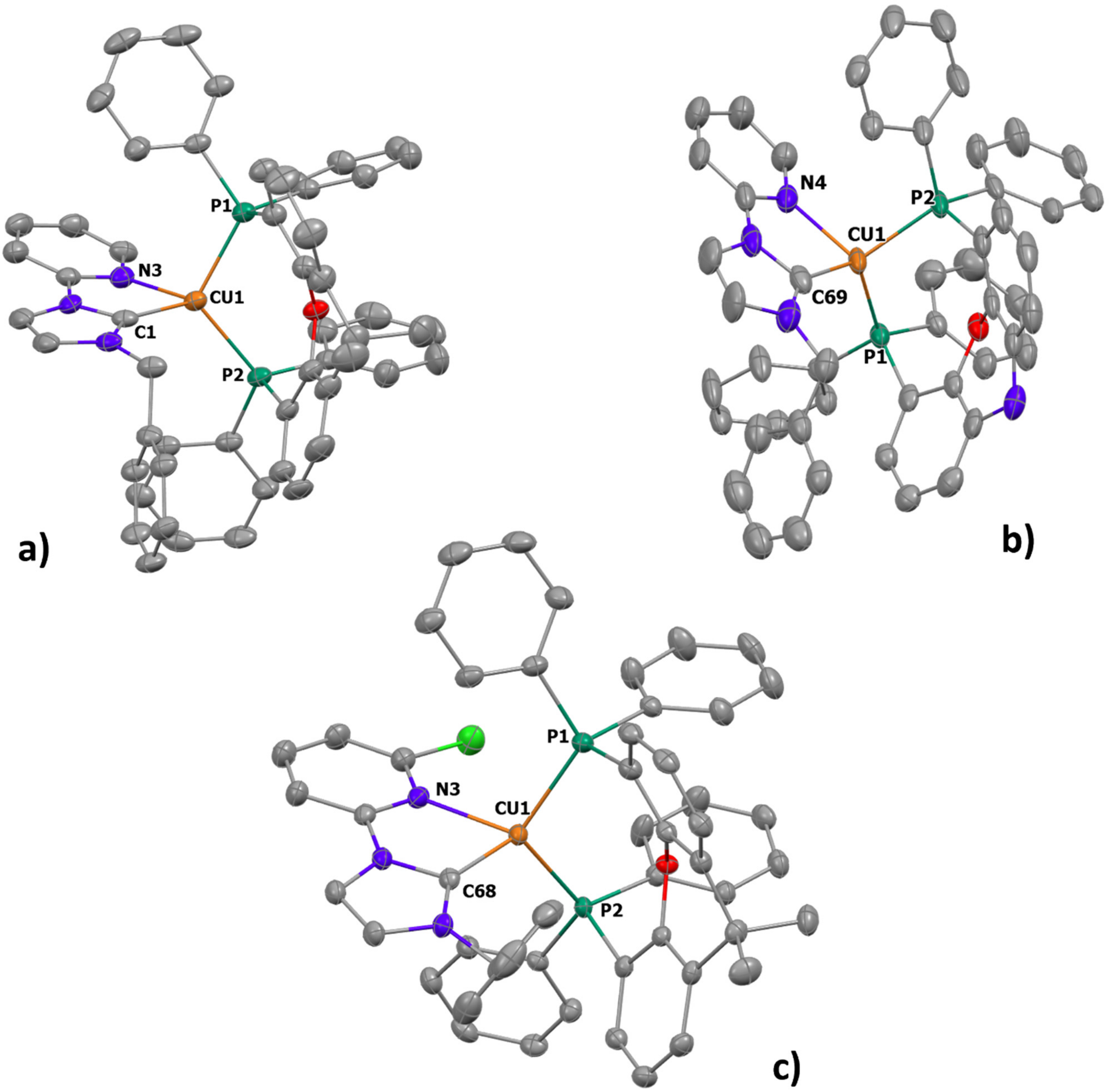

Figure S34. Ortep diagram of the cation of **Cu1** (a), **Cu2** (b), **Cu3** (c). Hydrogen atoms have been omitted for clarity. Ellipsoids represent 50% probability level.

S4.- TGA curves

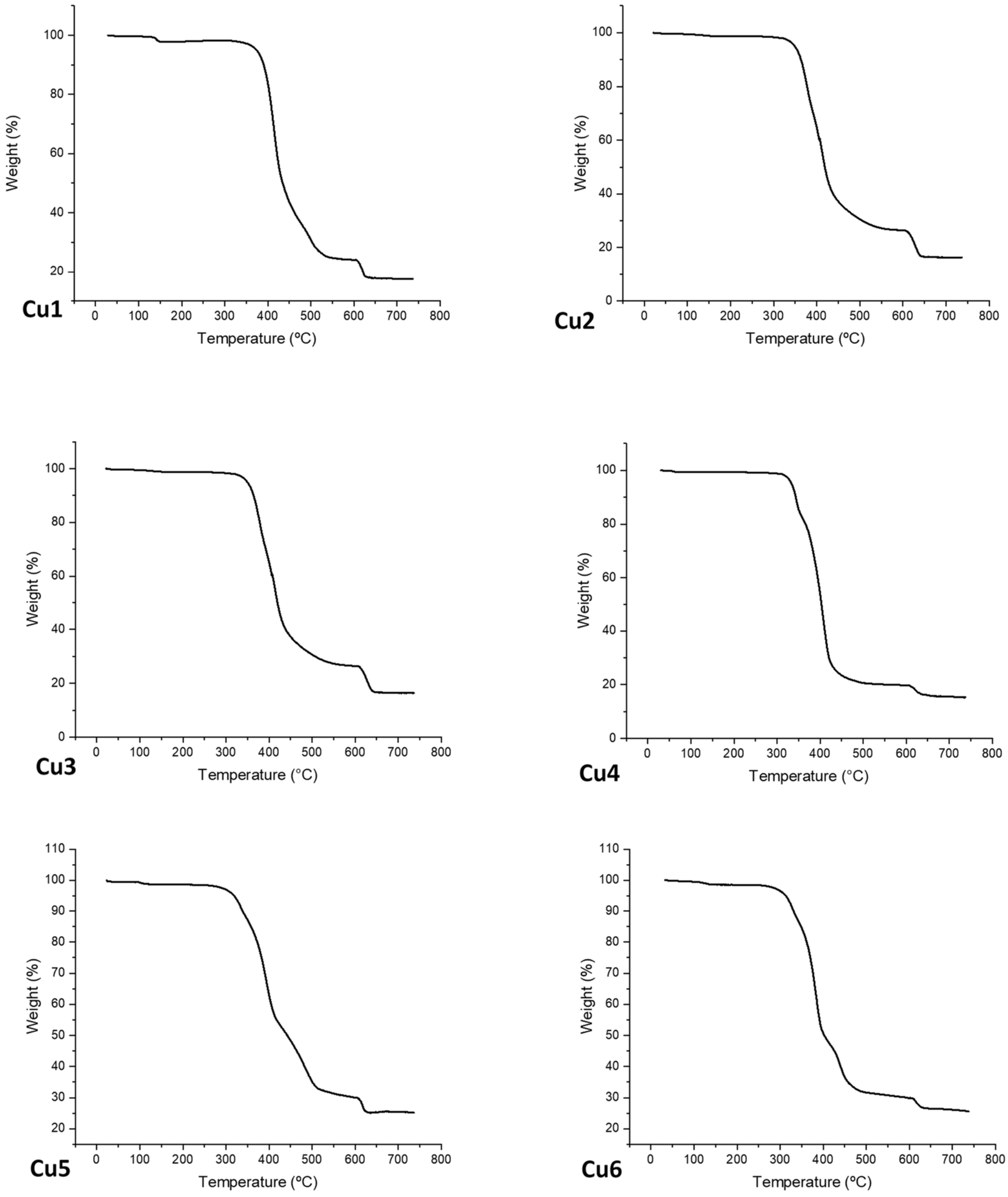

Figure S35. TGA curves of complexes **Cu1-Cu6**.

S5.-EMISSION AND EXCITATION SPECTRA

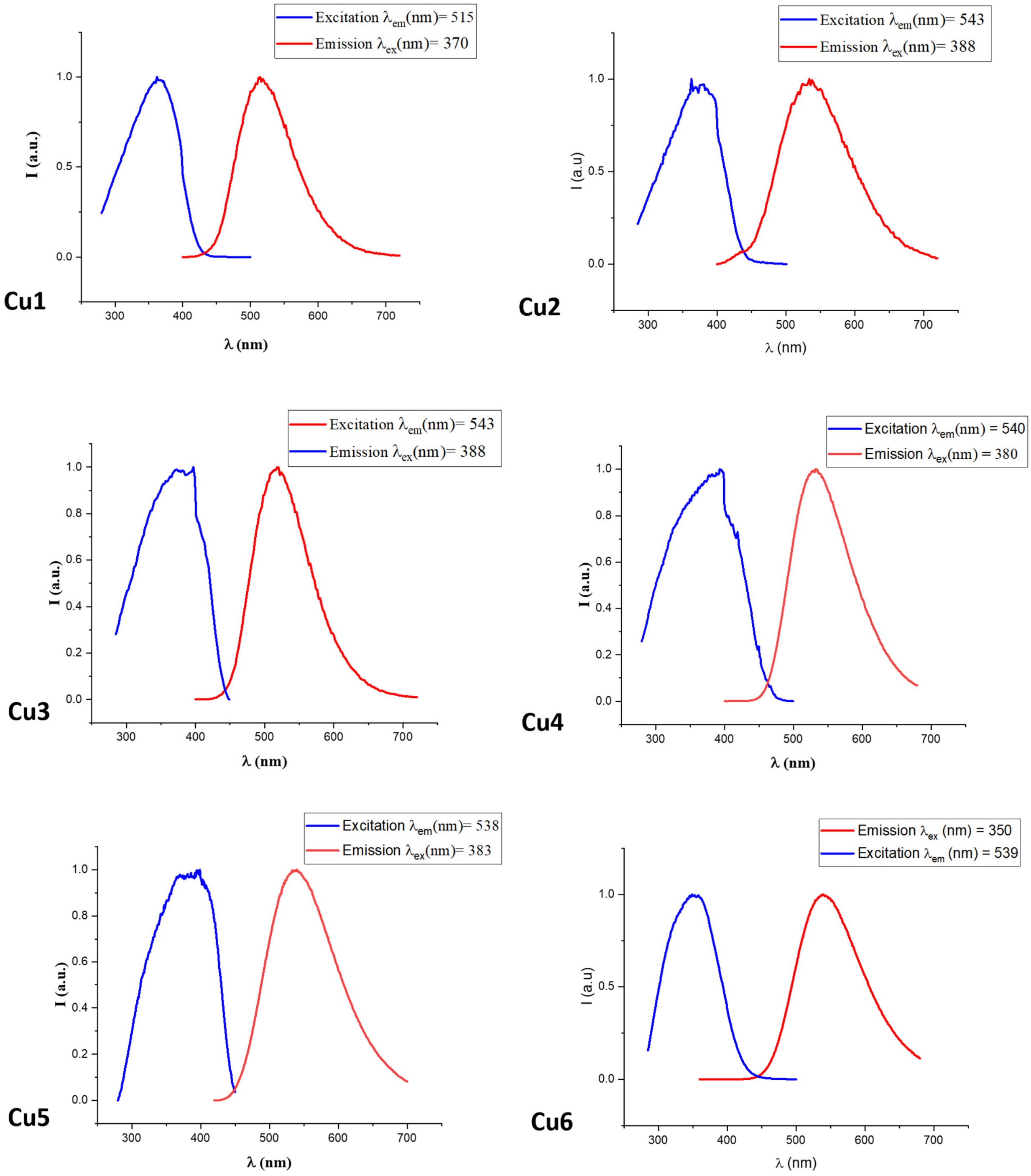

Figure S36. Excitation (blue) and emission (red) spectra in the solid state at room temperature for compounds **Cu1-Cu6**.

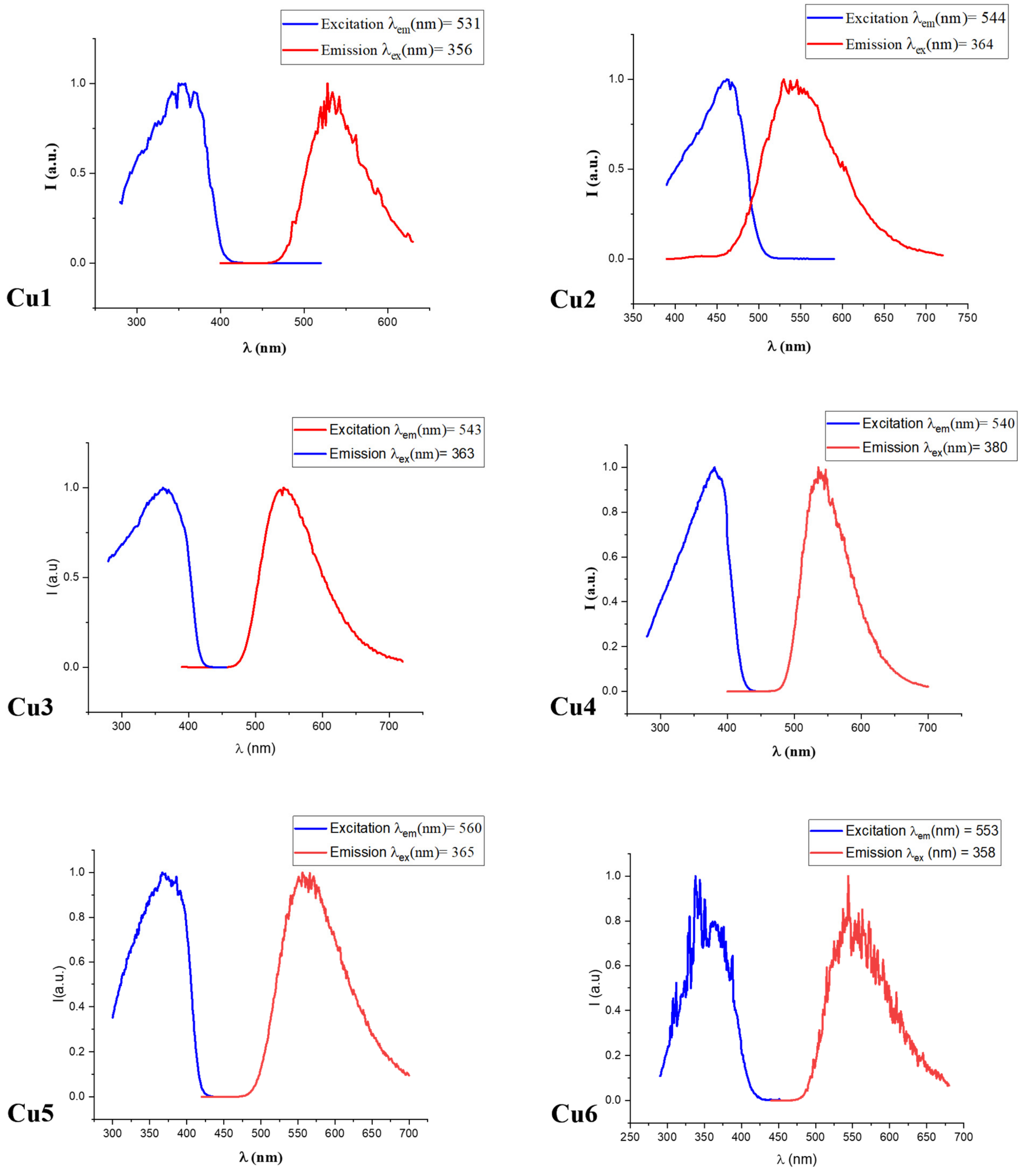

Figure S37. Excitation (blue) and emission (red) in the solid state at 77K for compounds **Cu1-Cu6**.

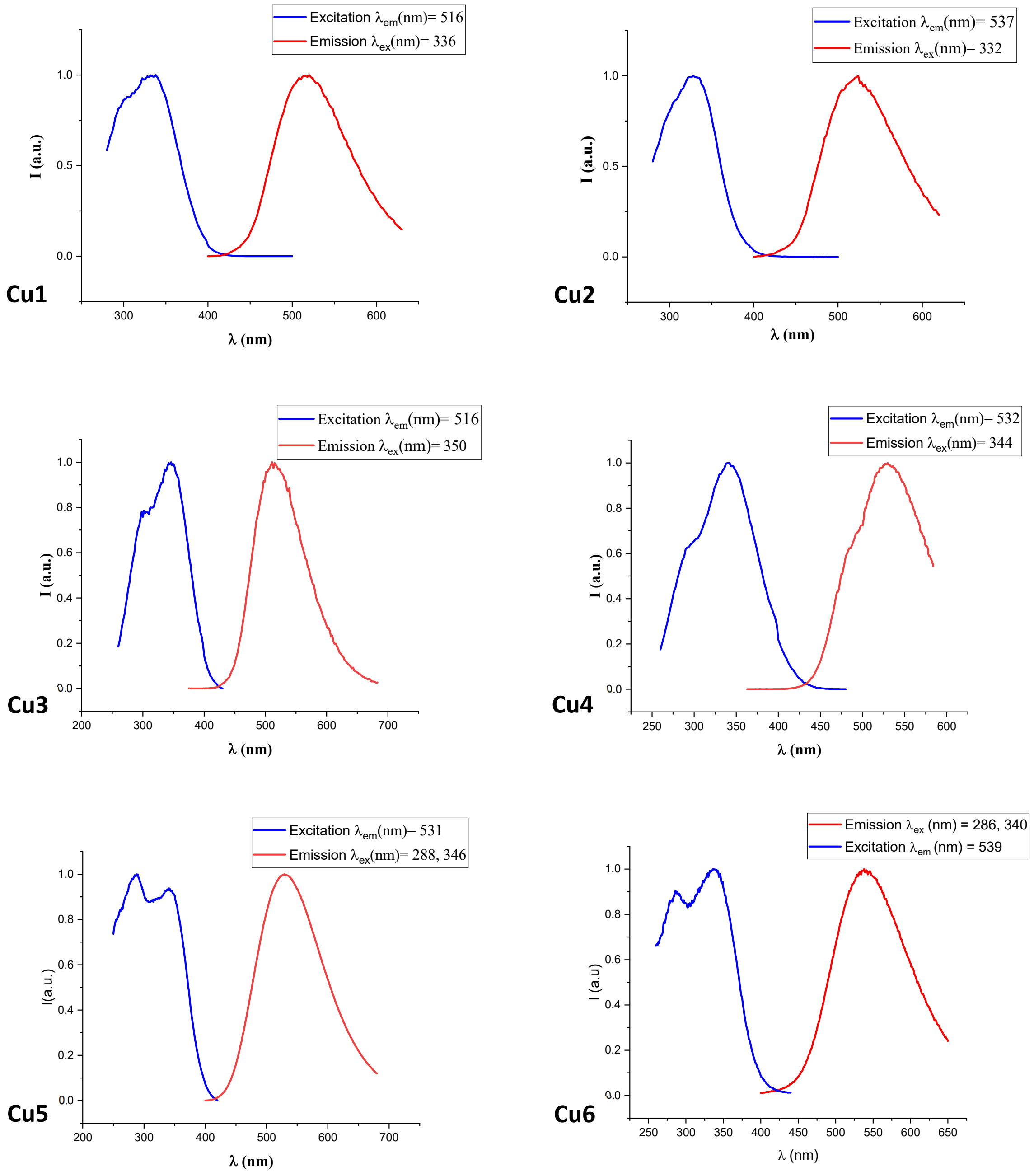

Figure S38. Excitation (blue) and emission (red) in 5wt % PMMA film for compounds **Cu1-Cu6**.

S6.-CIE COORDINATES

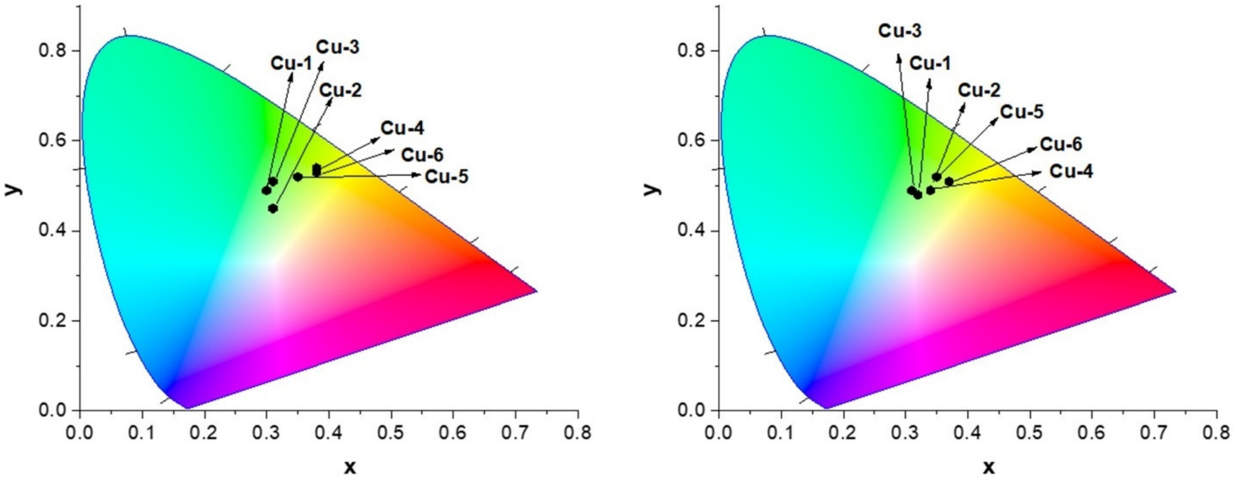

Figure **S39**. Calculated 1931 CIE Colour coordinates for complexes from the quantum yield measurements, after excluding excitation light for solid samples (left) and 5wt % PMMA film (right).

S7.- LIFETIME FITTING CURVES

The fitting program calculates the average lifetimes based on the intensities (I_1 and I_2) according to Equation 1 (see, for example: B. Valeur, M. N. Berberan-Santos. *Molecular Fluorescence. Principles and Applications.* WHILEY-VCH, 2nd Edition. **2013**, p. 23).

$$\text{Equation 1: } \tau(av) = \sum_{\substack{i=1 \\ Ii<0}}^{Nexp} Ii\tau i \Big/ \sum_{\substack{i=1 \\ Ai<0}}^{Nexp} Ii$$

S7.1.-Lifetimes of compounds Cu1-Cu6 in the solid state at 300 K

Measurement Context: Decay

Summary:

Excitation: U pol 405±15nm with PLS-400

Detection: U pol 530±13nm 30000 peak counts

grating 1200/500+

detector UV-red [PMT]

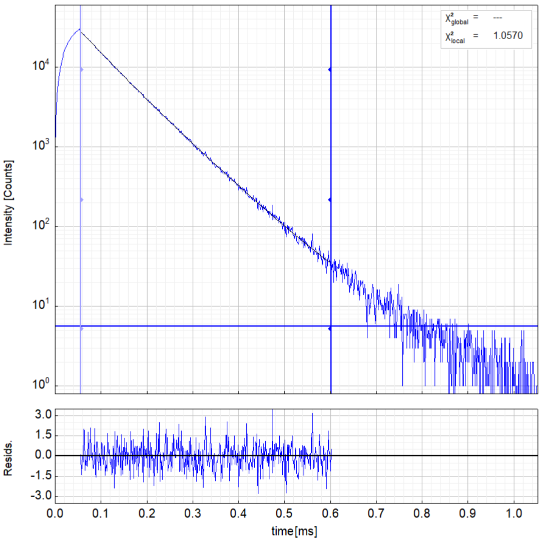

Parameter	Value	Δ	δ
A_1[kCnts/Chnl]	15.1	±4.9	32%
τ_1[ns]	86 800	±4 700	5.4%
I_1[kCnts]	1 030	±270	26%
-			
A_2[kCnts/Chnl]	12.9	±4.9	38%
τ_2[ns]	57 700	±5 300	9.1%
I_2[kCnts]	580	±270	46%
-			
$Bkgr_{Dec}$[kCnts]	0.0056	±0.0033	57%
-			
τ_{AvInt}[ns]	76 210	±350	0.5%
-			

Figure S40. Lifetime and data of compound **Cu1** in the solid state at 300 K.

Measurement Context:	Decay
Summary:	
Excitation:	U pol 405±15nm with PLS-400
Detection:	U pol 540±14nm 10000 peak counts
	grating 1200/500+
	detector UV-red [PMT]

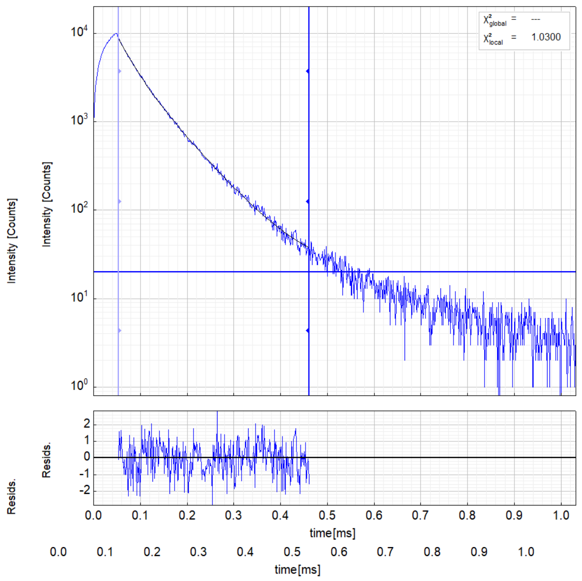

Parameter	Value	Δ	δ
A_1[kCnts/Chnl]	4.63	±0.39	8.3%
τ_1[ns]	73 300	±2 000	2.7%
I_1[kCnts]	265	±14	5.0%
-			
A_2[kCnts/Chnl]	4.07	±0.32	7.7%
τ_2[ns]	29 400	±2 400	8.0%
I_2[kCnts]	94	±15	15%
-			
$Bkgr_{Dec}$[kCnts]	0.0199	±0.0023	11%
-			
τ_{AvInt}[ns]	61 810	±430	0.7%
-			

Figure S41. Lifetime and data of compound **Cu2** in the solid state at 300 K

Measurement Context: Decay

Summary:

Excitation: U pol 405±15nm with PLS-400

Detection: U pol 520±14nm 30000 peak counts

grating 1200/500+

detector UV-red [PMT]

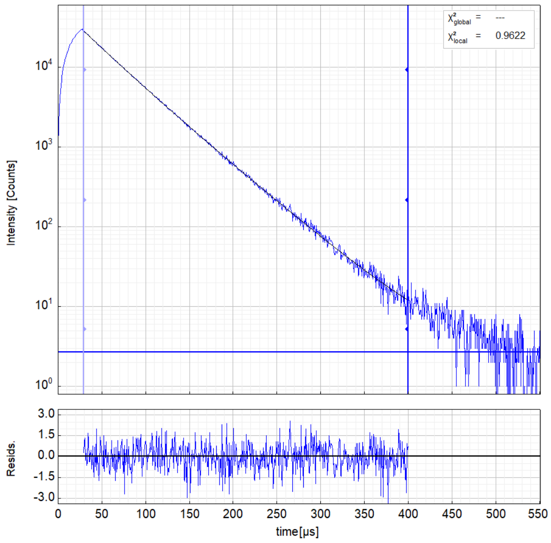

Parameter	Value	Δ	δ
A_1[kCnts/Chnl]	16.8	±5.7	34%
τ_1[ns]	49 300	±2 600	5.1%
I_1[kCnts]	1 290	±370	29%
-			
A_2[kCnts/Chnl]	11.6	±5.6	48%
τ_2[ns]	34 400	±3 200	9.2%
I_2[kCnts]	620	±370	60%
-			
$Bkgr_{Dec}$[kCnts]	0.0028	±0.0018	63%
-			
τ_{AvInt}[ns]	44 430	±110	0.2%
-			

Figure S42. Lifetime and data of compound **Cu3** in the solid state at 300 K

Measurement Context: Decay

Summary:

Excitation: U pol 405±15nm with PLS-400

Detection: U pol 540±14nm 10000 peak counts

grating 1200/500+

detector UV-red [PMT]

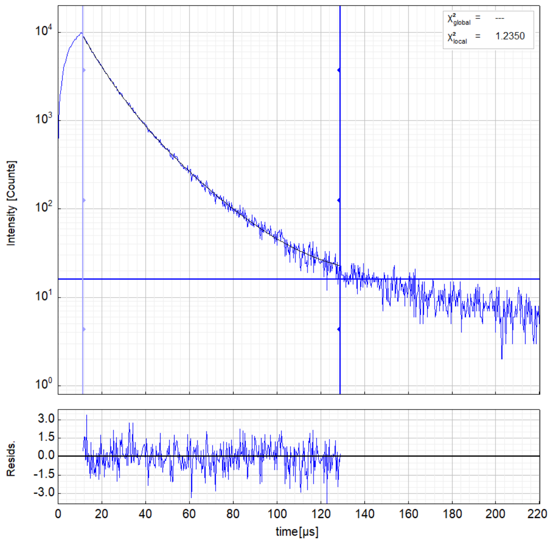

Parameter	Value	Δ	δ
A_1[kCnts/Chnl]	2.67	±0.27	10%
τ_1[ns]	19 690	±570	2.9%
I_1[kCnts]	164	±12	6.7%
-			
A_2[kCnts/Chnl]	6.24	±0.23	3.6%
τ_2[ns]	8 620	±240	2.8%
I_2[kCnts]	168	±11	6.4%
-			
$Bkgr_{Dec}$[kCnts]	0.0160	±0.0016	9.5%
-			
τ_{AvInt}[ns]	14 090	±110	0.8%
-			

Figure S43. Lifetime and data of compound **Cu4** in the solid state at 300 K.

Measurement Context: Decay

Summary:

Excitation: U pol 405±15nm with PLS-400

Detection: U pol 530±14nm 20000 peak counts

grating 1200/500+

detector UV-red [PMT]

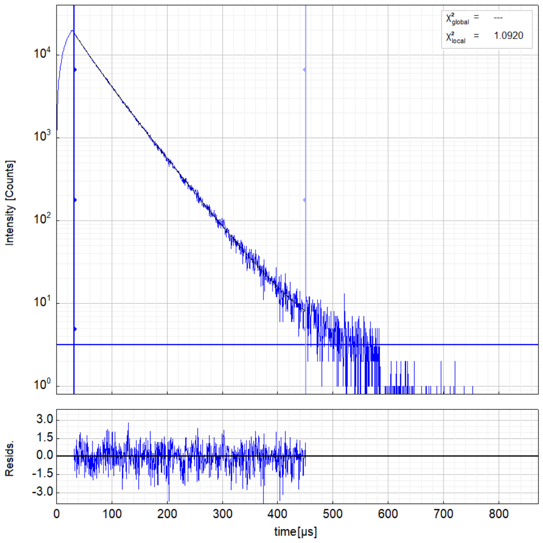

Parameter	Value	Δ	δ
A_1[kCnts/Chnl]	12.30	±0.50	4.0%
τ_1[ns]	53 480	±430	0.8%
I_1[kCnts]	1 028	±34	3.3%
-			
A_2[kCnts/Chnl]	5.97	±0.53	8.9%
τ_2[ns]	31 350	±870	2.8%
I_2[kCnts]	293	±34	12%
-			
$Bkgr_{Dec}$[kCnts]	0.0032	±0.0005	14%
-			
τ_{AvInt}[ns]	48 575	±37	0.1%
-			

Figure S44. Lifetime and data of compound **Cu5** in the solid state at 300 K

Measurement context: Decay

Summary:

Excitation: U pol 378±10nm with PLS-370

Detection: U pol 544±14nm 20000 peak counts

grating 1200/500+

detector UV-red [PMT]

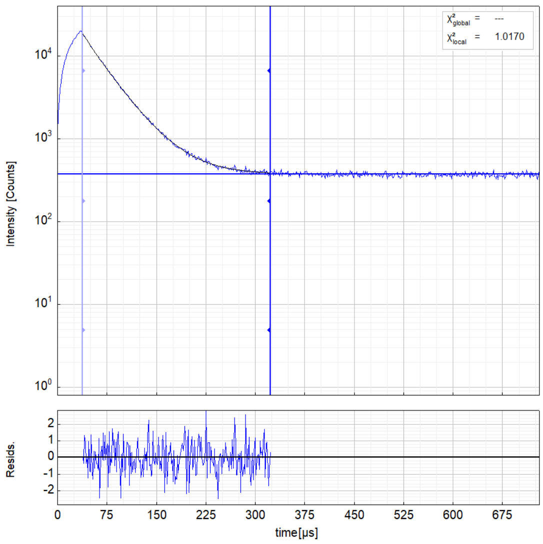

Parameter	Value	Δ	δ
A_1[kCnts/Chnl]	15.6	±1.2	7.2%
τ_1[ns]	40 100	±980	2.4%
I_1[kCnts]	487	±22	4.4%
-			
A_2[kCnts/Chnl]	2.58	±0.90	35%
τ_2[ns]	18 600	±6 600	35%
I_2[kCnts]	38	±24	62%
-			
$Bkgr_{Dec}$[kCnts]	0.3754	±0.0034	0.9%
-			
τ_{AvInt}[ns]	38 570	±210	0.5%
-			

Figure S45. Lifetime and data of compound **Cu6** in the solid state at 300 K.

S7.2.-Lifetimes of compounds Cu1-Cu6 in the solid state at 80 K

Measurement Context: Decay

Summary:

Excitation: U pol 405±15nm with PLS-400

Detection: U pol 535±14nm 10000 peak counts

grating 1200/500+

detector UV-red [PMT]

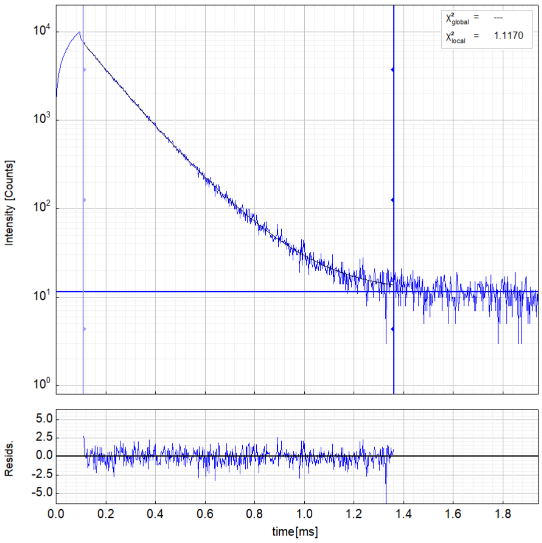

Parameter	Value	Δ	δ
A_1[kCnts/Chnl]	2.0	±2.5	122%
τ_1[ns]	184 000	±30 000	16%
I_1[kCnts]	150	±130	89%
-			
A_2[kCnts/Chnl]	5.6	±2.4	43%
τ_2[ns]	116 000	±18 000	15%
I_2[kCnts]	250	±130	52%
-			
$Bkgr_{Dec}$[kCnts]	0.0114	±0.0022	19%
-			
τ_{AvInt}[ns]	140 300	±2 000	1.4%
-			

Figure S46. Lifetime and data of compound **Cu1** in the solid state at 80 K.

Measurement context: Decay

Summary:

Excitation: U pol 405±15nm with PLS-400

Detection: U pol 540±14nm 10000 peak counts

grating 1200/500+

detector UV-red [PMT]

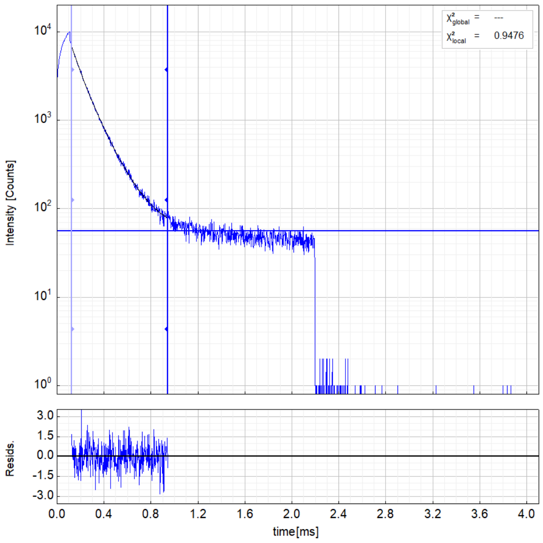

Parameter	Value	Δ	δ
A_1[kCnts/Chnl]	3.70	±0.84	23%
τ_1[ns]	161 000	±17 000	10.0%
I_1[kCnts]	232	±35	15%
-			
A_2[kCnts/Chnl]	2.98	±0.83	28%
τ_2[ns]	78 600	±9 400	12%
I_2[kCnts]	92	±38	41%
-			
$Bkgr_{Dec}$[kCnts]	0.0557	±0.0062	11%
-			
τ_{AvInt}[ns]	137 300	±2 900	2.1%
-			

Figure S47. Lifetime and data of compound **Cu2** in the solid state at 80 K.

Measurement context: Decay

Summary:

Excitation: U pol 380±14nm with Xe-Lamp

Detection: U pol 555±14nm 30000 peak counts

grating 1200/500+

detector UV-red [PMT]

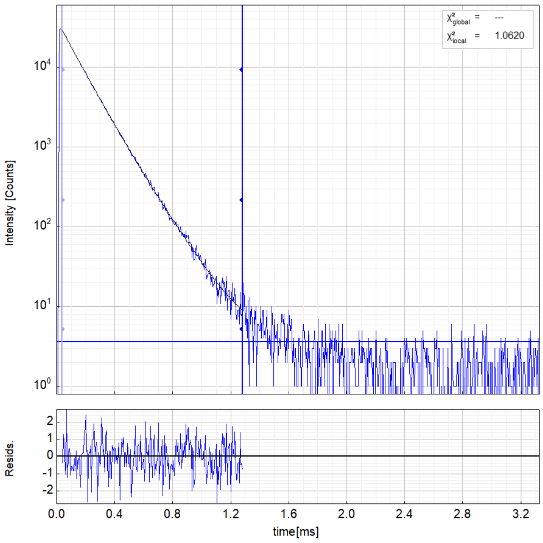

Parameter	Value	Δ	δ
A_1[kCnts/Chnl]	10.7	±1.6	15%
τ_1[ns]	162 000	±3 200	2.0%
I_1[kCnts]	338	±47	14%
-			
A_2[kCnts/Chnl]	18.1	±1.5	8.1%
τ_2[ns]	112 500	±4 600	4.1%
I_2[kCnts]	397	±47	12%
-			
$Bkgr_{Dec}$[kCnts]	0.0037	±0.0012	32%
-			
τ_{AvInt}[ns]	135 250	±250	0.2%
-			

Figure S48. Lifetime and data of compound **Cu3** in the solid state at 80 K.

Measurement context: Decay

Summary:

Excitation: U pol 405±15nm with PLS-400

Detection: U pol 540±14nm 10000 peak counts

grating 1200/500+

detector UV-red [PMT]

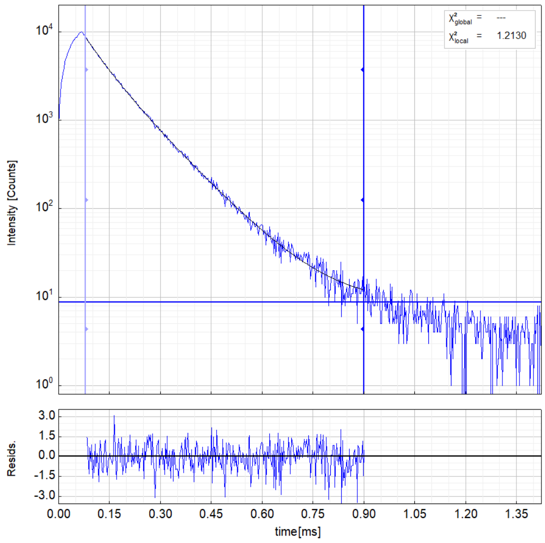

Parameter	Value	Δ	δ
A_1[kCnts/Chnl]	4.71	±0.48	10%
τ_1[ns]	112 900	±3 900	3.4%
I_1[kCnts]	208	±15	7.0%
-			
A_2[kCnts/Chnl]	3.61	±0.46	13%
τ_2[ns]	56 500	±3 500	6.1%
I_2[kCnts]	80	±15	19%
-			
$Bkgr_{Dec}$[kCnts]	0.0088	±0.0014	15%
-			
τ_{AvInt}[ns]	97 270	±450	0.5%
-			

Figure S49. Lifetime and data of compound **Cu4** in the solid state at 80 K.

Measurement context: Decay

Summary:

Excitation: U pol 405±15nm with PLS-400

Detection: U pol 555±14nm 20000 peak counts

grating 1200/500+

detector UV-red [PMT]

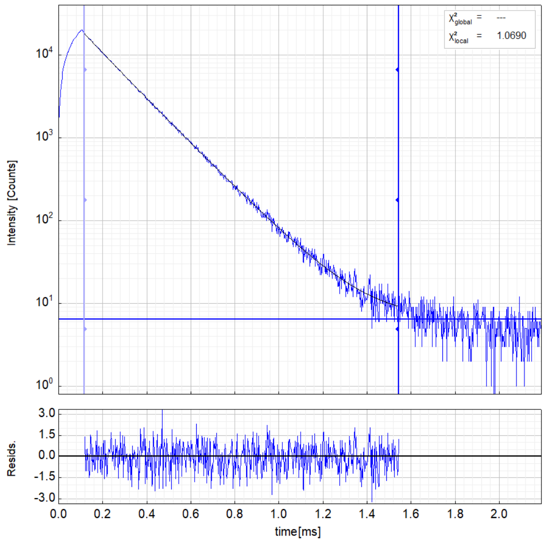

Parameter	Value	Δ	δ
A_1[kCnts/Chnl]	15.5	±1.3	7.8%
τ_1[ns]	165 200	±2 600	1.6%
I_1[kCnts]	994	±61	6.1%
-			
A_2[kCnts/Chnl]	2.7	±1.2	44%
τ_2[ns]	109 000	±18 000	16%
I_2[kCnts]	112	±61	55%
-			
$Bkgr_{Dec}$[kCnts]	0.0065	±0.0007	10%
-			
τ_{AvInt}[ns]	159 450	±280	0.2%
-			

Figure S50. Lifetime and data of compound **Cu5** in the solid state at 80 K.

Measurement Context: Decay

Summary:

Sample:	T.V-77K
Solvent:	<unspecified>
Excitation:	U pol 350±5nm with Xe-Lamp
Detection:	U pol 540±5nm 50000 peak counts
	grating 1200/500+
	detector UV-red [PMT]

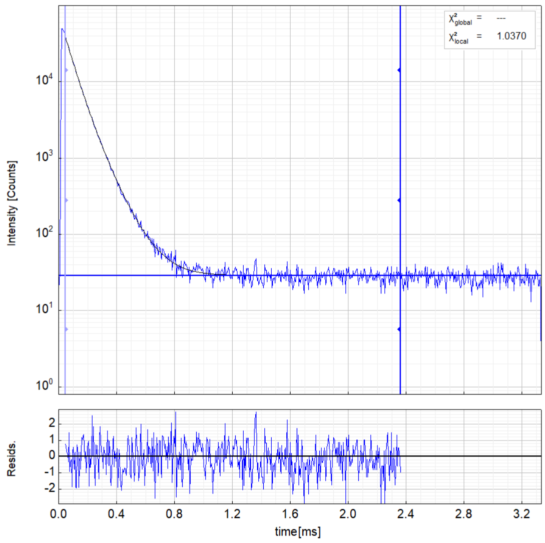

Parameter	Value	Δ	δ
A_1[kCnts/Chnl]	8.2	±1.4	17%
τ_1[ns]	116 000	±4 300	3.7%
I_1[kCnts]	185	±23	12%
-			
A_2[kCnts/Chnl]	31.3	±1.4	4.4%
τ_2[ns]	61 600	±1 300	2.0%
I_2[kCnts]	376	±24	6.3%
-			
$Bkgr_{Dec}$[kCnts]	0.0290	±0.0004	1.3%
-			
τ_{AvInt}[ns]	79 500	±230	0.3%
-			

Figure S51. Lifetime and data of compound **Cu6** in the solid state at 80 K.

S7.3.-Lifetimes of compounds Cu1-Cu6 in 5wt % PMMA film at r.t

Measurement Context: Decay

Summary:

Excitation: U pol 336±5nm with Xe-Lamp

Detection: U pol 516±5nm 10000 peak counts

grating 1200/500+

detector UV-red [PMT]

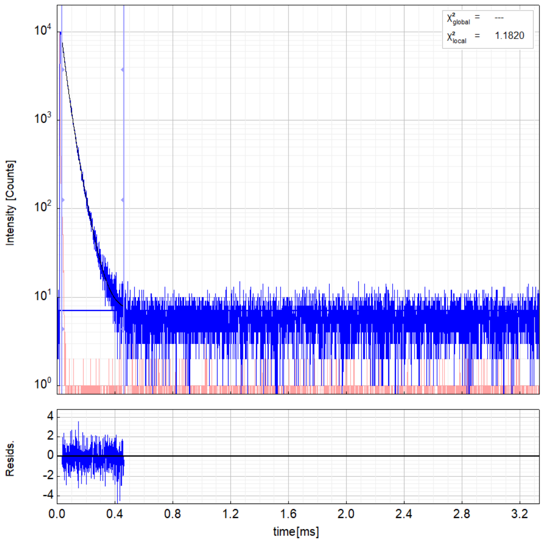

Parameter	Value	Δ	δ
A_1[kCnts/Chnl]	2.76	±0.21	7.3%
τ_1[ns]	52 530	±880	1.7%
I_1[kCnts]	226	±13	5.6%
-			
A_2[kCnts/Chnl]	5.07	±0.17	3.2%
τ_2[ns]	26 790	±660	2.4%
I_2[kCnts]	212	±12	5.6%
-			
$Bkgr_{Dec}$[kCnts]	0.0071	±0.0003	3.9%
-			
τ_{AvInt}[ns]	40 070	±70	0.2%
-			

Figure S52. Lifetime and data of compound **Cu1** in 5wt % PMMA film at r.t

Measurement Context: Decay

Summary:

Excitation: U pol 332±5nm with Xe-Lamp

Detection: U pol 537±5nm 100000 peak counts

grating 1200/500+

detector UV-red [PMT]

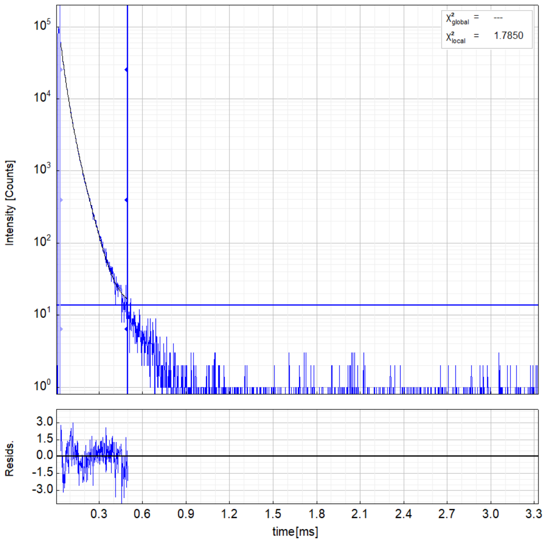

Parameter	Value	Δ	δ
A_1[kCnts/Chnl]	14.70	±0.67	4.5%
τ_1[ns]	54 340	±530	1.0%
I_1[kCnts]	312.0	±9.3	3.0%
-			
A_2[kCnts/Chnl]	44.53	±0.54	1.2%
τ_2[ns]	24 080	±150	0.6%
I_2[kCnts]	418.8	±9.7	2.3%
-			
$Bkgr_{Dec}$[kCnts]	0.0137	±0.0008	5.6%
-			
τ_{AvInt}[ns]	36 993	±67	0.2%
-			

Figure S53. Lifetime and data of compound **Cu2** in 5wt % PMMA film at r.t

Measurement context: Decay

Summary:

Excitation: U pol 378±10nm with PLS-370

Detection: U pol 514±14nm 10000 peak counts

grating 1200/500+

detector UV-red [PMT]

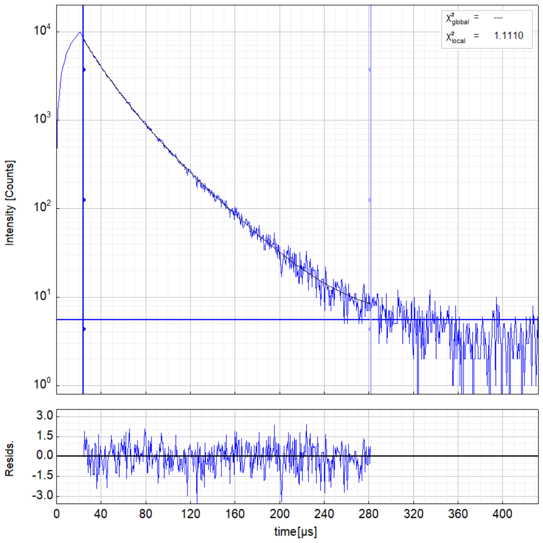

Parameter	Value	Δ	δ
A_1[kCnts/Chnl]	3.28	±0.35	11%
τ_1[ns]	36 500	±1 200	3.3%
I_1[kCnts]	187	±13	6.9%
-			
A_2[kCnts/Chnl]	4.86	±0.33	6.8%
τ_2[ns]	16 720	±630	3.7%
I_2[kCnts]	127	±14	11%
-			
$Bkgr_{Dec}$[kCnts]	0.0056	±0.0007	12%
-			
τ_{AvInt}[ns]	28 470	±150	0.5%
-			

Figure S54. Lifetime and data of compound **Cu3** in 5wt % PMMA film at r.t.

Measurement Context: Decay

Summary:

Excitation: U pol 378±10nm with PLS-370

Detection: U pol 532±14nm 10000 peak counts

grating 1200/500+

detector UV-red [PMT]

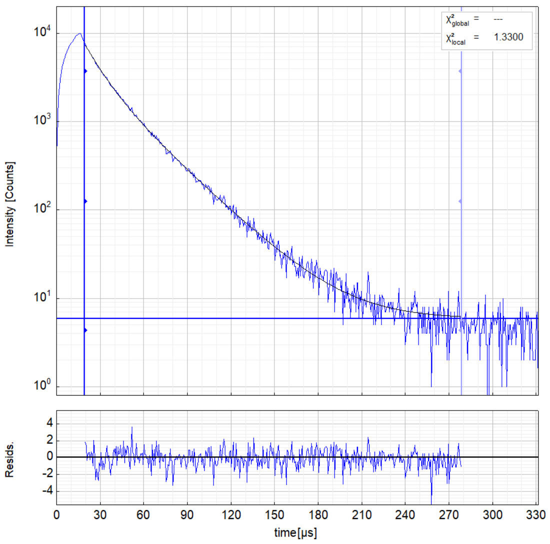

Parameter	Value	Δ	δ
A_1[kCnts/Chnl]	3.32	±0.19	5.7%
τ_1[ns]	28 150	±560	2.0%
I_1[kCnts]	145.8	±5.2	3.6%
-			
A_2[kCnts/Chnl]	3.97	±0.17	4.2%
τ_2[ns]	11 230	±570	5.0%
I_2[kCnts]	69.5	±5.8	8.2%
-			
$Bkgr_{Dec}$[kCnts]	0.0059	±0.0002	2.4%
-			
τ_{AvInt}[ns]	22 687	±95	0.4%
-			

Figure S55. Lifetime of compounds **Cu4** in 5wt % PMMA film at r.t.

Measurement Context: Decay

Summary:

Sample: RJS-100-TV-FILMS

Solvent: <unspecified>

Excitation: U pol 285±12nm with PLS-280

Detection: U pol 517±14nm 10000 peak counts

grating 1200/500+

detector UV-red [PMT]

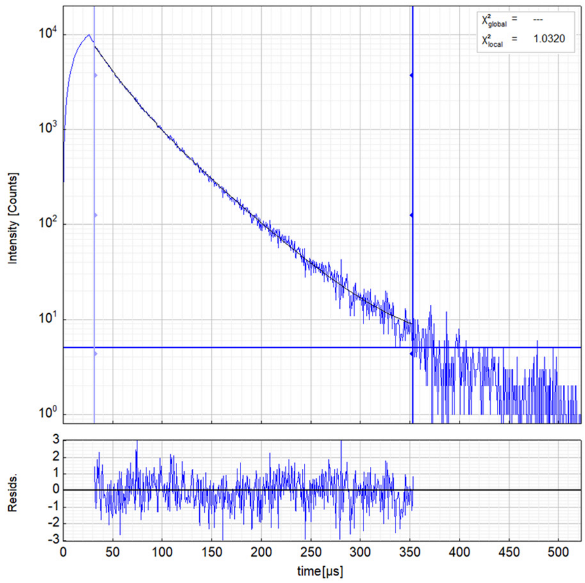

Parameter	Value	Δ	δ
A_1[kCnts/Chnl]	3.39	±0.11	3.1%
τ_1[ns]	47 540	±470	1.0%
I_1[kCnts]	251.3	±6.0	2.4%
A_2[kCnts/Chnl]	4.39	±0.11	2.3%
τ_2[ns]	21 510	±440	2.0%
I_2[kCnts]	147.5	±5.6	3.7%
$Bkgr_{Dec}$[kCnts]	0.0050	±0.0004	6.1%
τ_{AvInt}[ns]	37 909	±55	0.1%

Figure S56. Lifetime of compounds **Cu5** in 5wt % PMMA film at r.t.

Measurement Context: Decay

Summary:

Excitation: U pol 378±10nm with PLS-370

Detection: U pol 544±14nm 10000 peak counts

grating 1200/500+

detector UV-red [PMT]

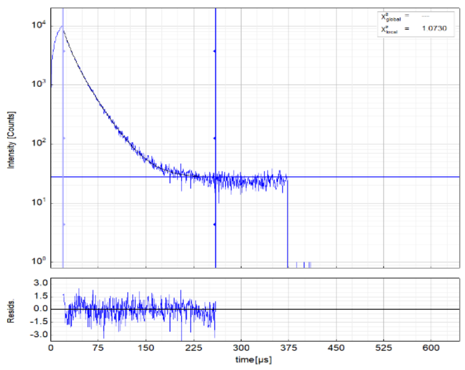

Parameter	Value	Δ	δ
A_1[kCnts/Chnl]	4.039	±0.092	2.3%
τ_1[ns]	26 190	±240	0.9%
I_1[kCnts]	165.3	±2.4	1.5%
-			
A_2[kCnts/Chnl]	4.42	±0.12	2.5%
τ_2[ns]	10 120	±150	1.5%
I_2[kCnts]	69.8	±2.9	4.0%
-			
$Bkgr_{Dec}$[kCnts]	0.0274	±0.0005	1.6%
-			
τ_{AvInt}[ns]	21 416	±55	0.3%
-			

Figure S57. Lifetime of compounds **Cu6** in 5wt % PMMA film at r.t.

S8.-LIFETIME STUDIES AT DIFFERENT TEMPERATURES

Equation1: $\tau = \dfrac{3 + e^{-\Delta E(S_1\text{-}T_1)/K_BT}}{3\left(\frac{1}{\tau(T_1)}\right) + \left(\frac{1}{\tau(S_1)}\right)e^{-\Delta E(S_1\text{-}T_1)/K_BT}}$

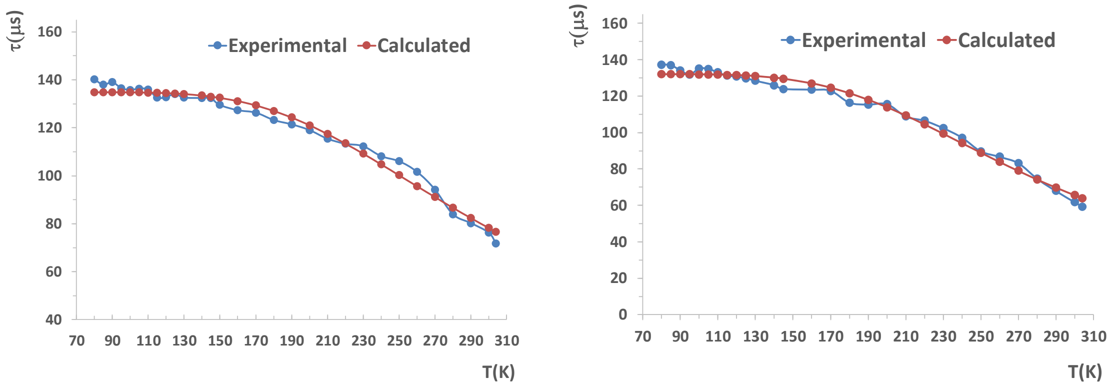

Figure **S58**. Temperature dependence of the emission lifetime of complex **Cu1** (left, Chi-Square 0.976) and **Cu2** (right, Chi-Square 0.983) in the solid state and the corresponding fitting values using Equation 1.

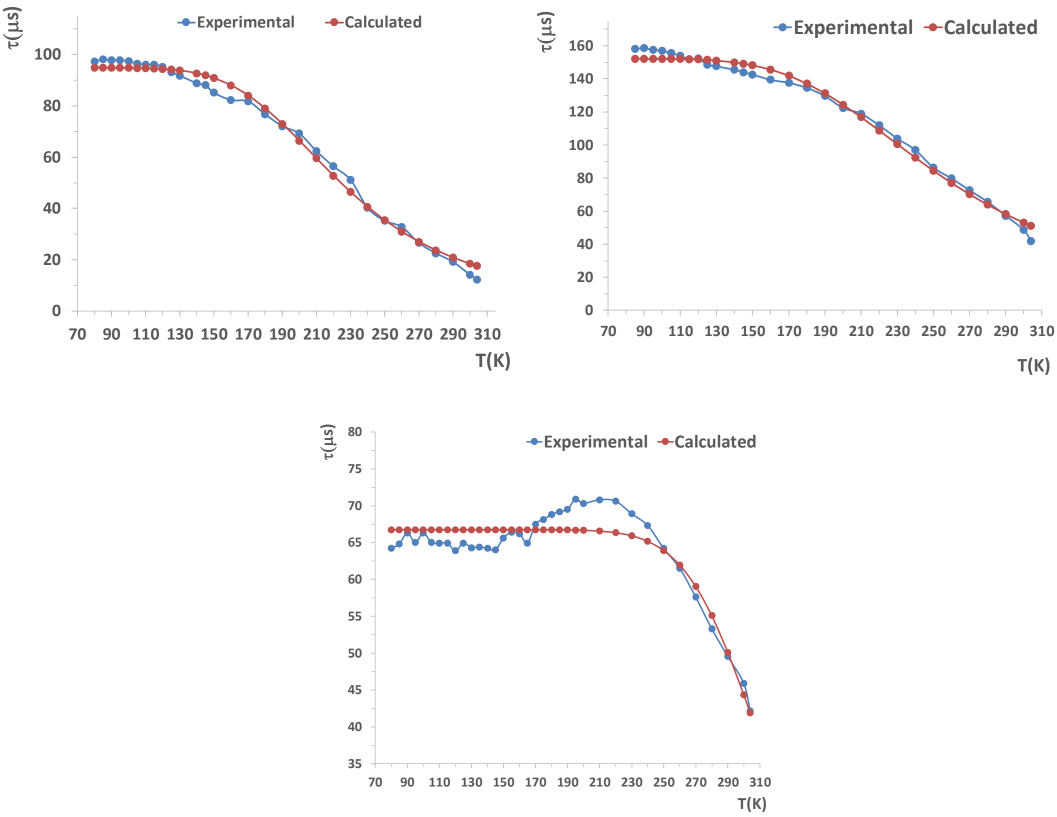

Figure **S59**. Temperature dependence of the emission lifetime of complex **Cu4** (up left, Chi-Square 0.990) and **Cu5** (up right, Chi-Square 0.987) and **Cu6** (down left, Chi-Square 0.885) in the solid state and the corresponding fitting values using Equation 1.

S9- BURIED VOLUME PERCENTAGE.

[P^P %Vbur] *[N^N %Vbur]* *[Total %Vbur]*

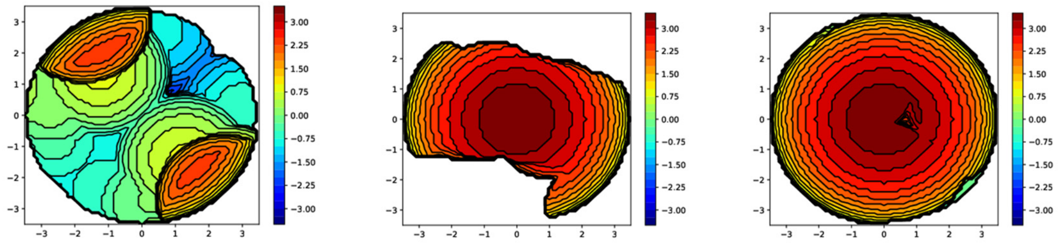

Figure S60. Topological maps for the diphosphane, carbene and both ligands together in **Cu1**.

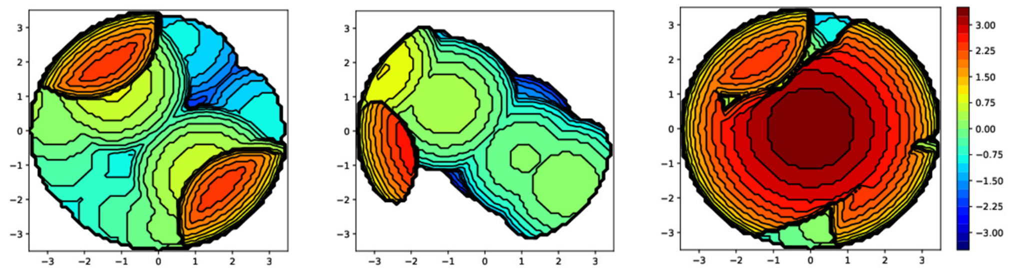

Figure S61. Topological maps for the diphosphane, carbene and both ligands together in **Cu2**.

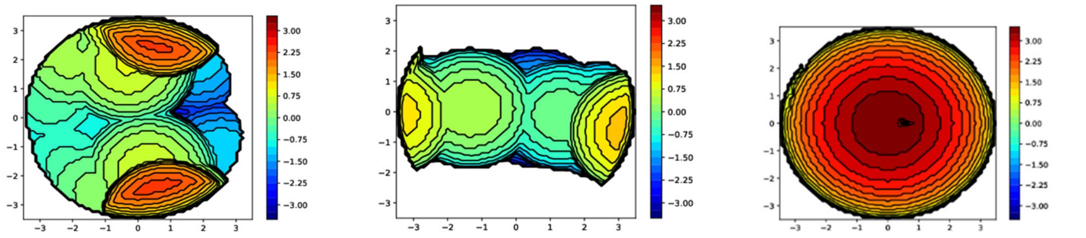

Figure S62. Topological maps for the diphosphane, carbene and both ligands together in **Cu3**.

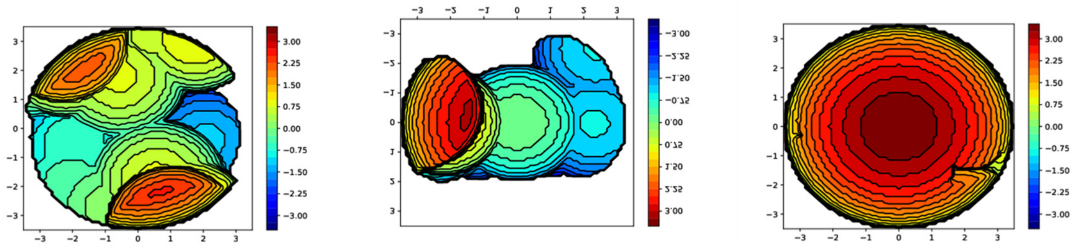

Figure S63. Topological maps for the diphosphane, carbene and both ligands together in **Cu5**.

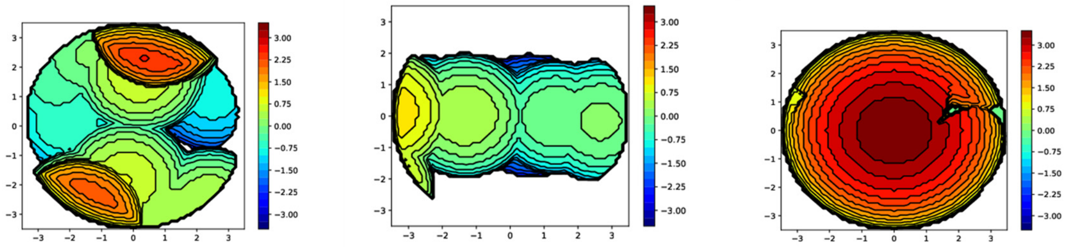

Figure S64. Topological maps for the diphosphane, carbene and both ligands together in $[Cu(L_A)(Dpephos)]PF_6$ (**I**).

S10.- VOLTAMETRIES

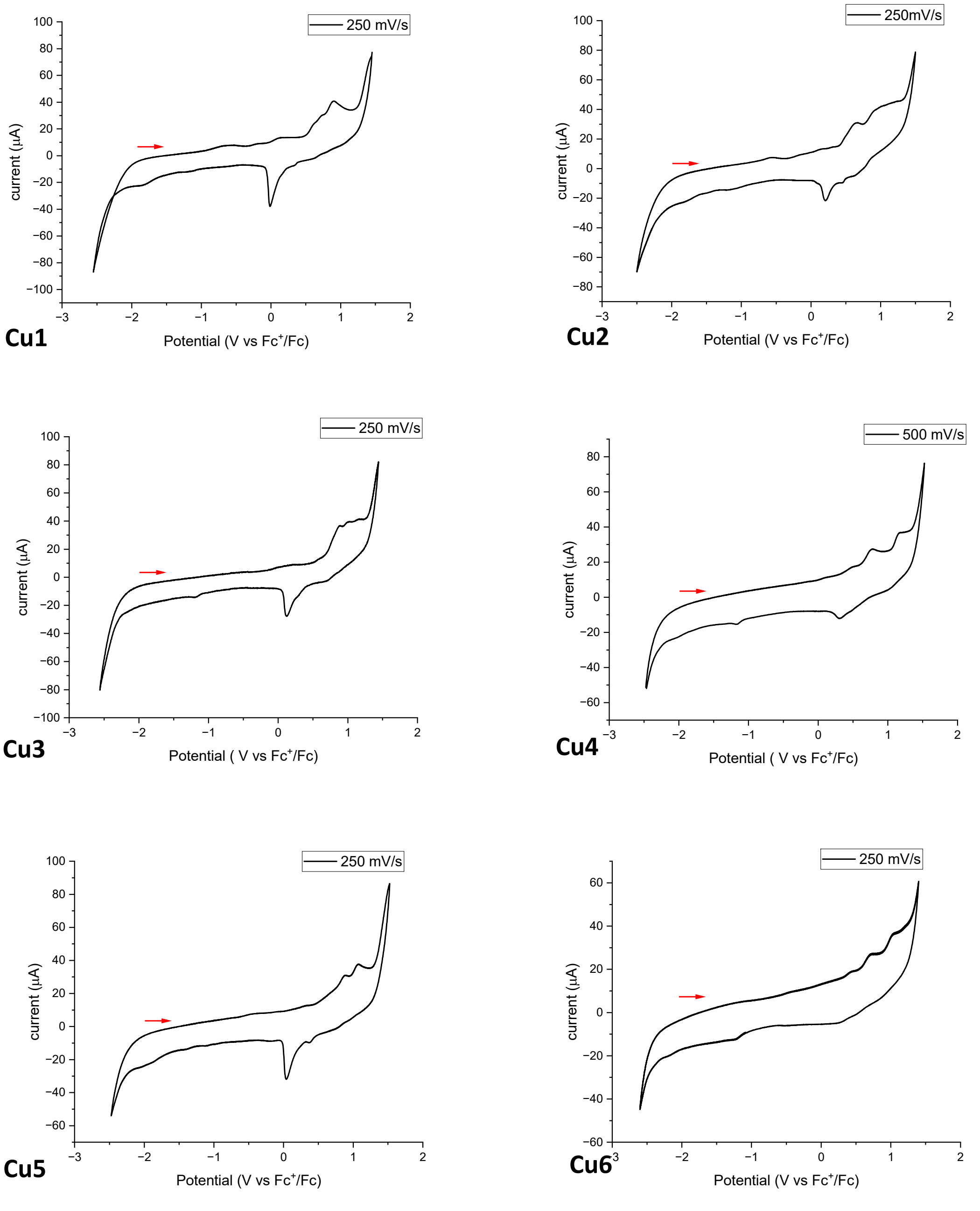

Figure S65. Voltametries of compounds **Cu1-Cu6** in the range -3 to 2 V at 250 mV/s, starting at 0 V.

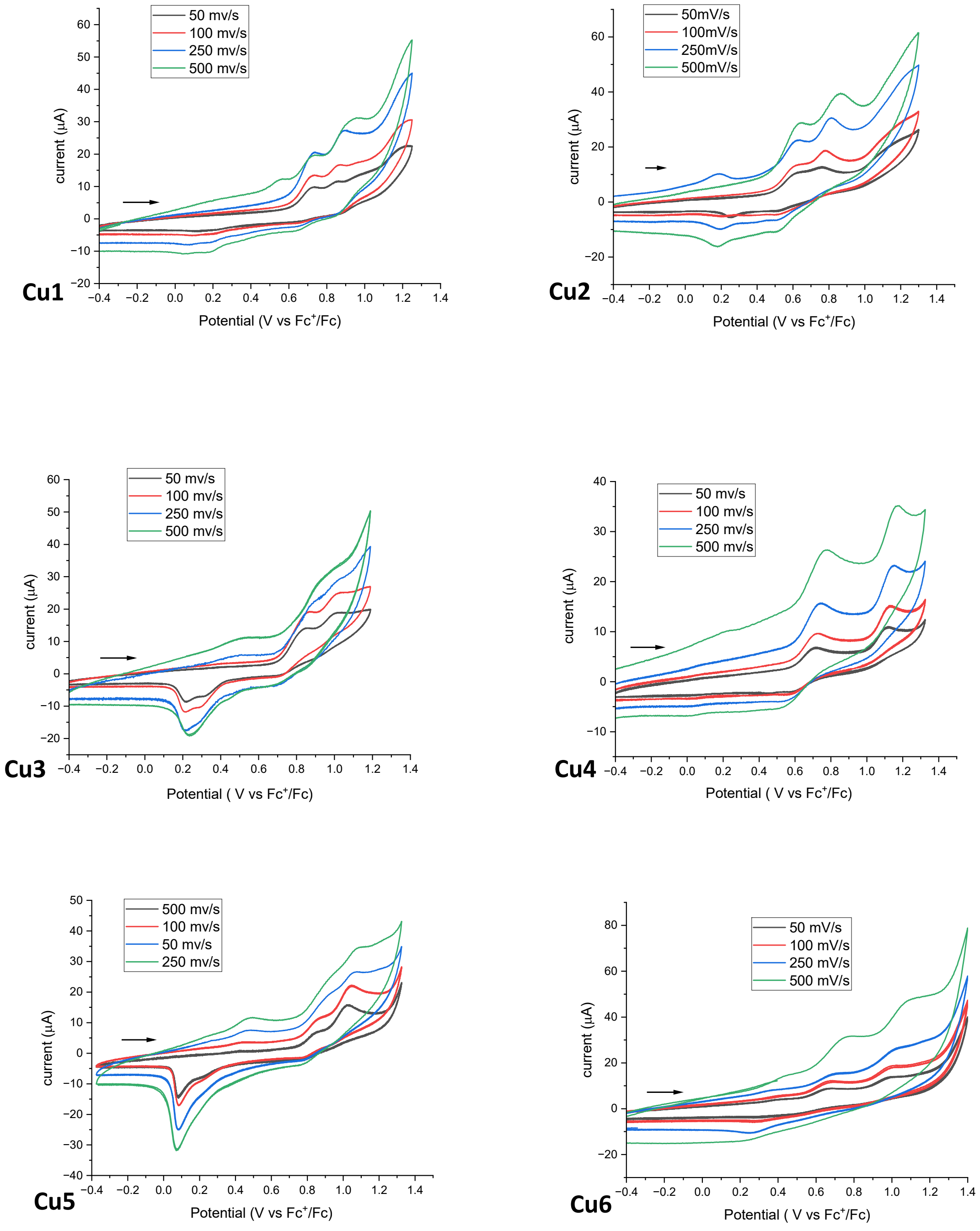

Figure S66. Voltametries of compounds **Cu1-Cu6** at different rates starting at 0 V.
